# Electro‐Olefination—A Catalyst Free Stereoconvergent Strategy for the Functionalization of Alkenes

**DOI:** 10.1002/chem.202001394

**Published:** 2020-06-25

**Authors:** Andreas N. Baumann, Arif Music, Jonas Dechent, Nicolas Müller, Thomas C. Jagau, Dorian Didier

**Affiliations:** ^1^ Department of Chemistry and Pharmacy Ludwig-Maximilians-University Munich Butenandtstraße 5–13, Haus F 81377 Munich Germany

**Keywords:** catalyst-free, electrochemistry, olefination, organoborates, stereoconvergent

## Abstract

Conventional methods carrying out C(sp^2^)−C(sp^2^) bond formations are typically mediated by transition‐metal‐based catalysts. Herein, we conceptualize a complementary avenue to access such bonds by exploiting the potential of electrochemistry in combination with organoboron chemistry. We demonstrate a transition metal catalyst‐free electrocoupling between (hetero)aryls and alkenes through readily available alkenyl‐tri(hetero)aryl borate salts (ATBs) in a stereoconvergent fashion. This unprecedented transformation was investigated theoretically and experimentally and led to a library of functionalized alkenes. The concept was then carried further and applied to the synthesis of the natural product pinosylvin and the derivatization of the steroidal dehydroepiandrosterone (DHEA) scaffold.

## Graphical abstract

**Electro‐Olefination**: The electrocoupling between (hetero)aryls and alkenes by alkenyl‐triaryl borates (ATBs) is demonstrated. ATBs are synthesized by a triple ligand exchange reaction on potassium alkenyltrifluroborates with organometallic species and directly oxidized in an electrochemical two‐pot coupling towards functionalized alkenes in a stereoconvergent fashion.

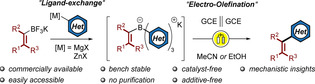

Despite its young history of only a few decades, the Suzuki–Miyaura reaction is one of the most utilized reactions in modern organic chemistry.^1^, ^2^ The palladium‐catalyzed coupling of boronic acids with organohalides was not only awarded with the Nobel prize in 2010, in fact, a recent study ranks the Suzuki–Miyaura coupling as one of the most frequently used reactions (5th place) in medicinal chemistry.^1^ Besides, many other transition‐metal‐mediated cross‐couplings, namely Stille, Heck, Negishi, Sonogashira, Hiyama and Kumada are likewise powerful tools to forge new C−C bonds.^3^ Such indispensable strategies undoubtedly display many advantages and have inspired us to challenge the formation of C−C bonds without the need of the commonly used transition‐metal catalysts, thus breaking new grounds in the field of cross‐coupling reactions. We first started our ambitious concept by replacing the catalyst with an electrochemical setup. Innate advantages, including the use of inexpensive and reusable electrodes, reaction tuneability and scalability do not only rely on the modern and cutting‐edge work from Baran, but trace back to many other advances in electrochemical synthesis since the pioneering works of Volta and Faraday in the 19th century.^4^

We already employed electrochemistry to initiate aryl–aryl bond formation, inspired by the work of Geske^5^ and Waldvogel^6^ (Scheme 1 A), introducing new hetero‐substituted tetraarylborate salts (TABs). We demonstrated that the formation of „unsymmetrical“ TAB salts is enabled by a triple ligand exchange reaction on commercially available organotrifluroborate species employing aryl‐Grignard reagents. Submitting those TABs to mild electrochemical oxidation led to the selective formation of heterocoupled biaryls (Scheme 1 B).^7^

**Scheme 1:**
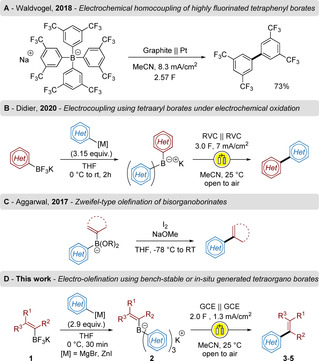
Electrochemical/ Transition‐metal catalyst‐free C−C couplings.

As an alternative route to conventional cross coupling reactions, the catalyst free Zweifel olefination cannot be neglected.^8^ This powerful methodology enables the stereospecific formation of alkenes from the corresponding alkenyl‐organoborinates, as exemplified recently by the groups of Aggarwal and Morken (Scheme 1 C).^9^ In addition, we demonstrated that the logical combination of different organometallic reagents^10^ with boron alkoxides could lead to the formation of the required bis‐organoborinates in an efficient one‐pot process.^11^ Based on these findings, we decided to examine the reactivity of alkenyl‐triaryl borate salts (ATBs) to develop an electro‐olefination reaction (Scheme 1 D).

ATBs (**2**) are underexplored salts, the only representative compound being triphenylvinyl borate which can be synthesized by treatment of tetravinyltin with triphenylborane.^12^ To investigate the electro‐olefination and expand the structural variety of ATBs, we aimed to simplify their access. Therefore, we built on our previously described strategy for the synthesis of hetero‐substituted tetraarylborate salts (TABs), and decided to make ATBs accessible by a triple ligand exchange reaction onto the corresponding potassium alkenyl‐trifluoro borates **1** (Molander salts),^13^ employing ex situ generated Grignard or organozinc reagents.^14^

We anticipated that the removal of an electron through an oxidation process should occur preferentially on the alkenyl moiety, avoiding the energetically disfavored dearomatization of one of the aryl groups. As a proof of concept, we first synthesized the model systems **2 a** 15 and **2 b**, possessing, respectively *para*‐fluorophenyl and phenyl moieties in addition to the β‐styryl substituent (Figure 1). To describe the change in the electronic structure upon oxidation of **2 a** and **2 b**, spin and charge densities were computed based on Mulliken population analysis of the DFT results. Charge densities were additionally computed using the CHarges from ELectrostatic Potentials using a Grid‐based (ChElPG) method.^14^ Blue areas (Figure 1 A) represent positive spin densities after oxidation. Only the alkenyl substituent is selectively oxidized in both cases whereas the charge and spin densities of the other aromatic substituents only change insignificantly, confirming our assumptions.

**Figure 1:**
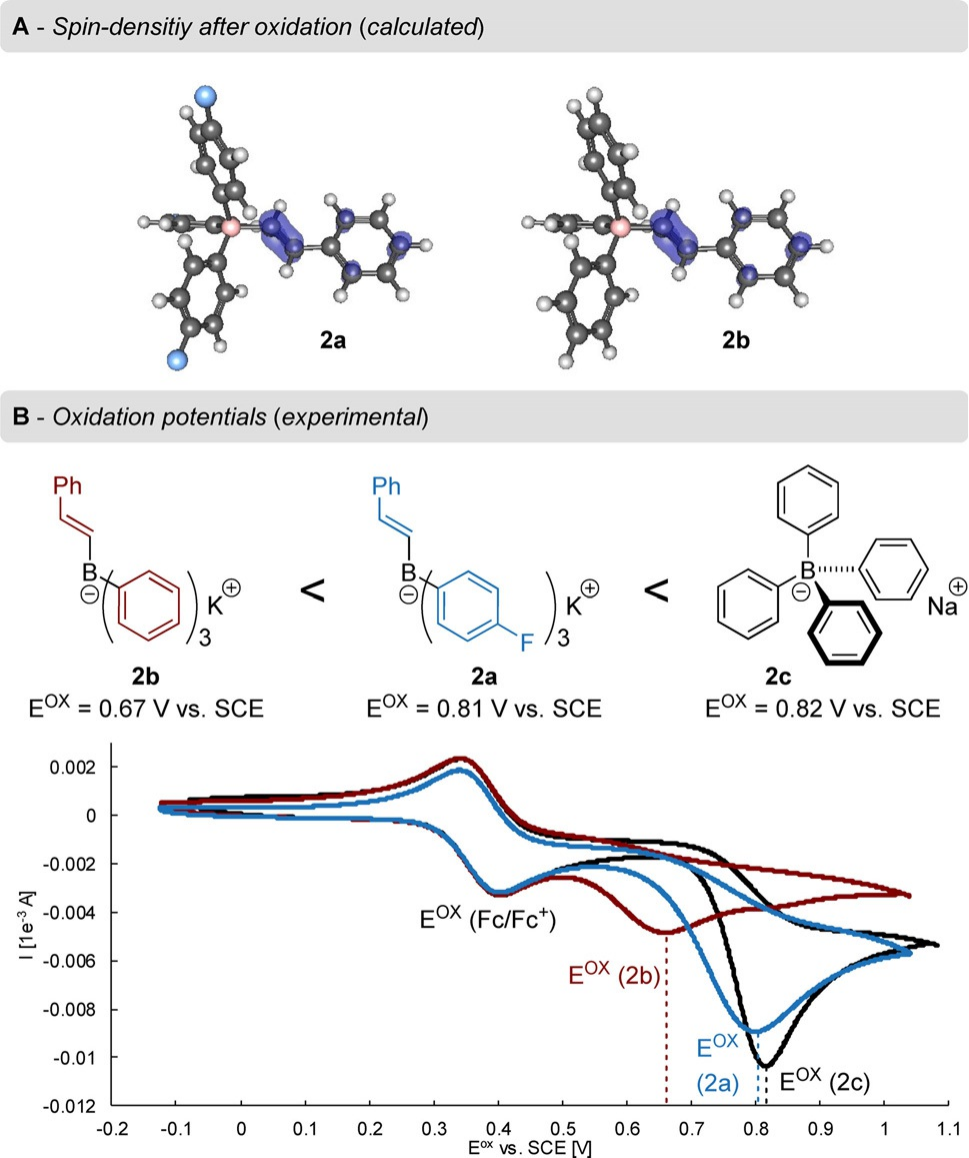
A) Spin density after oxidation of **2 a** and **2 b**. B) ATB salts **2 a**, **2 b** and tetraphenyl borate with experimental oxidation potentials and cyclic voltammetry calibrated to the reversible ferrocene oxidation (Fc/Fc^+^).

The oxidation potentials of ATB salts **2 a** and **2 b** were determined by cyclic voltammetry and compared to the value measured for commercial sodium tetraphenyl borate (Figure 1 B). With a fluoride atom present on each of the aryl groups, an *E* ^ox^ value of +0.81 V vs. SCE was measured for **2 a**, similar to the one of **2 c** (+0.82 V vs. SCE). However, in the absence of electron‐withdrawing substituents, the oxidation potential of **2 b** was decreased to +0.67 V vs. SCE. As expected, it can be concluded that alkenyl groups are easier to oxidize and that the oxidation potential varies with the electronic nature of substituents on the moieties surrounding the boron atom. From a chemoselectivity perspective, the favorable oxidation of the olefin leaves no other path for the reaction but to transfer one of the remaining aryl moieties, thereby avoiding the undesirable formation of biaryl homocoupling compounds.

**2 a** was chosen to test and optimize the reaction conditions.^14^ Inexpensive and reusable glassy carbon electrodes (GCE) proved to deliver the desired stilbene derivative **3 a** with optimal conversions in acetonitrile at 25 °C. Following the transformation by ^1^H NMR (Scheme 2) showed that the borate salt **2 a** is selectively oxidized into product **3 a**. Full conversion can be observed after 2.2 F in ^1^H NMR studies and conversion‐rate experiments of the electro‐olefination using GC revealed that an optimal yield was obtained after 2 F. Remarkably, further oxidation resulted in consumption of the reaction product. Although no biaryl byproduct was detected in ^1^H NMR, traces were found in GC. Interestingly, a third minor compound **3 ab** can be observed, which was identified as the epoxy‐stilbene derivative of **3 a**. This side reaction will be discussed later with the mechanistic considerations (Scheme 7).

**Scheme 2:**
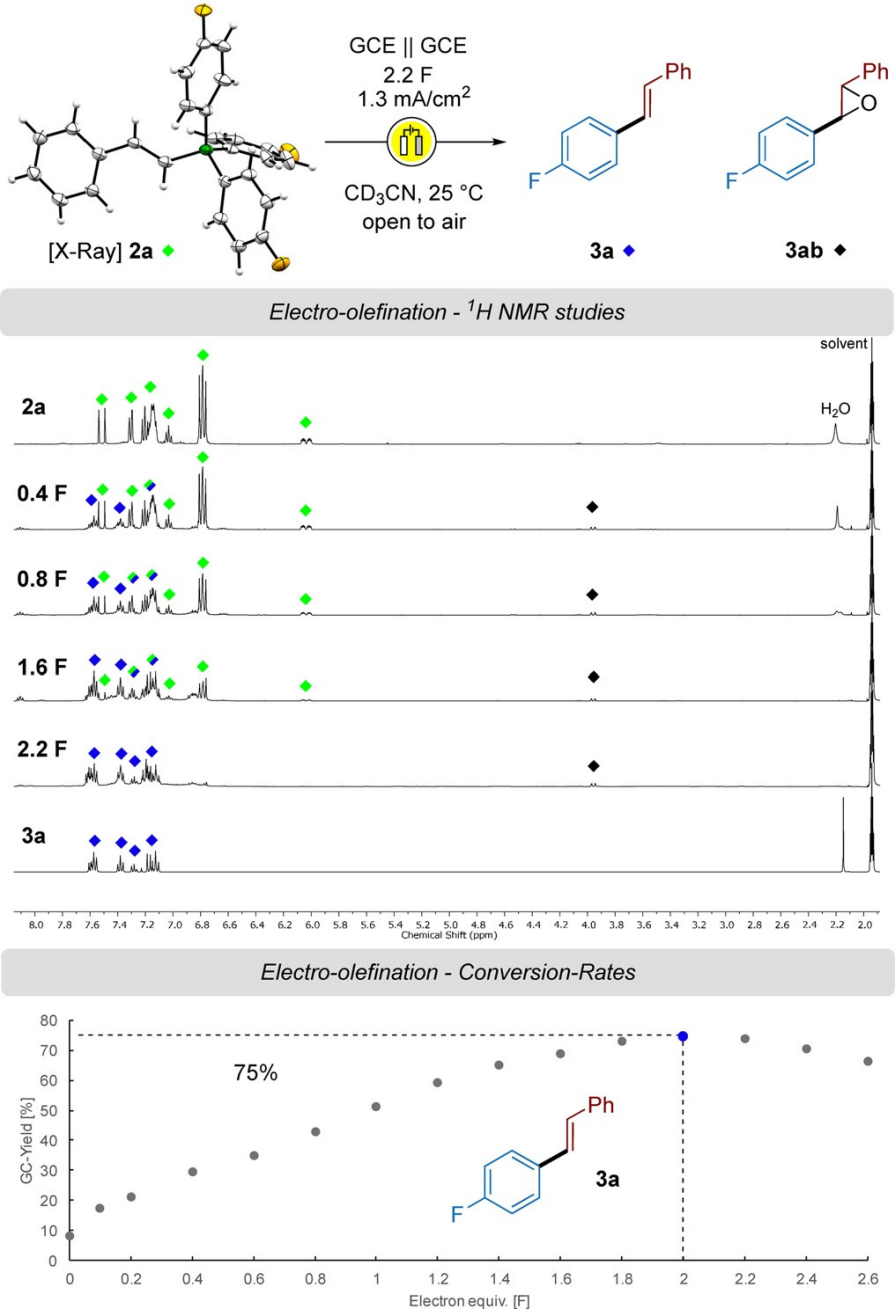
^1^H NMR studies of the transformation of **2 a** into **3 a** and **3 ab** under electrochemical oxidation and conversion‐rate (galvanostatic) experiment with *n*‐undecane as internal standard.

The synthesis of alkenyl‐borate salts can be followed by ^11^B NMR and proved quantitative when employing either Grignard reagents or—in cases of sensitive functional groups—organozinc species.^14^

Therefore, we started investigating the scope of the transformation using borate salts without prior purification. The reaction was first evaluated engaging (*E*)‐alkenyltrifluoroborates **1 a**–**g** as starting materials in this two‐pot sequence. Upon generation of the desired borate intermediates, those were treated with an aqueous solution to remove remaining inorganic salts and were subjected to electrochemical oxidation conditions after switching the solvent to acetonitrile. The results are depicted in Scheme 3. With electron‐withdrawing substituents present on the aryl moieties, (*E*)‐alkenes **3 a**–**b** were obtained in up to 69 % yield over two steps. In the case of *p*‐CN‐substituted phenyl groups, the corresponding organozinc species had to be employed, lowering the overall yield of the 2‐pot procedure (**3 c**, 29 %). This consequent decrease in yield can be attributed to the lower reactivity of organozinc derivatives in ligand exchange reactions. Electron‐donating and neutral aryl substituents furnished the desired (*E*)‐alkenes **3 d**–**e** in moderate to good yields (42 and 74 %). Varying the substitution pattern on the alkenyl moiety did not influence the course of the reaction, and **3 f**–**g** were isolated in 55 to 71 %. Heteroaryl groups were also tolerated in the electro‐olefination process, furnishing structures **3 h**–**j** in up to 68 % yield. Interestingly, trisubstituted double bonds also led to the corresponding olefinated aryl derivative **3 k** in good yield (70 %). The formation of the borate salt proved however difficult when an acrylate derivative was used. The introduction of 3‐pyridylzinc onto a trifluoroboryl acrylate and subsequent electro‐olefination only gave 25 % of product **3 l**. Notably, all derivatives were obtained with excellent (*E*/*Z*) ratios, up to 99:1

**Scheme 3:**
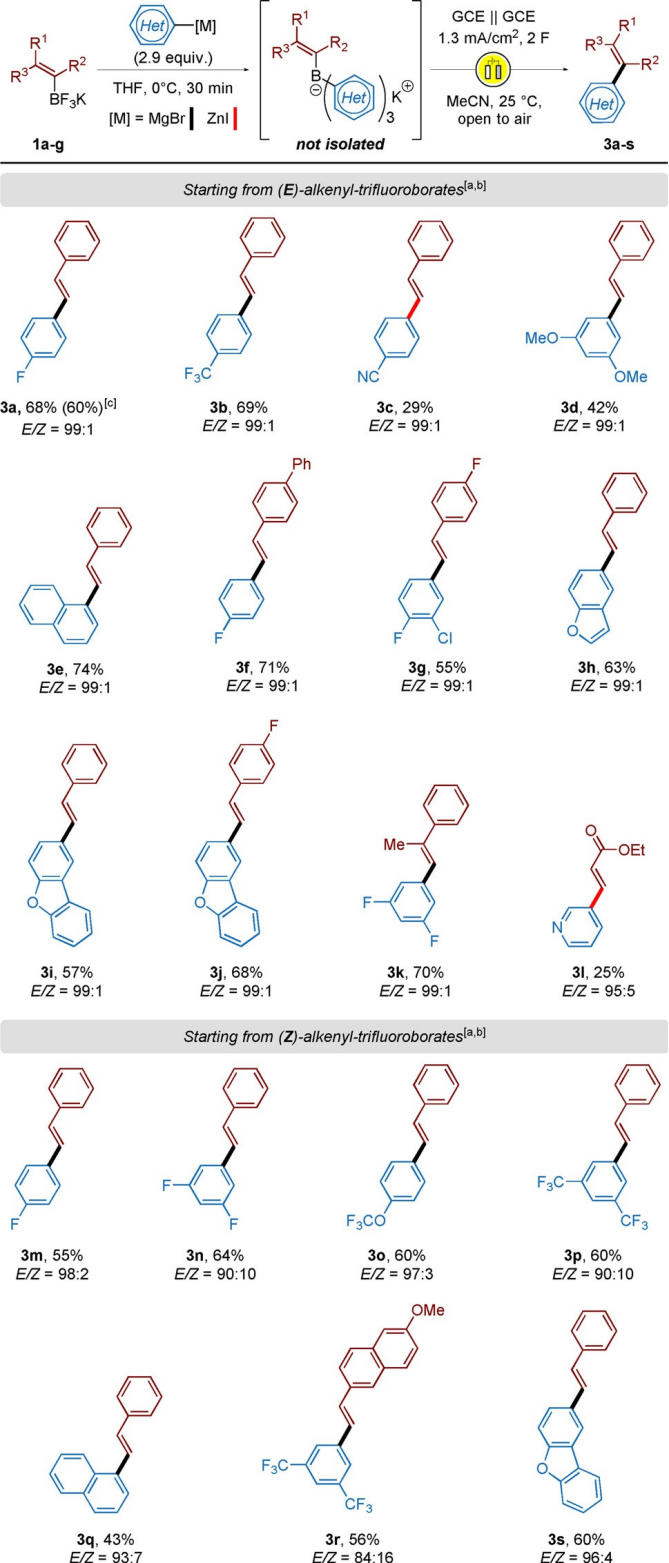
Two‐pot borate salt formation/ electro‐olefination sequence—Synthesis of acyclic alkenes. [a] Yields are stated as isolated yields over two‐steps. [b] GC‐ratios determined from crude mixtures. [c] Electrochemical oxidation in EtOH as solvent instead of MeCN at 25 °C and open to air.

*Z*‐alkenyl trifluoroborates were employed next. Following the same two‐pot protocol, the freshly generated *Z*‐alkenyl‐triaryl borates were engaged crude in the electro‐olefination under oxidative conditions. Diversely substituted aryl moieties were able to perform the coupling reaction, furnishing compounds **3 m–s** in reasonable yields (43 to 64 %). It is however interesting to notice that all derivatives were isolated as *trans*‐isomers. Given that either of the starting material (*E* or *Z*) gives the same thermodynamic *E* isomers after electro‐coupling, the strategy is stereoconvergent (Scheme 3). As it will be discussed in the mechanistic part, we assume that the oxidation of the double bond into a radical cationic species allows for the resulting bonding system to freely rotate and adopt the thermodynamically more stable configuration before abstraction of the boron‐containing moiety (Scheme 7).

Our study of the electro‐olefination was pursued with the use of α‐substituted alkenyl borates (Scheme 4). The simple acyclic isopropenyl borate salt delivered product **4 a** in 41 % yield. Cyclic alkenyl groups were then investigated in the presence of electron‐rich, ‐neutral and ‐poor aromatic systems, and gave compounds **4 b**–**f** with up to 75 % yield. Borate salts containing heteroatoms in the cycloalkenyl scaffolds such as 3,6‐dihydro‐2*H*‐pyranyl, ‐thiopyranyl and 1,2,3,6‐tetrahydropyridinylunderwent successful electro‐olefinations, delivering trisubstituted olefins **4 g**–**o** in moderate to good yields. We lastly demonstrated the reaction to be compatible in the presence of ketal functionalities (**4 p–r**, up to 86 %).

**Scheme 4:**
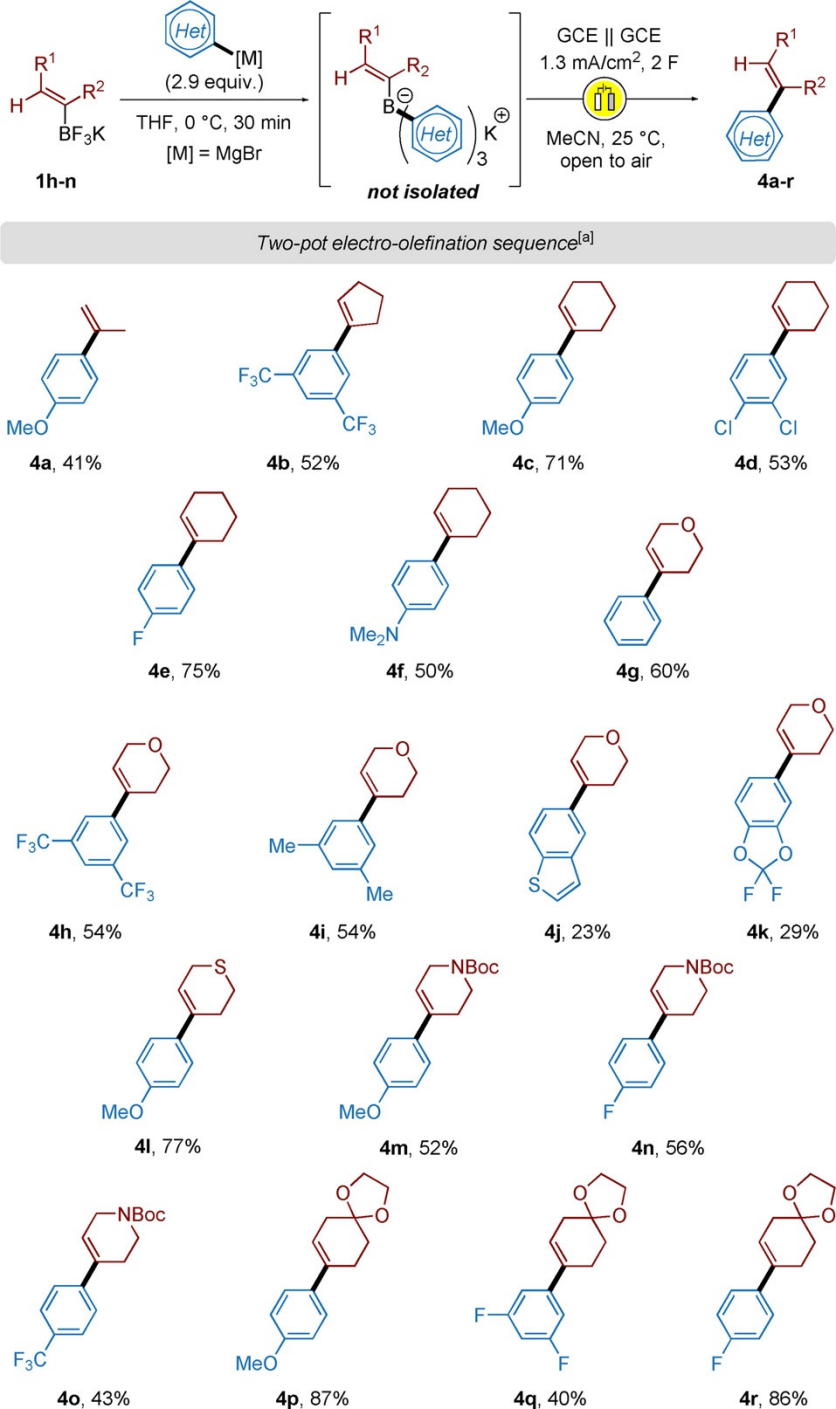
Two‐pot borate salt formation/ electro‐olefination sequence—Synthesis of cyclic alkenes. [a] Yields are stated as isolated yields over two‐steps.

Next, we applied the method to the derivatization of more challenging structures to demonstrate the synthetic potential of our ATB salts. Dehydroepiandrosterone (DHEA) was derivatized into a TBS‐protected ether and the carbonyl function transformed into the corresponding alkenyltrifluoroborate **1 o**. The addition of arylmagnesium bromide reagents to **1 o**, followed by electro‐olefination under the optimized oxidative conditions described above furnished functionalized molecules **5 a** and **5 b** in up to 70 % yield (Scheme 5 A). In addition, β‐styryltrifluoroborate **1 a** was employed as substrate for the synthesis of the natural product pinosylvin (Scheme 5 B). 3,5‐Dimethoxyphenylmagnesium bromide was introduced to perform the triple ligand exchange reaction and gave the intermediate alkenyltriaryl borate species. Subsequent electro‐olefination and demethylation with BBr_3_ furnished **5 c** in 35 % yield over three steps with perfect control of the diastereoselectivity (*E*/*Z=*99:1). Furthermore, the chemoselectivity was investigated on our benchmark salt **2 a** under distinct oxidative conditions (Scheme 5 C). As already mentioned before, the electro‐olefination occurs in a stereoconvergent manner. We selectively obtain the stilbene derivative **3 a** using (*E*)‐**2 a** or (*Z*)‐**2 a** in moderate to good yields. In contrast, typical Zweifel conditions led to a stereospecific inversion of the double bond configuration, as the reaction proceeds through two consecutive stereospecific steps (1,2‐metallate rearrangement and antiperiplanar β‐elimination). The (*Z*)‐isomer can therefore be synthesized using Zweifel conditions (Scheme 5 C) and (*Z*)‐**3 a** was isolated in 86 % yield (*E*/*Z* ratio<1:99). Noteworthy, stereodivergent Zweifel protocols have been developed. Even though the presented method might be less versatile than these contributions, our strategy avoids the use of highly toxic chemicals such as BrCN and PhSeCl.^16^

**Scheme 5:**
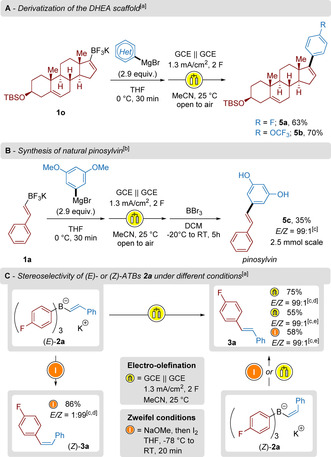
[a] Yields are stated as isolated yields over two‐steps. [b] Yield over three steps. [c] GC‐ratios determined from crude mixtures. [d] Starting from **2 a**. [e] Starting from (*Z*)‐**2 a**.

Lastly, we set out to ascertain the mechanism of this intriguing reaction, building on conversion experiments, cyclovoltammetry and theoretical considerations (Figure 1 and Scheme 2). Crossover experiments were conducted by mixing different borate salts under electrochemical conditions, confirming the absence of products resulting from intermolecular reactions and ruling out the possibility of intermolecular processes.^14^

After selective oxidation of the alkenyl moiety, a rearrangement takes place. To study the nature of this rearrangement, we synthesized borate salts containing more than a single styryl group (**6 a**–**b**, Scheme 6), employing styryl‐Grignard reagents as (*E*/*Z*)‐mixtures, and submitted them to our electrocoupling conditions. As a reference, the desired compound **3 a** was obtained as the sole compound from **2 a**. With a salt bearing two styryl groups (**6 a**), a product ratio of 73:27 of **3 a** and the diene **7** was obtained (*E*/*Z=*85:15). This result points out that the transfer of a vinyl group is not preferred over the transfer of an aryl group, and therefore indicates that the rearrangement is more likely to go through a σ‐bond breaking process rather than a π‐addition, as for the latter an unfavorable dearomatization has to occur. Example **6 b** (possessing three styryl moieties) further supports this hypothesis, as **7** was obtained in 54 % and **3 a** in 46 % GC‐ratio. The non‐statistical distribution of products **3 a** and **7** in both experiments also indicates that the aryl moiety is—in such cases—a better transferable ligand than the styryl group.

**Scheme 6:**
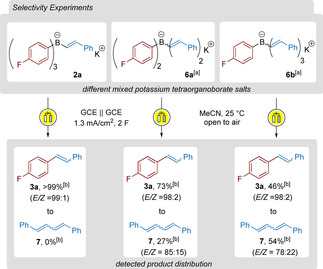
Electrocoupling of different mixed potassium tetraorganoborate salts. [a] In situ generated following general procedure **D** 14 as follows for **6 a**: 0.5 mmol **1 p** and 1.0 mmol styrylmagnesium bromide. For **6 b**: 0.5 mmol potassium trifluoro(4‐fluorophenyl)borate and 1.5 mmol styrylmagnesium bromide. [b] Product distribution ratios are determined by GC analysis on crude mixtures without isolation. Homocoupled biaryls are omitted and not included in the GC‐ratios for more clarity.

In summary, the alkenyl moiety is more prone to oxidation than the aryl groups (as concluded from quantum‐chemical calculations and selectivity experiments, see Figure 1 and Scheme 6) and leads to an intermediate alkyl radical cationic species [**A**] (Scheme 7). We then propose that further intramolecular σ‐addition of one of the aryl moieties undergoes a rearrangement^17^ towards intermediate [**B**] in which the C−C alkyl radical bond can freely rotate and lead to the thermodynamically favored *trans* product (*E*)‐**3 a**. Oxygen probably interacts with the reaction intermediates under formation of structure [**C**], as **3 ab** was observed in traces under air and isolated in 37 % yield when the reaction was carried out under oxygen atmosphere. It is however important to note that product **3 ab** does not come from the oxidation of product **3 a** under electrochemical conditions, as confirmed by control experiments, indicating a radical pathway.^14^ Based on cyclovoltametry (Figure 1), galvanostatic experiments (Scheme 2) and our findings in the previous work on biaryl electro‐coupling,^7^ we assume that no second oxidation has to occur during the formation of the desired product **3 a**.

**Scheme 7:**
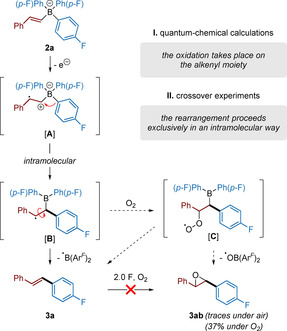
Proposed mechanism for the electro‐olefination of ATB **2 a**.

In conclusion, we have developed a new conceptual approach to alkene derivatives through electro‐olefination. A simple strategy was assembled for the synthesis of alkenylborate salts (ATBs) through ligand exchanges on potassium trifluoroborates. No purification of these salts was required for the sequence to be pursued and deliver the expected coupling compounds in moderate to good yields under electrochemical oxidation. Such method represents an original and stereoconvergent alternative to the formation of functionalized olefins, opening new ways of thinking about C−C bond disconnections.

## Conflict of interest

The authors declare no conflict of interest.

## Supporting information

As a service to our authors and readers, this journal provides supporting information supplied by the authors. Such materials are peer reviewed and may be re‐organized for online delivery, but are not copy‐edited or typeset. Technical support issues arising from supporting information (other than missing files) should be addressed to the authors.

Supplementary
