# Supplementary material for: Electro‐Olefination—A Catalyst Free Stereoconvergent Strategy for the Functionalization of Alkenes
Source: Chemistry. 2020 Jun 25;26(38):8382–7. doi: 10.1002/chem.202001394 (PMC7383514; doi:10.1002/chem.202001394)
Supplement: Supplementary file 1 — Supplementary [file CHEM-26-8382-s001.pdf]

Chemistry–A European Journal

Supporting Information

Electro-Olefination—A Catalyst Free Stereoconvergent Strategy for the Functionalization of Alkenes

Andreas N. Baumann+, Arif Music+, Jonas Dechent, Nicolas Müller, Thomas C. Jagau, and Dorian Didier*[a]

Electro-Olefination - a Catalyst Free Stereoconvergent Strategy for the Functionalization of Alkenes

*Andreas N. Baumann, Arif Music, Jonas Dechent, Nicolas Müller, Thomas C. Jagau and Dorian Didier**

Department Chemie,

Ludwig-Maximilians-Universität

Butenandtstraße 5-13, D-81377 Munich

dorian.didier@cup.uni-muenchen.de

Supporting Information

1. General Considerations **2**

2. Experimental Procedures and Data **4**

3. NMR Spectra **39**

4. Cyclic Voltammetry **87**

5. Theoretical Calculations **89**

6. Single Crystal X-Ray Diffraction **93**

1. General Considerations

Commercially available starting materials such as bromides and potassium alkenyl trifluoroborate salts (**1a, 1h, 1j**) were used without further purification unless otherwise stated. All reactions were carried out under N_2 atmosphere in flame-dried glassware. Syringes, which were used to transfer anhydrous solvents or reagents, were purged with nitrogen prior to use.

THF was refluxed and distilled from sodium benzophenone ketyl under nitrogen. Et_2O was predried over $CaCl_2$ and passed through activated Al_2O_3 (the solvent purification system SPS-400-2 from Innovative Technologies Inc.). MeCN was purchased in HPLC gradient grade (>=99.9%) from Fisher Scientific.

Chromatography purifications were performed using silica gel (SiO_2, 0.040-0.063 mm, 230-400 mesh ASTM) from Merck. The spots were visualized under UV (254 nm) or by staining the TLC plate with either $KMnO_4$ solution (K_2CO_3, 10 g – $KMnO_4$, 1.5 g – H_2O, 150 ml – NaOH 10% in H_2O, 1.25 ml) or *p*-anisaldehyde solution (conc. H_2SO_4, 10 ml – EtOH, 200 ml – AcOH, 3 ml – *p*-anisaldehyde, 4 ml). Yields refer to isolated yields of compounds estimated to be >95% pure as determined by ^{1}H NMR and GC-analysis. The ^{13}C and ^{1}H NMR spectra were recorded on VARIAN Mercury 200, BRUKER ARX 300, VARIAN VXR 400 S and BRUKER AMX 600 instruments. Chemical shifts are reported as δ values in ppm relative to the residual solvent peak (^{1}H-NMR, ^{13}C-NMR) in deuterated chloroform ($CDCl_3$: δ 7.26 ppm for ^{1}H-NMR and δ 77.16 ppm for ^{13}C-NMR), deuterated acetonitrile (CD_3CN: δ 1.94 ppm for ^{1}H-NMR and δ 118.69 and 1.39 ppm for ^{13}C-NMR). Abbreviations for signal coupling are as follows: s (singlet), d (doublet), t (triplet), q (quartet), quint (quintet), m (multiplet) and br (broad). Reaction endpoints were determined by GC monitoring of the reactions with *n*-undecane as an internal standard. Gas chromatography was performed with machines of Agilent Technologies 7890, using a column of type HP 5 (Agilent 5% phenylmethylpolysiloxane; length: 15 m; diameter: 0.25 mm; film thickness: 0.25 µm) or Hewlett-Packard 6890 or 5890 series II, using a column of type HP 5 (Hewlett-Packard, 5% phenylmethylpolysiloxane; length: 15 m; diameter: 0.25 mm; film thickness: 0.25 µm). High resolution mass spectra (HRMS) and low resolution mass spectra (LRMS) were recorded on Finnigan MAT 95Q, Finnigan MAT 90 instrument or JEOL JMS-700. Infrared spectra were recorded on a Perkin 281 IR spectrometer and samples were measured neat (ATR, Smiths Detection DuraSample IR II Diamond ATR). The absorption bands were reported in wave numbers (cm^{-1}) and abbreviations for intensity are as follows: vs (very strong; maximum intensity), s (strong; above 75% of max. intensity), m (medium; from 50% to 75% of max. intensity), w (weak; below 50% of max. intensity) and br (broad). Melting points were determined on a Büchi B-540 apparatus and are uncorrected.

Electrochemical Oxidations on scales smaller than 1.0 mmol were performed on the IKA ElectraSyn 2.0. All used electrodes were purchased from IKA, except the RVC (reticulated vitreous carbon) electrodes

which were obtained from Goodfellow (Carbon – Vitreous – 3000C Foam, Thickness: 6.35 mm, Bulk density: 0.05 g/cm^3, Porosity: 96.5%, Pores/cm: 24). Electrochemical Oxidations on a scale greater than 1.0 mmol were performed on an Atlas 0931 Potentiostat – Galvanostat using a two-electrode undivided cell setup.

n-BuLi was purchased as a solution in cyclohexane/hexanes mixtures from Rockwood Lithium GmbH. The concentration of organometallic reagent from commercially purchased and synthesized reagents was determined either by titration of isopropyl alcohol using the indicator 1,10-phenanthroline or by titration of *N*-benzylbenzamide in THF for organolithium reagents. Aryl-Grignard reagents and Arylzinc reagents were titrated using iodine in THF at room temperature.

2. Experimental Procedures and Data

2.1 General Procedure A: Synthesis of Arylgrignard reagents

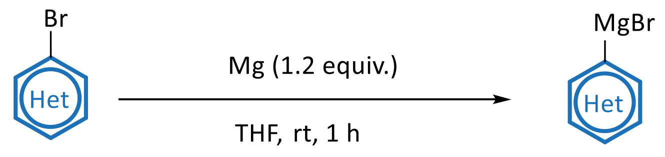

A-Br **A1-18**

A Schlenk flask was charged with magnesium turnings (720 mg, 30 mmol, 1.2 equiv.) and dried *in vacuo* using a heat gun (600 °C, 2 x 5 min). After addition of THF (2.0 mL) and 1,2-dibromoethane (2 drops), the mixture was heated to boil with a heat gun to activate the magnesium. The aryl bromide **A-Br** (25 mmol, 1.0 equiv.) was dissolved in THF (23.0 mL for approximately 1 M solution or 48.0 ml for 0.5 M solution) and added to the activated magnesium suspension dropwise. After completion of the addition, the mixture was stirred for one hour at room temperature to yield a THF-solution of the arylmagnesium reagents.

2.2 General Procedure B: Synthesis of Arylzinc reagents

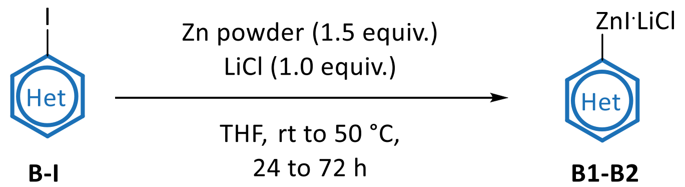

B-I **B1-B2**

According to a previously reported procedure,[1] a Schlenk flask was charged with lithium chloride (212 mg, 5 mmol, 1.0 equiv.) and dried *in vacuo* using a heat gun (500 °C, 2 x 5 min). Zinc powder (490 mg, 7.5 mmol, 1.5 equiv.) was added and the flask was dried again *in vacuo* (350 °C, 2 x 5 min). After addition of THF (5.0 mL), 1,2-dibromoethane (2 drops) and TMS-Cl (5 drops), the mixture was heated to boil with a heat gun to activate the zinc. The aryl iodide **B-I** (5 mmol, 1.0 equiv.) was added neat to the activated zinc suspension at room temperature and the reaction was stirred at 50 °C until complete consumption of the aryl iodide **B-I** was observed by GC analysis.

[1] Krasovskiy, A.; Malakhov, V.; Gavryushin, A.; Knochel, P. *Angew. Chem. Int. Ed.* **2006**, *118*, 6186.

2.3 General Procedure C: Preparation of potassium alkenyl trifluoroborate salts starting from alkenyl boronic esters and acids

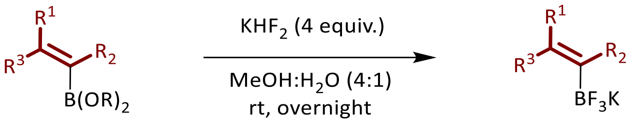

1

Adapted from a previously reported procedure,[2] 5.0 mmol (1.0 equiv.) of commercially available alkenyl boronic pinacol esters and boronic acids were dissolved in 15 mL of a 4:1 (v/v) mixture of MeOH and H_2O. The mixture was cooled to 0 °C and KHF_2 (20 mmol, 4.0 equiv.) was added neat. The mixture was vigorously stirred at room temperature overnight and then concentrated under reduced pressure. The remaining solids were extracted with boiling acetone (2 x 50 mL) and twice with acetone at room temperature (2 x 50 mL). The acetone was removed under reduced pressure and the remaining solid was dissolved in a minimum amount of boiling acetone, before being treated with diethyl ether, which resulted in precipitation of a colourless solid. The solids were filtered, washed with diethyl ether and dried in vacuo to yield potassium alkenyl trifluoroborate salts **1**.

Note: Literature known potassium alkenyl trifluoroborate salts were synthesized according to the same procedure **C** and used without further purification:

- **1b** analytical data in accordance with literature.[3]

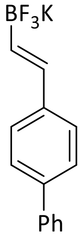

- **1c** analytical data in accordance with literature.[4]

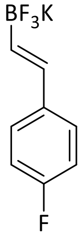

[2] Santos-Filho, E. F.; Sousa, J. C.; Bezerra, N. M. M.; Menezes, P. H. *Tetrahedron Letters* **2011**, *52*, 5288-5291.
[3] M. Presset; D. Oehlrich; F. Rombouts; G. A. Molander, *J. Org. Chem.* **2013**, *78*, 12837.
[4] B. Gopula; C.-W. Chiang; W.-Z. Lee; T.-S. Kuo; P.-Y. Wu; J. P. Henschke; H.-L. Wu, *Org. Lett.* **2014**, 16, 632.

- **1d** analytical data in accordance with literature.[5]

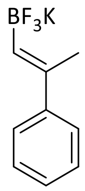

- **1e** analytical data in accordance with literature.[6]

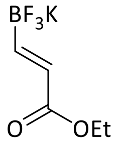

- **1f** analytical data in accordance with literature.[7]

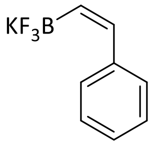

- **1m** analytical data in accordance with literature.[8]

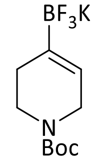

---

[5] J. J. Molloy; J. B. Metternich; C. G. Daniliuc; A. J. B. Watson; R. Gilmour, *Angew. Chem. Int. Ed.* **2018**, *57*, 3168.
[6] C. Feng; H. Wang; L. Xu; P. Li, *Org. Biomol. Chem.* **2015**, *13*, 7136.
[7] G. A. Molander; N. M. Ellis, *J. Org. Chem.* **2008**, *73*, 6841.
[8] M. Presset; D. Oehlrich; F. Rombouts; G. A. Molander, *J. Org. Chem.* **2013**, *78*, 12837.

2.4 General Procedure D: Two-pot Procedure for the synthesis of functionalized alkenes starting from potassium alkenyl trifluoroborate salts

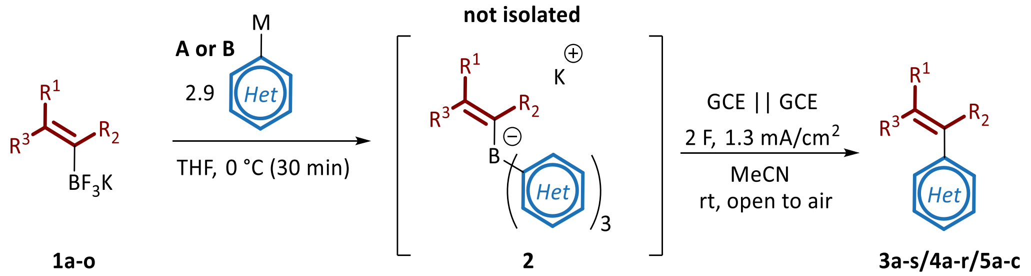

A 25 mL Schlenk flask was charged with the corresponding potassium trifluoroborate salt **1a-o** (0.4 mmol, 1.0 equiv.) and 2 mL of THF were added. The mixture was cooled to 0 °C and the aryl-Grignard reagent **A** (1.16 mmol, 2.9 equiv.) was added dropwise over 30 minutes *via* syringe pump. After addition, the reaction mixture was allowed to stir for further 10 min at 0 °C and was then quenched with 5 mL of H_2O and extracted with EtOAc (3 x 40 mL). If no phase separation was observed, 5 mL of aqueous saturated K_2CO_3 solution was added. The combined organic phases were filtered and concentrated under reduced pressure (no higher temperature than 40 °C). The crude tetraorganoborate **2** was then dissolved in 8 mL of HPLC grade MeCN and transferred into a 10 mL IKA glass vial. The reaction was started using the IKA ElectraSyn 2.0 with GCE (glassy carbon electrodes) as working and counter electrode (5 mA, 2.0 F, 1.3 mA/cm^2, 700 rpm stirring). The crude was then treated with water and extracted with diethyl ether (3 x 15 mL). The combined organic phases were dried over magnesium sulfate, filtered, concentrated under reduced pressure and purified by flash-column chromatography on silica gel with the appropriate solvent mixture to obtain pure **3a-b,d-k,m-s/4a-r/5a-c**.

---

a) Adaptation for the use of arylzinc reagents

After addition of the arylzinc **B** instead of the aryl-Grignard reagent *via* syringe pump, the reaction was heated to 40 °C for 16 hours to ensure full conversion of the potassium trifluoroborate salt into the desired salt **2**. General Procedure **D** was then followed to give products **3c** and **3l.**

2.5 General Procedure E: Procedure for the isolation of functionalized ATB salts starting from potassium alkenyl trifluoroborate salts

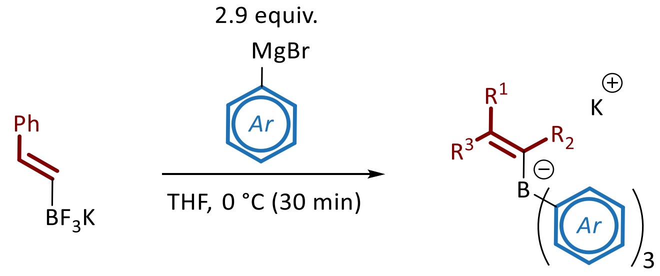

1a **2a-b**

A 50 mL Schlenk flask was charged with (*E*)-trifluoro(styryl)borate **1a** (3.0 mmol, 1.0 equiv.) and 9 mL of THF were added. The mixture was cooled to 0 °C and the corresponding aryl-Grignard reagent **A** (9.0 mmol, 3.0 equiv.) was added dropwise over 30 minutes *via* syringe pump. After addition, the reaction mixture was allowed to stir for further 10 min at 0 °C and was then quenched with 5 mL of H_2O and extracted with EtOAc (3 x 40 mL). If no phase separation was observed, 5 mL of aqueous saturated K_2CO_3 solution was added. The combined organic phases were filtered and concentrated under reduced pressure (no higher temperature than 40 °C). The resulting oil was then layered with hexane (20 mL) and sonicated at 0 °C for 10 minutes. The hexane was decanted and the process repeated two more times, until a white solid was obtained. (Note: ATB salts are highly soluble in EtOAc and therefore solidification can be challenging.) The solids were then again sonicated in hexane, the fine white powder was then filtered and washed with hexanes (2 x 10 mL) and dried in vacuo to yield ATB salts **2a-b**.

2.6 Optimization of the Electrochemical coupling

Conversion rates into (*E*)-1-fluoro-4-styrylbenzene (**3a**) were assessed by hydrolysis and GC analysis with *n*-undecane as an internal standard.

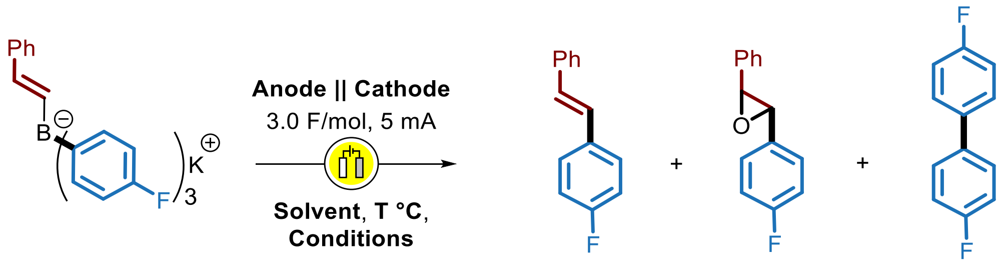

2a **3a** **3ab** **3ac**

Table 1: Screening of different electrode material, solvents and conditions.

Anode || Cathode	Solvent	Conditions	T (°C)	conv. (%) 3a:3ab:3ac
Graphite || Graphite	MeCN	Open to air no Electrolyte	25	77:5:2
RVC || RVC	MeCN	Open to air no Electrolyte	25	80:10:3
**Glassy carbon || Glassy carbon**	**MeCN**	**Open to air no Electrolyte**	**25**	**82:5:5 (isolated 75%)**
Glassy carbon || Glassy carbon	MeCN	N_2-atmosphere no Electrolyte	25	65:2:3
Glassy carbon || Glassy carbon	MeCN	O_2-atmosphere no Electrolyte	25	30:37:4
Glassy carbon || Glassy carbon	MeCN	Open to air with $LiClO_4$ [0.1 M]	25	75:4:3
Glassy carbon || Glassy carbon	EtOH	Open to air no Electrolyte	25	78:5:5
RVC || RVC	EtOH	Open to air no Electrolyte	25	73:9:1

As seen in Table 1, the oxidation process can be performed with different carbon electrode setups, resulting in good conversion and selectivity ratios. In addition, the oxidation process can also be performed in environmentally friendly solvents such as ethanol with only marginal conversion loss.

2.7 Formation of ATB salt 2a by ^{11}B NMR

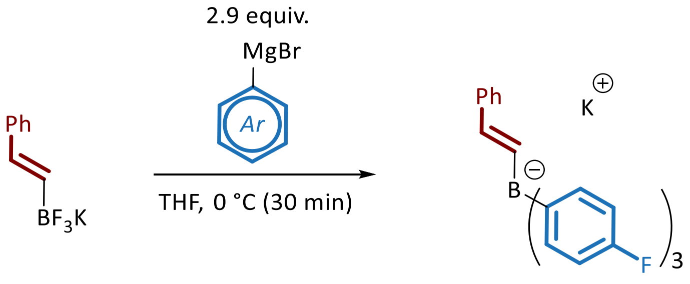

1a **2a**

Figure 1: 11B NMR analysis of the ATB salt formation to yield 2a.

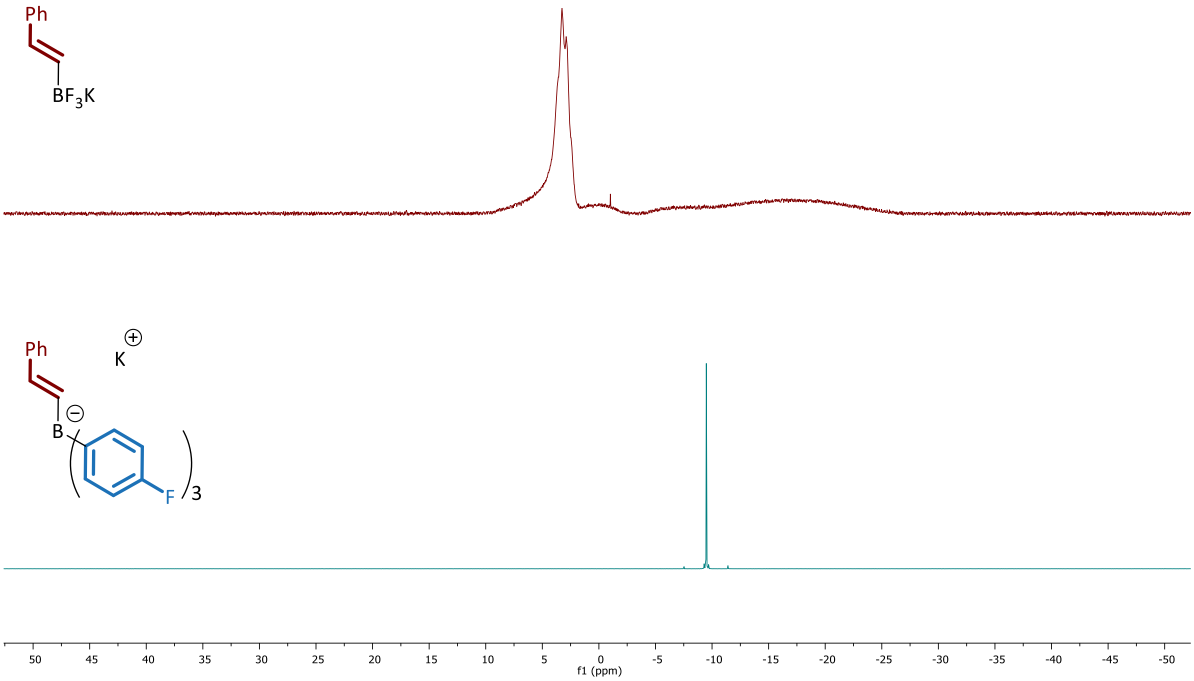

As depicted in Figure 1, a smooth transformation of the starting (*E*)-trifluoro(styryl)borate into the desired ATB salt **2a** was observed in the crude ^{11}B NMR, which was measured as a 1:1 THF:CD_3CN mixture following general procedure **E**.

2.8 Experimental Data

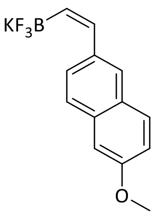

Potassium (*Z*)-trifluoro(2-(6-methoxynaphthalen-2-yl)vinyl)borate (1g)

Using (*Z*)-2-(2-(6-methoxynaphthalen-2-yl)vinyl)-4,4,5,5-tetramethyl-1,3,2-dioxaborolane according to general procedure **C**, provided **1g** (4.09 mmol, 1.186 g, 48%) as brownish solid.

^{1}H NMR (400 MHz, CD_3CN) δ 7.97 (dd, *J* = 8.6, 1.7 Hz, 1H), 7.81 (s, 1H), 7.66 (dd, *J* = 18.2, 8.8 Hz, 2H), 7.19 (d, *J* = 2.6 Hz, 1H), 7.07 (dd, *J* = 8.9, 2.6 Hz, 1H), 6.73 (d, *J* = 15.0 Hz, 1H), 5.70 (dq, *J* = 15.2, 6.6 Hz, 1H), 3.88 ppm (s, 3H). **^{13}C NMR** (101 MHz, CD_3CN) δ 158.1, 137.9, 136.7, 134.2, 130.2, 129.8, 128.8, 127.7, 126.5, 119.0, 106.6, 55.9 ppm. **^{11}B NMR** (128 MHz, CD_3CN) δ 2.43 (q, *J* = 54.7 Hz). **HRMS** (ESI-Quadrupole): m/z: calcd for $C_{13}H_{11}OBF_3KNa^+$ $[M+Na]^+$: 313.0390; found: 313.0385. **IR** (Diamond-ATR, neat) $\tilde{\nu}_{max}$ (cm^{-1}): 1686 (m), 1680 (m), 1623 (m), 1605 (m), 1482 (m), 1390 (m), 1292 (w), 1268 (m), 1258 (m), 1210 (m), 1196 (m), 1186 (m), 1164 (s), 1118 (m), 1108 (m), 1090 (s), 1084 (s), 1067 (s), 1060 (s), 1050 (s), 1030 (vs), 990 (s), 982 (s), 966 (s), 958 (s), 950 (s), 934 (s), 881 (m), 860 (s), 854 (s), 834 (m), 804 (s), 780 (m), 768 (m), 758 (m). **Mp** (°C) = 160-182 (decomposition).

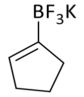

Potassium cyclopent-1-en-1-yltrifluoroborate (1i)

Using 2-(cyclopent-1-en-1-yl)-4,4,5,5-tetramethyl-1,3,2-dioxaborolane according to general procedure **C**, provided **1i** (3.76 mmol, 658 mg, 84%) as colourless solid.

^{1}H NMR (400 MHz, CD_3CN) δ 5.48 (s, 1H), 2.25-2.19 (m, 2H), 1.94 (p, *J* = 2.5 Hz, 2H), 1.69 ppm (p, *J* = 7.5 Hz, 2H). **^{13}C NMR** (101 MHz, CD_3CN) δ 128.8, 36.2, 34.5, 24.8 ppm. **^{11}B NMR** (128 MHz, CD_3CN) δ 2.71 (q, *J* = 57.1 Hz). **HRMS** (ESI-Quadrupole): m/z: calcd for $C_5H_7BF_3^-$ $[M-K]^-$: 135.0593; found: 135.0597. **IR** (Diamond-ATR, neat) $\tilde{\nu}_{max}$ (cm^{-1}): 2948 (w), 2843 (w), 1620 (w), 1291 (w), 1224 (w), 1152 (m), 1038 (m), 1021 (m), 980 (m), 949 (s), 916 (vs), 885 (s), 840 (m), 806 (m). Mp (°C) = 200-210 (decomposition).

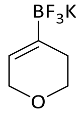

Potassium (3,6-dihydro-2H-pyran-4-yl)trifluoroborate (1k)

Using 2-(3,6-dihydro-2H-pyran-4-yl)-4,4,5,5-tetramethyl-1,3,2-dioxaborolane according to general procedure **C**, provided **1k** (6.84 mmol, 1.30 g, 68%) as colourless solid.

^{1}H NMR (400 MHz, CD_3CN) δ 5.55 (s, 1H), 3.96 (qd, *J* = 2.6, 1.3 Hz, 2H), 3.61 (t, *J* = 5.5 Hz, 2H), 2.00 ppm (dq, *J* = 5.6, 2.8 Hz, 2H). **^{13}C NMR** (101 MHz, CD_3CN) δ 123.8, 66.6, 65.7, 27.8 ppm. **^{11}B NMR** (128 MHz, CD_3CN) δ 2.49 (q, *J* = 56.2, 55.3 Hz). **HRMS** (ESI-Quadrupole): m/z: calcd for $C_5H_7OBF_3^-$ $[M-K]^-$: 151.0542; found: 151.0547. **IR** (Diamond-ATR, neat) $\tilde{\nu}_{max}$ (cm^{-1}): 1239 (m), 1211 (m), 1176 (s), 1114 (m), 1066 (m), 1035 (s), 1005 (s), 989 (s), 965 (s), 939 (vs), 919 (vs), 841 (s), 813 (m), 760 (m). Mp (°C) = 190-192.

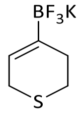

Potassium (3,6-dihydro-2H-thiopyran-4-yl)trifluoroborate (1l)

Using 2-(3,6-dihydro-2H-thiopyran-4-yl)-4,4,5,5-tetramethyl-1,3,2-dioxaborolane according to general procedure **C**, provided **1l** (3.49 mmol, 718 mg, 84%) as colourless solid.

^{1}H NMR (400 MHz, CD_3CN) δ 5.79 (s, 1H), 3.06-2.97 (m, 2H), 2.60 (t, *J* = 5.8 Hz, 2H), 2.22-2.17 ppm (m, 2H). **^{13}C NMR** (101 MHz, CD_3CN) δ 120.7, 27.3, 26.1, 25.9 ppm. **HRMS** (ESI-Quadrupole): m/z: calcd for $C_5H_7SBF_3^-$ $[M-K]^-$: 167.0314; found: 167.0318.

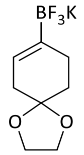

Potassium trifluoro(1,4-dioxaspiro[4.5]dec-7-en-8-yl)borate (1n)

Using 4,4,5,5-tetramethyl-2-(1,4-dioxaspiro[4.5]dec-7-en-8-yl)-1,3,2-dioxaborolane according to general procedure **C**, provided **1n** (3.43 mmol, 843 mg, 59%) as colourless solid.

^{1}H NMR (400 MHz, CD_3CN) δ 5.47 (s, 1H), 3.88 (s, 4H), 2.14-2.07 (m, 4H), 1.58 ppm (t, *J* = 6.4 Hz, 2H). **^{13}C NMR** (101 MHz, CD_3CN) δ 121.9, 109.6, 64.6, 37.0, 32.2, 27.1 ppm. **^{11}B NMR** (128 MHz, CD_3CN) δ 2.61 (q, *J* = 56.4 Hz). **HRMS** (ESI-Quadrupole): m/z: calcd for $C_8H_{11}O_2BF_3^-$ $[M-K]^-$: 207.0804; found: 207.0809. **IR** (Diamond-ATR, neat) $\tilde{\nu}_{max}$ (cm^{-1}): 1693 (w), 1209 (w), 1201 (w), 1170 (w), 1150 (m),

1128 (m), 1108 (m), 1058 (m), 1048 (m), 1010 (s), 941 (vs), 894 (s), 857 (m), 816 (w), 812 (w), 789 (m). Mp (°C) = 185-188.

Experimental Procedures for the Synthesis of 1o

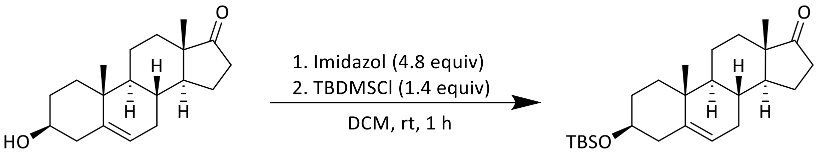

3-TBS-5-DHEA was prepared according to Pérez *et al.*[9] To a suspension of 5-dehydroepiandrosterone (15.0 mmol, 4.33 g) in DCM (50 mL) was added imidazole (72.0 mmol, 4.90 g). When a clear solution had formed, TBSCl (21.0 mmol, 3.16 g, 1.4 equiv.) was added. The mixture was stirred at room temperature for 2 h and then concentrated in vacuo. The crude product was dissolved in DCM (50 mL) and washed with 1 M HCl (2 x 50 mL) and water (2 x 50 mL). The organic phase was dried over $MgSO_4$ and evaporated in vacuo. The white solid was dried under high vacuum at 60 °C to obtain the pure product in 90% yield (13.5 mmol, 5.4 g), which was used without further purification for the following step.

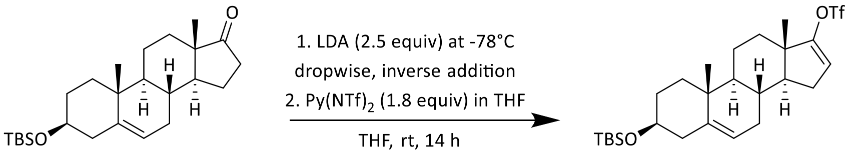

3-TBS-5-DHEA triflate was prepared according to procedures from Lopez *et al.*[10] A solution of LDA was freshly prepared by dropwise addition of *n*-butyllithium (11.5 mmol, 4.64 mL, 2.47 M in hexane, 2.3 equiv.) to a solution of DIPA (12.5 mmol, 1.26 g, 1.75 mL, 2.5 equiv.) in THF at −78 °C. To this solution, a suspension of 3-TBS-5-DHEA (5.00 mmol, 2.01 g) in THF (25 mL) was added dropwise at −78 °C. After stirring for 1 h, a solution of $Py(NTf_2)$ (9.00 mmol, 3.22 g, 1.8 equiv.) in THF (12.5 mL) was added dropwise at −78 °C. The reaction mixture was stirred at room temperature overnight and then filtered over silica gel and washed with DCM (2 x 50 mL). The filtrate was concentrated in vacuo. The resulting white solid was purified via flash column chromatography (hexanes/EtOAc 98:2) to give the product in 80% yield (4 mmol, 2.13 g), which was directly engaged in the next step.

---

[9] A. Pérez Encabo, J. A. Turiel Hernandez, F. J. Gallo Nieto, A. Lorente Bonde-Larsen, C. M. Sandoval Rodríguez C07J 41/00, **2013.**

[10] B. Lopez-Perez, M. A. Maestro, A. Mourino, *Chem. Commun.* **2017**, *53*, 8144.

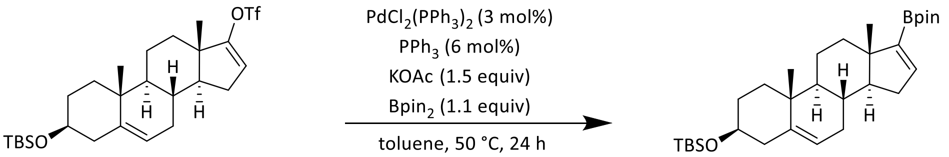

1o-SM

3-TBS-5-DHEA alkenylboronic acid pinacol ester was prepared according to procedures from Tagaki *et al.*[11] A dried flask was charged with 3-TBS-5-DHEA alkenyl triflate (1.24 mmol, 661 mg), $PdCl_2(PPh_3)_2$ (0.037 mmol, 26 mg, 3 mol%), PPh_3 (0.074 mmol, 19 mg, 6 mol%), B_2pin_2 (1.36 mmol, 345 mg, 1.1 equiv.) and toluene (7.5 mL). After adding KOAc (1.85 mmol, 182 mg, 1.5 equiv.), the flask was flushed with nitrogen, sealed and stirred at 50 °C for 24 h. The mixture was filtered over $MgSO_4$, washed with THF (20 mL) and concentrated in vacuo. The resulting white solid was purified via flash column chromatography (hexanes/EtOAc 95:5) to give the product in **1o-SM** 86% yield (1 mmol, 512 mg).

R_f = 0.60 (hexane/EtOAc 95:5, UV, $KMnO_4$). **^{1}H NMR** (400 MHz, $CDCl_3$) δ 6.50 (dd, *J* = 3.1, 1.6 Hz, 1H), 5.35 – 5.31 (m, 1H), 3.48 (tt, *J* = 10.9, 4.7 Hz, 1H), 2.34 – 2.22 (m, 1H), 2.22 – 2.05 (m, 3H), 2.05 – 1.89 (m, 2H), 1.81 (dt, *J* = 13.4, 3.6 Hz, 1H), 1.76 – 1.45 (m, 8H), 1.41 – 1.27 (m, 3H), 1.25 (s, 12H), 1.03 (s, 3H), 0.89 (s, 9H), 0.06 ppm (s, 6H). **^{13}C NMR** (101 MHz, $CDCl_3$) δ 146.1, 142.1, 121.2, 82.8, 72.8, 57.0, 53.6, 51.0, 47.8, 43.0, 37.5, 37.0, 35.9, 33.8, 32.2, 32.2, 30.8, 26.1, 25.0, 24.9, 21.0, 19.5, 18.5, 16.8, 1.2, −4.5 ppm. **LRMS** (DEP/EI-Orbitrap): *m/z* (%): 455.3 (40).

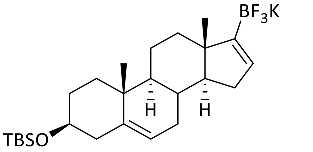

Potassium ((*3S,9S,10R,13S,14S*)-3-((*tert*-butyldimethylsilyl)oxy)-10,13-dimethyl-2,3,4,7,8,9,10,11,12,13,14,15-dodecahydro-1*H*-cyclopenta[*a*]phenanthren-17-yl)trifluoroborate (1o)

Using *tert*-butyl(((*3S,9S,10R,13S,14S*)-10,13-dimethyl-17-(4,4,5,5-tetramethyl-1,3,2-dioxaborolan-2-yl)-2,3,4,7,8,9,10,11,12,13,14,15-dodecahydro-1*H*-cyclopenta[*a*]phenanthren-3-yl)oxy)dimethylsilane according to general procedure **C**, provided **1o** (0.5 mmol, 246 mg, 50%) as colourless solid. Due to high insolubility in many different deuterated solvents like $CDCl_3$, DMSO-d_6, acetone-d_6, benzene-d_6, MeCN-d_3, NMR spectral analysis was not sufficient to determine the exact proton shifts and couplings. Nevertheless, an inaccurate proton NMR in MeCN-d_3 can be found in the NMR-spectra section.

^{11}B NMR (128 MHz, CD_3CN) δ 2.09 ppm (br, s). **HRMS** (ESI-Quadrupole): m/z: calcd for $C_{25}H_{41}BF_3OSi^-$ $[M-K]^-$: 453.2977; found: 453.2981. **IR** (Diamond-ATR, neat) $\tilde{\nu}_{max}$ (cm^{-1}): 2970 (w), 2960 (w), 2949

[11] J. Takagi, K. Takahashi, T. Ishiyama, N. Miyaura, *J. Am. Chem. Soc.* **2002**, *124*, 8001.

(w), 2930 (m), 2897 (w), 2857 (w), 2828 (w), 1608 (w), 1584 (w), 1470 (w), 1459 (w), 1437 (w), 1381 (w), 1368 (w), 1270 (w), 1250 (w), 1206 (w), 1195 (w), 1165 (w), 1146 (w), 1133 (w), 1087 (s), 1044 (w), 1004 (m), 993 (m), 982 (m), 952 (s), 945 (s), 930 (s), 919 (s), 908 (m), 887 (m), 874 (m), 863 (s), 838 (vs), 820 (m), 803 (m), 776 (s), 738 (m), 715 (w), 689 (w), 674 (w).Mp (°C) = >300.

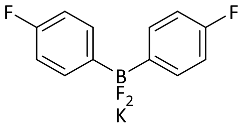

Potassium difluorobis(4-fluorophenyl) borate (1p)

Adapted from a previously reported procedure,[12] 15.0 mL of (4-fluorophenyl)magnesium bromide **A1** (0.7 M, 10.5 mmol, 2.1 equiv.) were cooled to 0 °C. Triisopropyl borate (5.0 mmol, 1.0 equiv., 1.15 mL) was slowly added via syringe pump over 30 minutes. The mixture was allowed to reach room temperature and stirred overnight. The slurry was then quenched with 2 mL of 2 M HCl and extracted with EtOAc (3 x 30 mL). The organic layers were combined, dried over $MgSO_4$ and concentrated in vacuo. After column chromatography in DCM (R_f = 0.6) the corresponding borinic acid was isolated as an orange oil. The crude was then directly redissolved in MeOH (20 mL) and KHF_2 (15.0 mmol, 1.17 g) was added in one portion at 0 °C. The reaction was stirred at room temperature overnight and the solvent removed afterwards. The solids were extracted with boiling acetone (4 x 20 mL) and the combined organic layers were dried in vacuo. The colourless solid was then filtered off and washed with Et_2O (3 x 20 mL) to yield the desired product **1p** (3.12 mmol, 867 mg, 62%).

^{1}H NMR (400 MHz, CD_3CN) δ 7.44 – 7.34 (m, 2H), 6.90 – 6.81 ppm (m, 2H). **^{13}C NMR** (101 MHz, CD_3CN) δ 162.2 (d, J = 237.8 Hz), 133.8 (dt, J = 6.9, 3.6 Hz), 113.7 ppm (d, J = 18.6 Hz). **^{11}B NMR** (128 MHz, CD_3CN) δ 6.84 ppm (t, J = 71.4 Hz). **HRMS** (ESI-Quadrupole): m/z: calcd for $C_{12}H_8BF_4^-$ [M-K]$^-$: 239.0655; found: 239.0660. **IR** (Diamond-ATR, neat) $\tilde{\nu}_{max}$ (cm^{-1}): 1594 (m), 1499 (w), 1389 (vw), 1301 (w), 1270 (w), 1228 (m), 1207 (m), 1193 (m), 1166 (m), 1158 (m), 1152 (m), 1090 (w), 1018 (w), 961 (w), 929 (m), 911 (m), 894 (s), 872 (m), 832 (s), 822 (vs), 800 (m), 715 (w). **Mp** (°C) = 193-196.

[12] T. Ito; T. Iwai; T. Mizuno; Y. Ishino, Synlett **2003**, *10*, 1435.

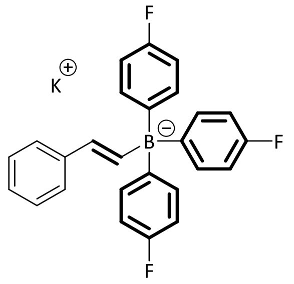

(*E*)-Tris(4-fluorophenyl)(styryl)borate (2a)

Using potassium (*E*)-trifluoro(styryl)borate **1a** and (4-fluorophenyl)magnesium bromide **A1** according to general procedure **E**, provided **2a** (2.61 mmol, 1.14 g, 87%) as colourless solid.

^{1}H NMR (400 MHz, (CD3)2CO) δ 7.59 (d, J = 17.8 Hz, 1H), 7.34 – 7.28 (m, 2H), 7.24 – 7.13 (m, 8H), 7.01 – 6.92 (m, 1H), 6.76 – 6.68 (m, 6H), 6.13 ppm (ddd, J = 17.8, 6.9, 3.3 Hz, 1H). **^{13}C NMR** (101 MHz, (CD3)2CO) δ 160.9 (d, J = 236.1 Hz), 160.1 (d, J = 3.4 Hz), 159.6 (d, J = 3.4 Hz), 159.1 (d, J = 4.0 Hz), 158.6 (d, J = 4.3 Hz), 158.0, 157.5, 143.7 (dd, J = 8.6, 4.3 Hz), 137.0 (ddd, J = 5.5, 3.5, 1.6 Hz), 131.6, 128.7, 126.0, 125.0, 112.5 ppm (ddd, J = 17.7, 6.1, 2.9 Hz). **^{11}B NMR** (128 MHz, CD_3CN) δ -9.49 ppm. **HRMS** (ESI-Quadrupole): m/z: calcd for $C_{26}H_{19}BF_3^-$ $[M\text{-}K]^-$: 399.1532; found: 399.1538. **IR** (Diamond-ATR, neat) $\tilde{\nu}_{max}$ (cm^{-1}): 1579 (m), 1487 (s), 1218 (s), 1157 (s), 1086 (w), 1012 (m), 966 (w), 827 (s), 817 (vs), 782 (w), 744 (m), 722 (m), 696 (m). **Mp** (°C) = 279-282 (decomposition).

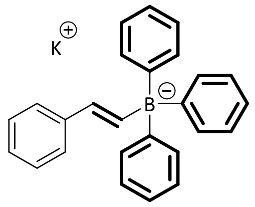

(*E*)-Tris(phenyl)(styryl)borate (2b)

Using potassium (*E*)-trifluoro(styryl)borate **1a** and phenylmagnesium bromide **A14** according to general procedure **E**, provided **2b** (2.47 mmol, 949 mg, 82%) as colourless powder.

^{1}H NMR (400 MHz, (CD3)2CO) δ 7.71 (d, J = 17.8 Hz, 1H), 7.34 – 7.27 (m, 8H), 7.18 – 7.11 (m, 2H), 6.99 – 6.92 (m, 7H), 6.83 – 6.77 (m, 3H), 6.20 ppm (ddd, J = 17.8, 6.7, 3.3 Hz, 1H). **^{13}C NMR** (101 MHz, (CD3)2CO) δ 165.5, 165.0, 164.5, 164.1, 160.4, 159.9, 159.4, 158.9, 144.2 (dd, J = 8.5, 4.1 Hz), 136.4 (dd, J = 2.9, 1.4 Hz), 131.2, 128.7, 126.2 (dd, J = 5.6, 2.7 Hz), 125.9, 124.6, 122.4 ppm. **^{11}B NMR** (128 MHz, CD_3CN) δ -8.75 ppm. **HRMS** (ESI-Quadrupole): m/z: calcd for $C_{26}H_{22}B^-$ $[M\text{-}K]^-$: 345.1815; found: 345.1824. **IR** (Diamond-ATR, neat) $\tilde{\nu}_{max}$ (cm^{-1}): 1742 (w), 1596 (w), 1578 (w), 1493 (w), 1477 (w), 1444 (w), 1428 (w), 1261 (w), 1236 (w), 1185 (w), 1152 (w), 1070 (w), 1030 (w), 1011 (w), 1006

(w), 958 (w), 912 (w), 874 (w), 818 (w), 768 (m), 756 (m), 739 (s), 723 (m), 712 (vs), 696 (s). **Mp** (°C) = >300.

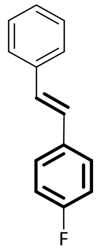

(*E*)-1-Fluoro-4-styrylbenzene (3a/3m)

Using potassium (*E*)-trifluoro(styryl)borate **1a** and (4-fluorophenyl)magnesium bromide **A1** according to general procedure **D**, provided **3a** (0.27 mmol, 54 mg, 68%, *E*/*Z* = 99:1) as colourless solid.

Using potassium (*Z*)-trifluoro(styryl)borate **1f** and (4-fluorophenyl)magnesium bromide **A1** according to general procedure **D**, provided **3m** (0.22 mmol, 44 mg, 55%, *E*/*Z* = 98:2) as colourless solid.

Using the purified tetraorganoborate **2a** under the optimized conditions for the electrochemical transformation, **3a** (0.30 mmol, 59 mg, 75%, *E*/*Z* = 99:1) was provided as a colourless solid.

Zweifel-Olefination:

A 25 mL Schlenk flask was charged with the (*Z*)-trifluoro(styryl)borate **1f** (0.2 mmol, 1.0 equiv., 42 mg) and 1 mL of THF was added. The mixture was cooled to 0 °C and the aryl-Grignard reagent **A1** (0.6 mmol, 3.0 equiv.) was added dropwise over 30 minutes *via* syringe pump. After addition, the reaction mixture was allowed to stir for further 10 min at 0 °C, after which 1.2 mL of NaOMe (0.5 M in MeOH, 0.6 mmol, 3.0 equiv.) were added. Iodine (0.3 mmol, 1.5 equiv., 76 mg) was dissolved in 1 mL THF and added dropwise to the mixture, which was then further stirred at 0 °C for 30 minutes. The slurry was then quenched with sat. aq. $Na_2S_2O_3$ solution (2 mL) and extracted with EtOAc (3 x 10 mL). The combined organic phases were dried over magnesium sulfate, filtered, concentrated under reduced pressure and purified by flash-column chromatography on silica gel in hexane (***R*f** = 0.37 (hexane/EtOAc 100:0, UV, $KMnO_4$)) to yield product **3a** (0.22 mmol, 44 mg, 55%, *E*/*Z* = 99:1) as colourless solid.

^{1}H NMR (400 MHz, $CDCl_3$) δ 7.46-7.35 (m, 4H), 7.27 (t, *J* = 7.7 Hz, 2H), 7.21-7.13 (m, 1H), 7.03-6.92 ppm (m, 4H). **^{13}C NMR** (101 MHz, $CDCl_3$) δ 162.34 (d, *J* = 247.2 Hz), 137.2, 133.51 (d, *J* = 3.3 Hz), 128.7, 128.49

(d, *J* = 2.5 Hz), 128.01 (d, *J* = 8.0 Hz), 127.7, 127.5, 126.5, 115.65 ppm (d, *J* = 21.6 Hz). **LRMS** (DEP/EI-Orbitrap): *m/z* (%): 198.0 (100), 183.0 (45), 177.0 (20). Analytical data in accordance to literature.[13]

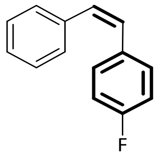

(*Z*)-1-Fluoro-4-styrylbenzene ((*Z*)-3a)

Zweifel-Olefination:

A 25 mL Schlenk flask was charged with (*E*)-Tris(4-fluorophenyl)(styryl)borate **2a** (0.2 mmol, 1.0 equiv., 88 mg) and 1 mL of THF was added. 1.2 mL of NaOMe (0.5 M in MeOH, 0.6 mmol, 3.0 equiv.) were added in one portion. Iodine (0.3 mmol, 1.5 equiv., 76 mg) was dissolved in 1 mL THF and added dropwise to the mixture, which was then further stirred at 0 °C for 30 minutes. The slurry was then quenched with sat. aq. $Na_2S_2O_3$ solution (2 mL) and extracted with EtOAc (3 x 10 mL). The combined organic phases were dried over magnesium sulfate, filtered, concentrated under reduced pressure and purified by flash-column chromatography on silica gel in hexane (R_f = 0.37 (hexane/EtOAc 100:0, UV, $KMnO_4$)) to yield product (*Z*)-**3a** (0.17 mmol, 34 mg, 86%, *E*/*Z* = 1:99) as colourless solid.

^{1}H NMR (400 MHz, $CDCl_3$) δ 7.28 – 7.16 (m, 7H), 6.95 – 6.87 (m, 2H), 6.60 (d, J = 12.2 Hz, 1H), 6.55 ppm (d, J = 12.2 Hz, 1H). **^{13}C NMR** (101 MHz, $CDCl_3$) δ 161.93 (d, J = 246.6 Hz), 137.15, 133.30 (d, J = 3.5 Hz), 130.65 (d, J = 7.9 Hz), 130.37 (d, J = 1.2 Hz), 129.20, 128.95, 128.44, 127.32, 115.28 ppm (d, J = 21.4 Hz). **LRMS** (DEP/EI-Orbitrap): *m/z* (%): 198.0 (100), 183.0 (45), 177.0 (20). Analytical data in accordance to literature.[14]

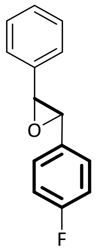

***trans*-2-(4-fluorophenyl)-3-phenyloxirane (3ab)**

Using potassium (*E*)-trifluoro(styryl)borate **1a** and (4-fluorophenyl)magnesium bromide **A1** according to general procedure **D under O_2 atmosphere**, provided **3ab** (0.15 mmol, 32 mg, 37) as yellowish oil.

[13] A. L. Isfahani; I. Mohammadpoor-Baltork; V. Mirkhani; A. R. Khosropour; M. Moghadam; S. Tangestaninejad; R. Kia, *Adv. Synth. Catal.* **2013**, *355*, 957.
[14] M. Das; D. F. O´Shea, Org. Lett. **2016**, *18*, 336.

R_f = 0.21 (hexane, UV, $KMnO_4$, PAA). **^{1}H NMR** (400 MHz, $CDCl_3$) δ 7.44 – 7.28 (m, 7H), 7.08 (tt, J = 8.8, 2.5 Hz, 2H), 3.85 ppm (dd, J = 9.4, 1.8 Hz, 2H). **^{13}C NMR** (101 MHz, $CDCl_3$) δ 162.90 (d, J = 246.6 Hz), 137.0, 132.98 (d, J = 3.0 Hz), 128.7, 128.6, 127.31 (d, J = 8.3 Hz), 125.6, 115.70 (d, J = 21.7 Hz), 63.0, 62.4 ppm. **LRMS** (EI pos): *m/z* (%): 214.0 (20), 196.0 (20), 185.0 (100), 165.0 (70). Analytical data in accordance to literature.[15]

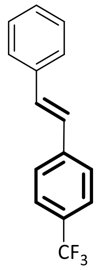

(*E*)-1-Styryl-4-(trifluoromethyl)benzene (3b)

Using potassium (*E*)-trifluoro(styryl)borate **1a** and (4-(trifluoromethyl)phenyl)magnesium bromide **A2** according to general procedure **D**, provided **3b** (0.28 mmol, 68 mg, 69%, *E/Z* = 99:1) as colourless solid.

^{1}H NMR (400 MHz, $CDCl_3$) δ 7.61 (s, 4H), 7.56-7.52 (m, 2H), 7.43-7.37 (m, 2H), 7.33-7.27 (m, 1H), 7.20 (d, *J* = 16.4 Hz, 1H), 7.12 ppm (d, *J* = 16.4 Hz, 1H). **^{13}C NMR** (101 MHz, $CDCl_3$) δ 140.9, 136.7, 131.3, 129.36 (q, *J* = 32.4 Hz), 128.9, 128.4, 127.2, 126.9, 126.7, 125.77 (q, *J* = 3.9 Hz), 123.0 ppm. **LRMS** (DEP/EI-Orbitrap): *m/z* (%): 248.0 (100), 233.0 (15), 227.0 (25). Analytical data in accordance to literature.[16]

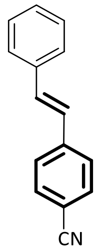

(*E*)-4-Styrylbenzonitrile (3c)

Using potassium (*E*)-trifluoro(styryl)borate **1a** and (4-cyanophenyl)zinc(II) iodide **B1** according to general procedure **D**, provided **3c** (0.12 mmol, 24 mg, 29%, *E/Z* = 99:1) as colourless solid.

^{1}H NMR (400 MHz, $CDCl_3$) δ 7.69-7.61 (m, 2H), 7.61-7.57 (m, 2H), 7.56-7.52 (m, 2H), 7.43-7.37 (m, 2H), 7.36-7.29 (m, 1H), 7.22 (d, *J* = 16.3 Hz, 1H), 7.09 ppm (d, *J* = 16.3 Hz, 1H). **^{13}C NMR** (101 MHz, $CDCl_3$) δ

15 T. Niwa, M. Nakada, *J. Am. Chem. Soc.* **2012**, *134*, 13538.
16 S. W. Youn; B. S. Kim; A. R. Jagdale, *J. Am. Chem. Soc.* **2012**, *134*, 11308.

141.9, 136.4, 132.6, 132.5, 129.0, 128.8, 127.0, 127.0, 126.8, 119.2, 110.7 ppm. **LRMS** (DEP/EI-Orbitrap): *m/z* (%): 205.1 (100), 190.0 (50), 176.0 (20). Analytical data in accordance to literature.[17]

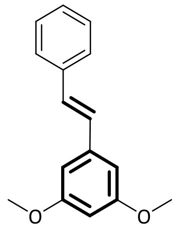

(*E*)-1,3-Dimethoxy-5-styrylbenzene (3d)

Using potassium (*E*)-(styryl)trifluoroborate **1a** and (3,5-dimethoxyphenyl)magnesium bromide **A3** according to general procedure **D**, provided **3d** (0.21 mmol, 50 mg, 42%) as colourless solid.

***R*f** = 0.50 (hexane/EtOAc 9:1, UV, $KMnO_4$). **¹H NMR** (400 MHz, $CDCl_3$) δ 7.54- 7.49 (m, 2H), 7.39- 7.34 (m, 2H), 7.30- 7.24 (m, 1H), 7.10 (d, *J* = 16.3 Hz, 1H), 7.04 (d, *J* = 16.3 Hz, 1H), 6.68 (d, *J* = 2.3 Hz, 2H), 6.40 (t, *J* = 2.3 Hz, 1H), 3.84 ppm (s, 6H). **¹³C NMR** (101 MHz, $CDCl_3$) δ 161.1, 139.5, 137.2, 129.3, 128.8, 128.8, 127.9, 126.7, 104.7, 100.1, 55.5 ppm. **LRMS** (DEP/EI-Orbitrap): *m/z* (%): 240.1 (100), 224.0 (10), 209.0 (20). **HRMS** (EI-Orbitrap): m/z: [M^+] Calcd. for $C_{16}H_{16}O_2^+$: 240.1150; found: 240.1143. Analytical data in accordance to literature.[18]

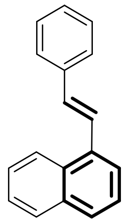

(*E*)-1-Styrylnaphthalene (3e/3q)

Using potassium (*E*)-trifluoro(styryl)borate **1a** and naphthalen-1-ylmagnesium bromide **A4** according to general procedure **D**, provided **3e** (0.30 mmol, 68 mg, 74%, *E/Z* = 99:1) as colourless solid.

Using potassium (*Z*)-trifluoro(styryl)borate **1f** and naphthalen-1-ylmagnesium bromide **A4** according to general procedure **D**, provided **3q** (0.17 mmol, 37 mg, 43%, *E/Z* = 93:7) as colourless solid.

¹H NMR (400 MHz, $CDCl_3$) δ 8.23 (d, *J* = 8.0 Hz, 1H), 7.93-7.86 (m, 2H), 7.82 (d, *J* = 8.2 Hz, 1H), 7.76 (d, *J* = 6.1 Hz, 1H), 7.65-7.60 (m, 2H), 7.58-7.48 (m, 3H), 7.42 (t, *J* = 7.7 Hz, 2H), 7.34-7.29 (m, 1H), 7.17 ppm (d, *J* = 16.0 Hz, 1H). **¹³C NMR** (101 MHz, $CDCl_3$) δ 137.7, 135.1, 133.8, 131.9, 131.5, 128.9, 128.8, 128.2,

[17] H. Li; J. Lü; J. Lin; Y. Huang; M. Cao; R. Cao, *Chem. Eur. J.* **2013**, *19*, 15661.
[18] J. Jang; C. Wang; Y. Sun; X. Man; J. Li; F. Sun, *Chem. Commun.* **2019**, *13*, 1903.

127.9, 126.8, 126.2, 126.0, 125.9, 125.8, 123.9, 123.8 ppm. **LRMS** (DEP/EI-Orbitrap): *m/z* (%): 229.1 (100), 215.1 (15), 202.1 (10). Analytical data in accordance to literature.[19]

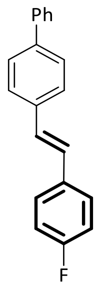

(*E*)-4-(4-Fluorostyryl)-1,1'-biphenyl (3f)

Using potassium (*E*)-(2-([1,1'-biphenyl]-4-yl)vinyl)trifluoroborate **1b** and (4-fluorophenyl)magnesium bromide **A1** according to general procedure **D**, provided **3f** (0.28 mmol, 78 mg, 71%, *E*/*Z* = 99:1) as colourless solid.

^{1}H NMR (400 MHz, $CDCl_3$) δ 7.69-7.55 (m, 6H), 7.55-7.42 (m, 4H), 7.40-7.31 (m, 1H), 7.15-7.00 ppm (m, 4H). **^{13}C NMR** (101 MHz, $CDCl_3$) δ 162.35 (d, *J* = 247.2 Hz), 140.6, 140.4, 136.2, 133.50 (d, *J* = 3.3 Hz), 128.8, 128.1, 128.0, 127.5, 127.4, 126.9, 126.9, 115.67 ppm (d, *J* = 21.6 Hz). **LRMS** (DEP/EI-Orbitrap): *m/z* (%): 274.1 (100), 259.0 (10), 252.1 (15). Analytical data in accordance to literature.[20]

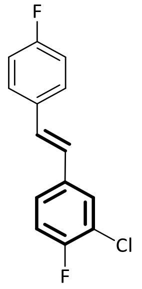

(*E*)-2-Chloro-1-fluoro-4-(4-fluorostyryl)benzene (3g)

Using potassium (*E*)-trifluoro(4-fluorostyryl)borate **1c** and (3-chloro-4-fluorophenyl)magnesium bromide **A5** according to general procedure **D**, provided **3g** (0.22 mmol, 55 mg, 55%, *E*/*Z* = 99:1) as colourless oil.

$\boldsymbol{R_f}$ = 0.40 (hexane, UV, $KMnO_4$). **^{1}H NMR** (400 MHz, $CDCl_3$) δ 7.53 (dd, *J* = 7.0, 2.2 Hz, 1H), 7.48-7.43 (m, 2H), 7.33 (ddd, *J* = 8.6, 4.6, 2.2 Hz, 1H), 7.12 (t, *J* = 8.7 Hz, 1H), 7.06 (t, *J* = 8.7 Hz, 2H), 6.99 (d, *J* = 16.3 Hz, 1H), 6.90 ppm (d, *J* = 16.3 Hz, 1H). **^{13}C NMR** (101 MHz, $CDCl_3$) δ 162.7 (d, *J* = 247.8 Hz), 157.6 (d, *J* = 249.8 Hz), 134.7 (d, *J* = 3.9 Hz), 133.0 (d, *J* = 3.4 Hz), 128.7 (d, *J* = 2.4 Hz), 128.3, 128.2, 126.2 (d, *J* = 7.0

[19] M. Das; D. F. O´Shea, *Org. Lett.* **2016**, *18*, 336.
[20] Y. Liu; P. Liu; Y. Wei, *Chin. J. Chem.* **2017**, *35*, 1141.

Hz), 126.1 (t, *J* = 2.1 Hz), 121.5 (d, *J* = 18.1 Hz), 116.9 (d, *J* = 21.5 Hz), 115.9 ppm (d, *J* = 21.7 Hz). **LRMS** (DEP/EI-Orbitrap): *m/z* (%): 250.0 (95), 235.0 (15), 214.1 (100), 195.1 (30). **HRMS** (EI-Orbitrap): m/z: [M$^+$] Calcd. for $C_{14}H_9ClF_2^+$: 250.0361; found: 250.0354. **IR** (Diamond-ATR, neat) $\tilde{\nu}_{max}$ (cm^{-1}): 2942 (vs), 2929 (vs), 2892 (s), 2866 (vs), 1502 (vs), 1463 (s), 1254 (m), 1202 (s), 1148 (m), 1096 (s), 1059 (m), 1037 (s), 1017 (s), 1004 (s), 993 (s), 981 (s), 942 (m), 931 (m), 919 (m), 906 (m), 884 (s), 863 (m), 830 (s), 810 (m), 804 (m), 775 (m), 739 (m), 709 (m), 677 (s), 668 (s), 662 (s).

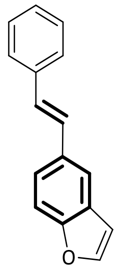

(*E*)-5-Styrylbenzofuran (3h)

Using potassium (*E*)-trifluoro(styryl)borate **1a** and benzofuran-5-ylmagnesium bromide **A6** according to general procedure **D**, provided **3h** (0.25 mmol, 55 mg, 63%, *E*/*Z* = 99:1) as colourless oil.

***R*$_f$** = 0.3 (hexane, UV, PAA, $KMnO_4$). **^{1}H NMR** (400 MHz, $CDCl_3$) δ 7.72 (s, 1H), 7.62 (d, *J* = 2.2 Hz, 1H), 7.55-7.47 (m, 4H), 7.37 (t, *J* = 7.7 Hz, 2H), 7.31-7.19 (m, 2H), 7.10 (d, *J* = 16.3 Hz, 1H), 6.78 ppm (d, *J* = 2.2 Hz, 1H). **^{13}C NMR** (101 MHz, $CDCl_3$) δ 154.8, 145.7, 137.6, 132.6, 129.1, 128.8, 128.0, 127.8, 127.5, 126.5, 123.1, 119.4, 111.7, 106.8 ppm. **LRMS** (DEP/EI-Orbitrap): *m/z* (%): 220.0 (100), 204.9 (10), 191.0 (60). **HRMS** (EI-Orbitrap): m/z: [M$^+$] Calcd. for $C_{16}H_{12}O^+$: 220.0888; found: 220.0882. **IR** (Diamond-ATR, neat) $\tilde{\nu}_{max}$ (cm^{-1}): 1464 (w), 1450 (w), 1253 (w), 1197 (w), 1125 (m), 1105 (m), 1028 (m), 967 (m), 887 (m), 809 (s), 769 (s), 736 (vs), 693 (s).

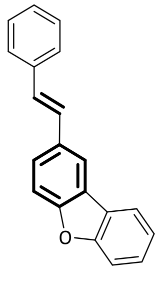

(*E*)-2-Styryldibenzo[*b*,*d*]furan (3i/3s)

Using potassium (*E*)-trifluoro(styryl)borate **1a** and dibenzo[*b*,*d*]furan-2-ylmagnesium bromide **A7** according to general procedure **D**, provided **3i** (0.23 mmol, 62 mg, 57%, *E*/*Z* = 99:1) as colourless solid.

Using potassium (Z)-trifluoro(styryl)borate **1f** and dibenzo[*b*,*d*]furan-2-ylmagnesium bromide **A7** according to general procedure **D**, provided **3s** (0.24 mmol, 65 mg, 60%, *E*/*Z* = 96:4) as colourless solid.

^{1}**H NMR** (400 MHz, $CDCl_3$) δ 8.09 (d, *J* = 1.8 Hz, 1H), 7.99 (dd, *J* = 7.8, 1.3 Hz, 1H), 7.64 (dd, *J* = 8.5, 1.8 Hz, 1H), 7.61-7.53 (m, 4H), 7.48 (ddd, *J* = 8.4, 7.3, 1.4 Hz, 1H), 7.43-7.35 (m, 3H), 7.32-7.25 (m, 2H), 7.17 ppm (d, *J* = 16.3 Hz, 1H). ^{13}**C NMR** (101 MHz, $CDCl_3$) δ 156.8, 156.0, 137.5, 132.6, 128.9, 128.7, 128.1, 127.6, 127.5, 126.5, 126.1, 124.8, 124.2, 123.0, 120.9, 118.6, 111.9, 111.9 ppm. **LRMS** (DEP/EI-Orbitrap): *m/z* (%): 270.1 (100), 255.0 (10), 239.0 (20). Analytical data in accordance to literature.[21]

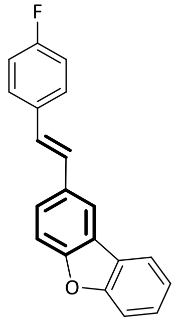

(*E*)-2-(4-Fluorostyryl)dibenzo[*b*,*d*]furan (3j)

Using potassium (*E*)-trifluoro(4-fluorostyryl)borate **1c** and dibenzo[*b*,*d*]furan-2-ylmagnesium bromide **A7** according to general procedure **D**, provided **3j** (0.27 mmol, 78 mg, 68%, *E/Z* = 99:1) as colourless oil.

$\boldsymbol{R_f}$ = 0.30 (hexane, UV, PAA, $KMnO_4$). ^{1}**H NMR** (400 MHz, $CDCl_3$) δ 8.07 (d, *J* = 1.8 Hz, 1H), 7.98 (d, *J* = 6.9 Hz, 1H), 7.62 (dd, *J* = 8.6, 1.8 Hz, 1H), 7.60-7.45 (m, 5H), 7.37 (td, *J* = 7.5, 1.0 Hz, 1H), 7.18 (d, *J* = 16.3 Hz, 1H), 7.14-7.05 ppm (m, 3H). ^{13}**C NMR** (101 MHz, $CDCl_3$) δ 162.40 (d, *J* = 246.9 Hz), 156.8, 156.0, 133.74 (d, *J* = 3.3 Hz), 132.4, 128.53 (d, *J* = 2.5 Hz), 128.00 (d, *J* = 7.8 Hz), 127.5, 126.9, 126.0, 124.9, 124.2, 123.0, 120.8, 118.5, 115.9, 115.7, 111.95 ppm (d, *J* = 4.4 Hz). **LRMS** (DEP/EI-Orbitrap): *m/z* (%): 288.1 (100), 273.0 (5), 257.1 (30). **HRMS** (EI-Orbitrap): m/z: [M^+] Calcd. for $C_{20}H_{13}FO^+$: 288.0950; found: 288.0945. **IR** (Diamond-ATR, neat) $\tilde{\nu}_{max}$ (cm^{-1}): 3041 (w), 1710 (m), 1601 (m), 1508 (s), 1473 (m), 1450 (s), 1431 (m), 1414 (m), 1360 (m), 1349 (m), 1329 (w), 1304 (w), 1296 (w), 1261 (w), 1226 (s), 1210 (m), 1196 (s), 1168 (m), 1159 (m), 1141 (m), 1122 (m), 1100 (m), 1022 (m), 1004 (w), 972 (m), 961 (s), 940 (m), 926 (m), 908 (w), 893 (m), 857 (m), 841 (m), 824 (vs), 812 (s), 790 (m), 766 (m), 752 (s), 741 (vs), 726 (s), 710 (m), 683 (w), 665 (w), 656 (w).

---

[21] C. Wang; I. Piel; F. Glorius, *J. Am. Chem. Soc.* **2009**, *131*, 4194.

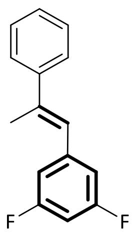

(*E*)-1,3-Difluoro-5-(2-phenylprop-1-en-1-yl)benzene (3k)

Using potassium (*E*)-trifluoro(2-phenylprop-1-en-1-yl)borate **1d** and (3,5-difluorophenyl)magnesium bromide **A8** according to general procedure **D**, provided **3k** (0.28 mmol, 64 mg, 70%, *E/Z* = 99:1) as colourless oil.

$\boldsymbol{R}_f$ = 0.6 (hexane, UV, PAA, $KMnO_4$). **^{1}H NMR** (400 MHz, $CDCl_3$) δ 7.56-7.46 (m, 2H), 7.43-7.36 (m, 2H), 7.35-7.29 (m, 1H), 6.94-6.83 (m, 2H), 6.74-6.66 (m, 2H), 2.28 (d, *J* = 1.4 Hz, 3H). **^{13}C NMR** (101 MHz, $CDCl_3$) δ 162.97 (dd, *J* = 247.4, 13.2 Hz), 143.4, 141.62 (t, *J* = 9.7 Hz), 140.1, 128.6, 127.9, 126.2, 125.80 (t, *J* = 2.6 Hz), 112.21-111.70 (m), 102.01 (t, *J* = 25.5 Hz), 17.8 ppm. **LRMS** (DEP/EI-Orbitrap): *m/z* (%): 230.1 (100), 215.1 (80), 195.1 (20). **HRMS** (EI-Orbitrap): m/z: [M^+] Calcd. for $C_{14}H_{15}FO_2^+$: 230.0907; found: 230.0896.

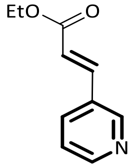

Ethyl (*E*)-3-(pyridin-3-yl)acrylate (3l)

Using potassium (*E*)-(3-ethoxy-3-oxoprop-1-en-1-yl)trifluoroborate **1e** and pyridin-3-ylzinc(II) iodide **B2** according to general procedure **D**, provided **3l** (0.10 mmol, 18 mg, 25%, *E/Z* = 99:1) as colourless oil.

$\boldsymbol{R}_f$ = 0.25 (hexane/EtOAc 6:4, UV, PAA). **^{1}H NMR** (400 MHz, $CDCl_3$) δ 8.75 (d, *J* = 2.2 Hz, 1H), 8.61 (dd, *J* = 4.8, 1.6 Hz, 1H), 7.84 (dt, *J* = 7.9, 2.0 Hz, 1H), 7.67 (d, *J* = 16.1 Hz, 1H), 7.37-7.31 (m, 1H), 6.51 (d, *J* = 16.1 Hz, 1H), 4.28 (q, *J* = 7.1 Hz, 2H), 1.35 ppm (t, *J* = 7.1 Hz, 3H). **^{13}C NMR** (101 MHz, $CDCl_3$) δ 166.5, 151.1, 149.9, 141.0, 134.3, 130.4, 123.9, 120.6, 61.0, 14.4 ppm. **HRMS** (EI-Orbitrap): m/z: [M^+] Calcd. for $C_{10}H_{11}NO_2^+$: 177.0790; found: 177.0782. **IR** (Diamond-ATR, neat) $\tilde{\nu}_{max}$ (cm^{-1}): 2963 (m), 2956 (m), 2926 (m), 2853 (m), 1717 (vs), 1685 (w), 1653 (w), 1642 (m), 1508 (m), 1472 (m), 1465 (m), 1457 (m), 1418 (m), 1388 (w), 1367 (m), 1312 (m), 1278 (m), 1262 (s), 1218 (m), 1184 (s), 1127 (m), 1120 (m), 1096 (m), 1074 (m), 1066 (m), 1043 (m), 1026 (m), 983 (m), 806 (m), 718 (w), 712 (w), 700 (m), 668 (w), 662 (w).

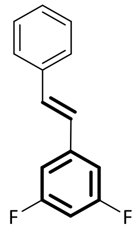

(*E*)-1,3-difluoro-5-styrylbenzene (3n)

Using potassium (*Z*)-trifluoro(styryl)borate **1f** and (3,5-difluorophenyl)magnesium bromide **A8** according to general procedure **D**, provided **3n** (0.26 mmol, 56 mg, 65%, *E*/*Z* = 90:10) as colourless solid.

^{1}H NMR (400 MHz, $CDCl_3$) δ 7.56-7.48 (m, 2H), 7.43-7.35 (m, 2H), 7.35-7.28 (m, 1H), 7.11 (d, *J* = 16.2 Hz, 1H), 7.05-6.97 (m, 3H), 6.71 ppm (tt, *J* = 8.8, 2.3 Hz, 1H). **^{13}C NMR** (101 MHz, $CDCl_3$) δ 163.41 (dd, *J* = 247.5, 13.2 Hz), 140.88 (t, *J* = 9.6 Hz), 136.4, 131.4, 129.0, 128.5, 126.9, 126.62 (t, *J* = 2.9 Hz), 109.46-108.89 (m), 102.85 ppm (t, *J* = 25.7 Hz). **LRMS** (DEP/EI-Orbitrap): *m/z* (%): 216.1 (100), 201.1 (50), 195.1 (30). Analytical data in accordance to literature.[22]

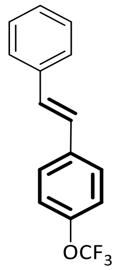

(*E*)-1-Styryl-4-(trifluoromethoxy)benzene (3o)

Using potassium (*Z*)-trifluoro(styryl)borate **1f** and (4-(trifluoromethoxy)phenyl)magnesium bromide **A9** according to general procedure **D**, provided **3o** (0.24 mmol, 64 mg, 60%, *E*/*Z* = 97:3) as colourless solid.

^{1}H NMR (400 MHz, $CDCl_3$) δ 7.57-7.49 (m, 4H), 7.42-7.34 (m, 2H), 7.33-7.27 (m, 1H), 7.24-7.19 (m, 2H), 7.09 ppm (s, 2H). **^{13}C NMR** (101 MHz, $CDCl_3$) δ 148.6, 137.0, 136.2, 129.8, 128.9, 128.1, 127.8, 127.2, 126.7, 121.3, 120.6 ppm (q, *J* = 257.1 Hz). **LRMS** (DEP/EI-Orbitrap): *m/z* (%): 264.1 (100), 249.0 (10), 179.1 (50). Analytical data in accordance to literature.[23]

---

[22] T. Ismail; S. Shafi; J. Srinivas; D. Sarkar; Y. Qurishi; J. Khazir; M. S. Alam; H. M. S. Kumar, *Bioorg. Chem.* **2016**, *64*, 97.
[23] K. Kanagaraj; K. Pitchumani, *Chem. Eur. J.* **2013**, *19*, 14425.

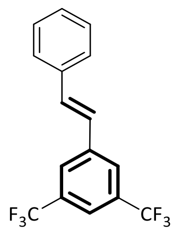

(*E*)-1-Styryl-3,5-bis(trifluoromethyl)benzene (3p)

Using potassium (*Z*)-trifluoro(styryl)borate **1f** and (3,5-bis(trifluoromethyl)phenyl) magnesium bromide **A10** according to general procedure **D**, provided **3p** (0.24 mmol, 76 mg, 60%, *E*/*Z* = 90:10) as colourless solid.

^{1}H NMR (400 MHz, $CDCl_3$) δ 7.92 (s, 2H), 7.75 (s, 1H), 7.59-7.53 (m, 2H), 7.46-7.38 (m, 2H), 7.38-7.31 (m, 1H), 7.25 (d, *J* = 16.4 Hz, 1H), 7.13 ppm (d, *J* = 16.4 Hz, 1H). **^{13}C NMR** (101 MHz, $CDCl_3$) δ 139.5, 136.1, 132.6, 132.1 (q, *J* = 33.0 Hz), 129.1, 128.9, 127.1, 126.3 (d, *J* = 3.9 Hz), 125.6, 123.5 (q, *J* = 272.8 Hz), 120.9 ppm (p, *J* = 3.9 Hz). **LRMS** (DEP/EI-Orbitrap): *m/z* (%): 316.1 (100), 301.1 (15), 275.1 (10). Analytical data in accordance to literature.[24]

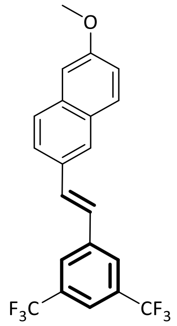

(*E*)-2-(3,5-Bis(trifluoromethyl)styryl)-6-methoxynaphthalene (3r)

Using potassium (*Z*)-trifluoro(2-(6-methoxynaphthalen-2-yl)vinyl)borate **1g** and (3,5-bis(trifluoromethyl)phenyl) magnesium bromide **A10** according to general procedure **D**, provided **3r** (0.22 mmol, 89 mg, 56%, *E*/*Z* = 84:16) as colourless oil.

***R*f** = 0.25 (hexane, UV, PAA, $KMnO_4$). **^{1}H NMR** (400 MHz, $CDCl_3$) δ 7.94 (s, 2H), 7.85 (s, 1H), 7.77-7.68 (m, 4H), 7.38 (d, *J* = 16.3 Hz, 1H), 7.24 (d, *J* = 15.1 Hz, 1H), 7.19-7.12 (m, 2H), 3.94 ppm (s, 3H). **^{13}C NMR** (101 MHz, $CDCl_3$) δ 158.4, 139.8, 134.9, 132.8, 132.13 (q, *J* = 33.1 Hz), 131.5, 129.9, 129.1, 127.8, 127.6, 126.2, 124.8, 123.9, 122.2, 120.81-120.57 (m), 119.5, 106.1 ppm. **LRMS** (DEP/EI-Orbitrap): *m/z* (%): 396.1 (100), 381.0 (5), 353.0 (10), 333.0 (5). **HRMS** (EI-Orbitrap): m/z: [M^+] Calcd. for $C_{21}H_{14}F_6O_2^+$: 396.0949; found: 396.0949. **IR** (Diamond-ATR, neat) $\tilde{\nu}_{max}$ (cm^{-1}): 1626 (m), 1611 (m), 1602 (m), 1484

[24] L. Yu; Y. Huang; Z. Wie; Y. Ding; C. Su; Q. Xu, *J. Org. Chem.* **2015**, *80*, 8677.

(m), 1467 (w), 1392 (m), 1373 (s), 1274 (vs), 1248 (m), 1218 (w), 1204 (m), 1170 (s), 1123 (vs), 1107 (s), 1032 (m), 1000 (w), 957 (m), 943 (m), 888 (s), 853 (m), 844 (m), 810 (m), 699 (m), 682 (s), 666 (m).

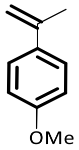

1-Methoxy-4-(prop-1-en-2-yl)benzene (4a)

Using potassium trifluoro(prop-1-en-2-yl)borate **1h** and (4-methoxyphenyl)magnesium bromide **A11** according to general procedure **D**, provided **4a** (0.16 mmol, 24 mg, 41%) as colourless oil.

^{1}H NMR (400 MHz, $CDCl_3$) δ 7.45-7.37 (m, 2H), 6.91-6.82 (m, 2H), 5.29 (dd, *J* = 1.6, 0.8 Hz, 1H), 5.04-4.96 (m, 1H), 3.82 (s, 3H), 2.13 ppm (dd, *J* = 1.5, 0.8 Hz, 3H). **^{13}C NMR** (101 MHz, $CDCl_3$) δ 158.5, 142.7, 133.9, 126.7, 113.7, 111.3, 56.1, 22.1 ppm. **LRMS** (DEP/EI-Orbitrap): *m/z* (%): 148.0 (100), 133.0 (80), 127.8 (5). Analytical data in accordance to literature.[25]

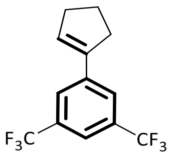

1-(Cyclopent-1-en-1-yl)-3,5-bis(trifluoromethyl)benzene (4b)

Using potassium cyclopent-1-en-1-yltrifluoroborate **1i** and (3,5-bis(trifluoromethyl)phenyl)magnesium bromide **A10** according to general procedure **D**, provided **4b** (0.21 mmol, 58 mg, 52%) as colourless oil.

***R*$_f$** = 0.6 (hexane, UV, PAA). **^{1}H NMR** (400 MHz, $CDCl_3$) δ 7.81 (s, 2H), 7.70 (s, 1H), 6.39 (td, *J* = 2.7, 1.4 Hz, 1H), 2.78-2.71 (m, 2H), 2.62-2.56 (m, 2H), 2.08 ppm (p, *J* = 7.6 Hz, 2H). **^{13}C NMR** (101 MHz, $CDCl_3$) δ 140.3, 138.9, 131.6 (q, *J* = 32.9 Hz), 130.7, 125.5, 123.5 (q, *J* = 272.5 Hz), 120.3 (q, *J* = 3.9 Hz), 33.7, 33.2, 23.4 ppm. **LRMS** (DEP/EI-Orbitrap): *m/z* (%): 280.1 (100), 261.1 (50), 245.1 (40), 211.1 (100), 191.1 (45), 142.1 (25). **HRMS** (EI-Orbitrap): m/z: [M^+] Calcd. for $C_{13}H_{10}F_6^+$: 280.0687; found: 280.0680. **IR** (Diamond-ATR, neat) $\tilde{\nu}_{max}$ (cm^{-1}): 2959 (vw), 2929 (vw), 2852 (vw), 1703 (vw), 1626 (vw), 1468 (w), 1384 (m), 1331 (w), 1277 (vs), 1171 (s), 1129 (vs), 1108 (m), 1046 (w), 1016 (w), 994 (w), 960 (w), 945 (vw), 895 (m), 888 (m), 843 (w), 770 (vw), 758 (vw), 700 (m), 682 (m).

---

[25] W. J. Kerr; A. J. Morrison; M. Pazicky; T. Weber, *Org. Lett.* **2012**, *14*, 2250.

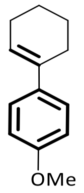

4'-Methoxy-2,3,4,5-tetrahydro-1,1'-biphenyl (4c)

Using potassium cyclohex-1-en-1-yltrifluoroborate **1j** and (4-methoxyphenyl)magnesium bromide **A11** according to general procedure **D**, provided **4c** (0.28 mmol, 53 mg, 71%) as colourless oil.

^{1}H NMR (400 MHz, $CDCl_3$) δ 7.36-7.29 (m, 2H), 6.90-6.82 (m, 2H), 6.03 (tt, *J* = 3.9, 1.7 Hz, 1H), 3.81 (s, 3H), 2.38 (dtd, *J* = 6.1, 3.2, 2.6, 1.4 Hz, 2H), 2.19 (dddd, *J* = 8.7, 6.3, 4.4, 2.5 Hz, 2H), 1.82-1.72 (m, 2H), 1.68-1.61 ppm (m, 2H). **^{13}C NMR** (101 MHz, $CDCl_3$) δ 158.5, 136.0, 135.5, 126.0, 123.3, 113.6, 55.4, 27.6, 26.0, 23.2, 22.3 ppm. **LRMS** (DEP/EI-Orbitrap): *m/z* (%): 188.1 (100), 184.0 (5), 173.1 (15), 159.0 (50). Analytical data in accordance to literature.[26]

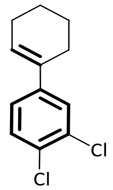

3',4'-Dichloro-2,3,4,5-tetrahydro-1,1'-biphenyl (4d)

Using potassium cyclohex-1-en-1-yltrifluoroborate **1j** and (3,4-dichlorophenyl)magnesium bromide **A12** according to general procedure **D**, provided **4d** (0.21 mmol, 48 mg, 53%) as colourless oil.

R_f = 0.80 (hexane, UV, PAA, $KMnO_4$). **^{1}H NMR** (400 MHz, $CDCl_3$) δ 7.44 (d, *J* = 2.2 Hz, 1H), 7.35 (d, *J* = 8.4 Hz, 1H), 7.20 (dd, *J* = 8.4, 2.2 Hz, 1H), 6.13 (td, *J* = 4.0, 1.9 Hz, 1H), 2.33 (tdd, *J* = 6.2, 2.5, 1.7 Hz, 2H), 2.20 (dddd, *J* = 9.2, 6.7, 4.7, 2.7 Hz, 2H), 1.82-1.72 (m, 2H), 1.70-1.58 ppm (m, 2H). **^{13}C NMR** (101 MHz, $CDCl_3$) δ 142.0, 134.8, 132.3, 130.2, 130.1, 127.0, 126.7, 123.5, 27.3, 26.0, 23.0, 22.0 ppm. **LRMS** (DEP/EI-Orbitrap): *m/z* (%): 226.0 (70), 211.0 (20), 191.0 (60), 163.0 (100). **HRMS** (EI-Orbitrap): m/z: [M^+] Calcd. for $C_{12}H_{12}Cl_2^+$: 226.0316; found: 226.0309. **IR** (Diamond-ATR, neat) $\tilde{\nu}_{max}$ (cm^{-1}): 2927 (w), 2848 (w), 1724 (w), 1627 (w), 1463 (w), 1447 (w), 1425 (w), 1384 (w), 1363 (w), 1353 (w), 1311 (w), 1279 (w), 1261 (w), 1236 (m), 1128 (vs), 1076 (w), 1042 (m), 969 (m), 947 (m), 916 (w), 904 (m), 863 (w), 844 (m), 802 (w), 773 (w), 762 (w), 746 (w).

---

[26] M. O. Ganiu; A- H. Cleveland; J. L. Paul; R. Kartika, *Org. Lett.* **2019**, *21*, 5611.

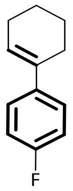

4'-Fluoro-2,3,4,5-tetrahydro-1,1'-biphenyl (4e)

Using potassium cyclohex-1-en-1-yltrifluoroborate **1j** and (4-fluorophenyl)magnesium bromide **A1** according to general procedure, provided **4e** (0.30 mmol, 53 mg, 75%) as colourless oil.

^{1}**H NMR** (400 MHz, $CDCl_3$) δ 7.41-7.29 (m, 2H), 7.03-6.92 (m, 2H), 6.06 (tt, *J* = 3.9, 1.8 Hz, 1H), 2.37 (ddq, *J* = 6.3, 4.3, 2.2 Hz, 2H), 2.20 (dtt, *J* = 8.8, 6.1, 2.6 Hz, 2H), 1.85-1.74 (m, 2H), 1.70-1.60 ppm (m, 2H). ^{13}**C NMR** (101 MHz, $CDCl_3$) δ 161.75 (d, *J* = 244.9 Hz), 138.8, 135.6, 126.37 (d, *J* = 7.7 Hz), 124.7, 114.88 (d, *J* = 21.2 Hz).,27.5, 25.8, 23.0, 22.1 ppm. **LRMS** (DEP/EI-Orbitrap): *m/z* (%): 176.1 (100), 161.0 (50), 147.0 (100). Analytical data in accordance to literature.[27]

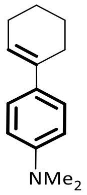

4'-Fluoro-2,3,4,5-tetrahydro-1,1'-biphenyl (4f)

Using potassium cyclohex-1-en-1-yltrifluoroborate **1j** and (4-(dimethylamino)phenyl)magnesium bromide **A13** according to general procedure **D**, provided **4f** (0.20 mmol, 40 mg, 70%) as colourless oil.

^{1}**H NMR** (400 MHz, $CDCl_3$) δ 7.34-7.27 (m, 2H), 6.74-6.65 (m, 2H), 6.00 (tt, *J* = 4.0, 1.7 Hz, 1H), 2.38 (ddt, *J* = 6.2, 3.9, 2.0 Hz, 2H), 2.19 (dtd, *J* = 8.2, 4.0, 2.2 Hz, 2H), 1.82-1.72 (m, 2H), 1.69-1.59 ppm (m, 2H). ^{13}**C NMR** (101 MHz, $CDCl_3$) δ 149.6, 136.1, 131.3, 125.7, 121.7, 112.6, 40.9, 27.5, 26.0, 23.3, 22.5 ppm. **LRMS** (DEP/EI-Orbitrap): *m/z* (%): 201.1 (100), 197.1 (40), 186.1 (5). Analytical data in accordance to literature.[28]

[27] K. Ishizuka; H. Seike; T. Hatakeyama; M. Nakamura, *J. Am. Chem. Soc.* **2010**, *132*, 13117.
[28] W. D. Oosterbaan; P. C. M. van Gerven; C. A. van Walree; M. Koeberg; J. J. Piet; R. W. A. Havenith; J. W. Zwikker; L. W. Jenneskens; R. Gleiter, *Eur. J. Org. Chem.* **2003**, 3117.

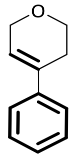

4-Phenyl-3,6-dihydro-2*H*-pyran (4g)

Using potassium (3,6-dihydro-2*H*-pyran-4-yl)trifluoroborate **1k** and phenylmagnesium bromide **A14** according to general procedure **D**, provided **4g** (0.24 mmol, 38 mg, 60%) as colourless oil.

^{1}H NMR (400 MHz, $CDCl_3$) δ 7.42-7.38 (m, 2H), 7.37-7.32 (m, 2H), 7.30-7.24 (m, 1H), 6.13 (tt, *J* = 3.0, 1.6 Hz, 1H), 4.33 (q, *J* = 2.8 Hz, 2H), 3.94 (t, *J* = 5.5 Hz, 2H), 2.74-2.34 ppm (m, 2H). **^{13}C NMR** (101 MHz, $CDCl_3$) δ 140.4, 134.2, 128.6, 127.4, 124.8, 122.6, 66.0, 64.6, 27.3 ppm. **LRMS** (DEP/EI-Orbitrap): *m/z* (%): 160.1 (100), 145.1 (10), 131.1 (100). Analytical data in accordance to literature.[29]

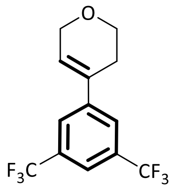

4-(3,5-Bis(trifluoromethyl)phenyl)-3,6-dihydro-2H-pyran (4h)

Using potassium (3,6-dihydro-2*H*-pyran-4-yl)trifluoroborate **1k** and (3,5-bis(trifluoromethyl)phenyl) magnesium bromide **A10** according to general procedure **D**, provided **4h** (0.22 mmol, 64 mg, 54%) as colourless oil.

R_f = 0.3 (hexane/EtOAc 9:1, UV, PAA, $KMnO_4$). **^{1}H NMR** (400 MHz, $CDCl_3$) δ 7.80 (s, 2H), 7.76 (s, 1H), 6.31 (tt, *J* = 3.0, 1.6 Hz, 1H), 4.36 (q, *J* = 2.9 Hz, 2H), 3.96 (t, *J* = 5.4 Hz, 2H), 2.59-2.51 ppm (m, 2H). **^{13}C NMR** (101 MHz, $CDCl_3$) δ 142.3, 132.3, 131.89 (q, *J* = 33.1 Hz), 126.3, 125.05-124.76 (m), 123.51 (d, *J* = 271.9 Hz), 121.09-120.83 (m), 65.8, 64.2, 27.1 ppm. **LRMS** (DEP/EI-Orbitrap): *m/z* (%): 296.1 (100), 278.1 (70), 267.1 (80), 254.1 (20). **HRMS** (EI-Orbitrap): m/z: [M^+] Calcd. for $C_{13}H_{10}F_6^+$: 296.0636; found: 296.0628. **IR** (Diamond-ATR, neat) $\tilde{\nu}_{max}$ (cm^{-1}): 1710 (m), 1621 (vw), 1470 (vw), 1356 (m), 1275 (vs), 1224 (w), 1171 (s), 1123 (vs), 1053 (m), 1021 (m), 963 (w), 940 (m), 899 (m), 879 (w), 844 (m), 810 (w), 725 (w), 701 (m), 681 (s).

[29] B. Guo; G. Schwarzwalder; J. T. Njardarson, *Angew. Chem. Int. Ed.* **2012**, *51*, 5675.

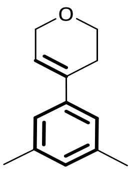

4-(3,5-Dimethylphenyl)-3,6-dihydro-2H-pyran (4i)

Using potassium (3,6-dihydro-2*H*-pyran-4-yl)trifluoroborate **1k** and (3,5-dimethylphenyl)magnesium bromide **A15** according to general procedure **D**, provided **4i** (0.22 mmol, 64 mg, 54%) as colourless oil.

$\boldsymbol{R_f}$ = 0.35 (hexane/EtOAc 98:2, UV, $KMnO_4$). **^{1}H NMR** (400 MHz, $CDCl_3$) δ 7.02 (s, 2H), 6.92 (s, 1H), 6.09 (tt, *J* = 3.1, 1.6 Hz, 1H), 4.32 (q, *J* = 2.8 Hz, 2H), 3.93 (t, *J* = 5.5 Hz, 2H), 2.54-2.48 (m, 2H), 2.33 ppm (s, 6H). **^{13}C NMR** (101 MHz, $CDCl_3$) δ 140.5, 138.0, 134.4, 129.1, 122.8, 122.2, 66.0, 64.7, 27.5, 21.5 ppm. **LRMS** (DEP/EI-Orbitrap): *m/z* (%): 188.1 (100), 173.1 (90), 159.1 (35), 145.1 (100). **HRMS** (EI-Orbitrap): m/z: [M^+] Calcd. for $C_{13}H_{16}O^+$: 188.1201; found: 188.1206. **IR** (Diamond-ATR, neat) $\tilde{\nu}_{max}$ (cm^{-1}): 2948 (m), 2920 (s), 2894 (m), 1722 (s), 1685 (m), 1602 (s), 1464 (m), 1450 (m), 1444 (m), 1423 (m), 1385 (m), 1363 (m), 1309 (m), 1295 (m), 1280 (m), 1269 (m), 1260 (m), 1242 (m), 1223 (m), 1183 (m), 1160 (m), 1137 (vs), 1084 (s), 1060 (s), 1044 (s), 1017 (m), 995 (m), 974 (m), 966 (m), 951 (s), 881 (m), 849 (s), 818 (m), 699 (m), 688 (m).

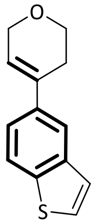

4-(Benzo[*b*]thiophen-5-yl)-3,6-dihydro-2*H*-pyran (4j)

Using potassium (3,6-dihydro-2*H*-pyran-4-yl)trifluoroborate **1k** and benzo[*b*]thiophen-5-ylmagnesium bromide **A16** according to general procedure **D**, provided **4j** (0.09 mmol, 20 mg, 23%) as colourless oil.

$\boldsymbol{R_f}$ = 0.60 (hexane/EtOAc 98:2, UV, PAA, $KMnO_4$). **^{1}H NMR** (400 MHz, $CDCl_3$) δ 7.74-7.68 (m, 2H), 7.37-7.29 (m, 2H), 7.23 (d, *J* = 5.5 Hz, 1H), 6.09 (tt, *J* = 3.0, 1.6 Hz, 1H), 4.26 (q, *J* = 2.8 Hz, 2H), 3.88 (t, *J* = 5.5 Hz, 2H), 2.59-2.46 ppm (m, 2H). **^{13}C NMR** (101 MHz, $CDCl_3$) δ 140.0, 138.7, 136.9, 134.3, 124.2, 122.6, 122.4, 121.7, 119.7, 66.1, 64.7, 27.7 ppm. **HRMS** (EI-Orbitrap): m/z: [M^+] Calcd. for $C_{13}H_{12}OS^+$: 216.0609; found: 216.0604. **IR** (Diamond-ATR, neat) $\tilde{\nu}_{max}$ (cm^{-1}): 3253 (w), 3099 (w), 2922 (m), 2853 (m), 1596 (m), 1565 (w), 1503 (w), 1415 (m), 1340 (s), 1328 (s), 1303 (m), 1253 (s), 1231 (m), 1188 (m), 1183 (m), 1146 (m), 1140 (m), 1089 (s), 1046 (s), 1017 (m), 944 (m), 917 (m), 906 (m), 900 (m), 889 (m), 862 (m), 849 (m), 830 (s), 803 (s), 768 (m), 748 (s), 737 (s), 723 (s), 690 (vs).

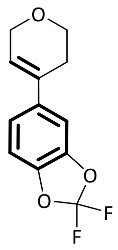

5-(3,6-Dihydro-2*H*-pyran-4-yl)-2,2-difluorobenzo[*d*][1,3]dioxole (4k)

Using potassium (3,6-dihydro-2*H*-pyran-4-yl)trifluoroborate **1k** and (2,2-difluorobenzo[*d*][1,3]dioxol-5-yl)magnesium bromide **A17** according to general procedure **D**, provided **4k** (0.12 mmol, 27 mg, 29%) as colourless oil.

$\boldsymbol{R_f}$ = 0.30 (hexane/EtOAc 98:2, UV, PAA). **^{1}H NMR** (400 MHz, $CDCl_3$) δ 7.11-7.06 (m, 2H), 7.03-6.99 (m, 1H), 6.06 (tt, *J* = 3.0, 1.6 Hz, 1H), 4.31 (q, *J* = 2.8 Hz, 2H), 3.93 (t, *J* = 5.4 Hz, 2H), 2.47 ppm (ttd, *J* = 5.5, 2.7, 1.6 Hz, 2H). **^{13}C NMR** (101 MHz, $CDCl_3$) δ 143.63 (d, *J* = 120.1 Hz), 143.0, 137.1, 132.8, 131.8 (t, *J* = 255.1 Hz), 123.3, 120.1, 109.3, 106.3, 65.9, 64.5, 27.6 ppm. **LRMS** (DEP/EI-Orbitrap): *m/z* (%): 240.0 (70), 222.0 (20), 196.9 (25), 158.0 (50). **HRMS** (EI-Orbitrap): m/z: [M^+] Calcd. for $C_{12}H_{10}F_2O_3^+$: 240.0598; found: 240.0592. **IR** (Diamond-ATR, neat) $\tilde{\nu}_{max}$ (cm^{-1}): 3248 (w), 3099 (w), 2961 (w), 2923 (w), 2853 (w), 1597 (m), 1565 (m), 1502 (m), 1451 (w), 1421 (m), 1346 (s), 1328 (m), 1303 (m), 1261 (s), 1238 (vs), 1183 (s), 1145 (s), 1088 (s), 1046 (s), 1035 (s), 944 (m), 916 (m), 908 (m), 889 (m), 862 (m), 849 (m), 830 (s), 810 (s), 803 (s), 767 (m), 748 (s), 737 (m), 723 (s), 690 (vs).

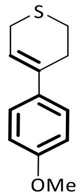

4-(4-Methoxyphenyl)-3,6-dihydro-2*H*-thiopyran (4l)

Using potassium (3,6-dihydro-2*H*-thiopyran-4-yl)trifluoroborate **1l** and (4-methoxyphenyl)magnesium bromide **A11** according to general procedure **D**, provided **4l** (0.31 mmol, 63 mg, 77%) as colourless oil. Yield was calculated by quantitative NMR-analysis since the hydrolysis of the (4-methoxyphenyl)magnesium bromide (= anisol) was not separable from the olefinic product via column chromatography. Impurity signals in the NMR-spectra were assigned.

$\boldsymbol{R_f}$ = 0.5 (hexane/EtOAc 98:2, UV, PAA). **^{1}H NMR** (400 MHz, $CDCl_3$) δ 7.30-7.25 (m, 2H), 6.89-6.84 (m, 2H), 6.12-6.08 (m, 1H), 3.81 (s, 3H), 3.33 (dt, *J* = 4.5, 2.3 Hz, 2H), 2.88 (t, *J* = 5.8 Hz, 2H), 2.70-2.64 ppm (m, 2H). **^{13}C NMR** (101 MHz, $CDCl_3$) δ 159.0, 137.7, 135.6, 126.7, 120.3, 113.8, 55.4, 28.8, 26.4, 25.3 ppm. **LRMS** (DEP/EI-Orbitrap): *m/z* (%): 206.1 (100), 191.0 (5), 177.0 (60), 147.1 (75). **IR** (Diamond-

ATR, neat) $\tilde{\nu}_{max}$ (cm^{-1}): 3248 (w), 2999 (w), 2934 (w), 2835 (w), 1601 (w), 1578 (w), 1511 (vs), 1493 (m), 1463 (m), 1453 (m), 1443 (m), 1414 (m), 1340 (w), 1322 (w), 1309 (w), 1252 (vs), 1229 (vs), 1179 (m), 1134 (s), 1086 (w), 1071 (vw), 1026 (s), 926 (w), 895 (w), 862 (m), 814 (m), 800 (w), 779 (s), 765 (m), 738 (w), 698 (m), 656 (w).

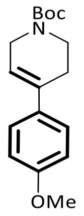

***tert*-Butyl 4-(4-methoxyphenyl)-3,6-dihydropyridine-1(2*H*)-carboxylate (4m)**

Using potassium (1-(*tert*-butoxycarbonyl)-1,2,3,6-tetrahydropyridin-4-yl)trifluoroborate **1m** and (4-methoxyphenyl)magnesium bromide **A11** according to general procedure **D**, provided **4m** (0.21 mmol, 60 mg, 52%) as colourless oil.

^{1}H NMR (400 MHz, $CDCl_3$) δ 7.35-7.29 (m, 2H), 6.93-6.83 (m, 2H), 5.94 (s, 1H), 4.05 (s, 2H), 3.81 (s, 3H), 3.63 (t, *J* = 5.7 Hz, 2H), 2.50 (s, 2H), 1.49 ppm (s, 9H). **^{13}C NMR** (101 MHz, $CDCl_3$) δ 159.0, 133.4, 131.8, 127.8, 126.1, 113.9, 79.8, 55.4, 43.6, 40.7, 39.2, 28.6 ppm. **LRMS** (DEP/EI-Orbitrap): *m/z* (%): 232.1 (100), 202.1 (15), 188.1 (34), 160.0 (16). Analytical data in accordance to literature.[30]

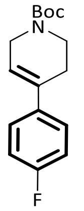

***tert*-Butyl 4-(4-fluorophenyl)-3,6-dihydropyridine-1(2*H*)-carboxylate (4n)**

Using potassium (1-(*tert*-butoxycarbonyl)-1,2,3,6-tetrahydropyridin-4-yl)trifluoroborate **1m** and (4-fluorophenyl)magnesium bromide **A1** according to general procedure **D**, provided **4n** (0.22 mmol, 62 mg, 56%) as colourless oil.

^{1}H NMR (400 MHz, $CDCl_3$) δ 7.37-7.29 (m, 2H), 7.06-6.98 (m, 2H), 5.97 (s, 1H), 4.09-4.02 (m, 2H), 3.63 (t, *J* = 5.7 Hz, 2H), 2.49 (s, 2H), 1.49 ppm (s, 9H). **^{13}C NMR** (101 MHz, $CDCl_3$) δ 162.23 (d, *J* = 246.2 Hz), 155.0, 136.9, 134.8, 126.60 (d, *J* = 7.9 Hz), 115.35 (d, *J* = 21.3 Hz), 113.0, 79.9, 43.8, 39.9, 28.6, 27.7 ppm. **LRMS** (DEP/EI-Orbitrap): *m/z* (%): 220.1 (100), 204.1 (15), 177.1 (70). Analytical data in accordance to literature.[31]

---

30 A. Music; C. Hoarau; N. Hilgert; F. Zischka; D. Dorian, *Angew. Chem. Int. Ed.* **2019**, *58*, 1188.
31 D.J. Wustrow; L. D. Wise, *Synthesis* **1991**, *11*, 993.

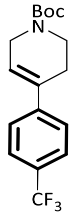

tert-Butyl 4-(4-(trifluoromethyl)phenyl)-3,6-dihydropyridine-1(2_H_)-carboxylate (4o)

Using potassium (1-(_tert_-butoxycarbonyl)-1,2,3,6-tetrahydropyridin-4-yl)trifluoroborate **1m** and (4-(trifluoromethyl)phenyl)magnesium bromide **A18** according to general procedure **D**, provided **4o** (0.17 mmol, 56 mg, 43%) as colourless oil.

^{1}H NMR (400 MHz, $CDCl_3$) δ 7.58 (d, _J_ = 8.7 Hz, 2H), 7.46 (d, _J_ = 8.1 Hz, 2H), 6.12 (s, 1H), 4.15-4.00 (m, 2H), 3.65 (t, _J_ = 5.7 Hz, 2H), 2.53 (s, 2H), 1.49 ppm (s, 9H). **^{13}C NMR** (101 MHz, $CDCl_3$) δ 154.9, 144.2, 134.7, 129.27 (q, _J_ = 32.6 Hz), 126.77 (q, _J_ = 129.9 Hz), 125.51 (q, _J_ = 3.8 Hz), 125.3, 123.0, 80.0, 44.0, 39.8, 28.6, 27.4 ppm. **LRMS** (DEP/EI-Orbitrap): _m/z_ (%): 271.1 (100), 254.1 (15), 227.1 (90). Analytical data in accordance to literature.[32]

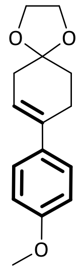

8-(4-Methoxyphenyl)-1,4-dioxaspiro[4.5]dec-7-ene (4p)

Using potassium trifluoro(1,4-dioxaspiro[4.5]dec-7-en-8-yl)borate **1n** and (4-methoxyphenyl)magnesium bromide **A11** according to general procedure **D**, provided **4p** (0.35 mmol, 86 mg, 87%) as colourless oil.

^{1}H NMR (400 MHz, $CDCl_3$) δ 7.35-7.31 (m, 2H), 6.88-6.81 (m, 2H), 5.90 (td, _J_ = 3.9, 2.0 Hz, 1H), 4.02 (s, 4H), 3.80 (s, 3H), 2.66-2.60 (m, 2H), 2.51-2.42 (m, 2H), 1.92 ppm (t, _J_ = 6.5 Hz, 2H). **^{13}C NMR** (101 MHz, $CDCl_3$) δ 158.7, 135.7, 134.2, 126.4, 120.0, 113.6, 108.0, 64.6, 55.4, 36.2, 31.5, 27.0 ppm. **LRMS** (DEP/EI-Orbitrap): _m/z_ (%): 246.1 (40), 231.0 (2). 160.1 (100). Analytical data in accordance to literature.[33]

[32] Merck & Co., US6303593, **2001**, B1.
[33] A. J. Pearson; I. C. Richards; D. V. Gardner, _J. Org. Chem._ **1984**, _49_, 3887.

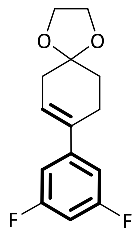

8-(3,5-Difluorophenyl)-1,4-dioxaspiro[4.5]dec-7-ene (4q)

Using potassium trifluoro(1,4-dioxaspiro[4.5]dec-7-en-8-yl)borate **1n** and (3,5-difluorophenyl)magnesium bromide **A8** according to general procedure **D**, provided **4q** (0.16 mmol, 40 mg, 40%) as colourless oil.

***R*f** = 0.10 (hexane/EtOAc 98:2, UV, PAA). **^{1}H NMR** (400 MHz, $CDCl_3$) δ 6.95-6.85 (m, 2H), 6.66 (tt, *J* = 8.8, 2.3 Hz, 1H), 6.05 (tt, *J* = 4.0, 1.6 Hz, 1H), 4.02 (s, 4H), 2.63-2.56 (m, 2H), 2.49-2.44 (m, 2H), 1.91 ppm (t, *J* = 6.6 Hz, 2H). **^{13}C NMR** (101 MHz, $CDCl_3$) δ 163.1 (dd, *J* = 246.8, 13.3 Hz), 144.9 (t, *J* = 9.2 Hz), 134.6 (t, *J* = 2.6 Hz), 124.0, 108.3-107.9 (m), 107.6, 102.1 (t, *J* = 25.6 Hz), 64.7, 36.2, 31.3, 26.6 ppm. **LRMS** (DEP/EI-Orbitrap): *m/z* (%): 252.1 (35), 237.0 (5) 164.0 (15), 151.0 (15), 86.0 (100). **HRMS** (EI-Orbitrap): m/z: [M$^+$] Calcd. for $C_{14}H_{14}F_2O_2^+$: 252.0962; found: 252.0956. **IR** (Diamond-ATR, neat) $\tilde{\nu}_{max}$ (cm^{-1}): 2942 (vs), 2929 (vs), 2892 (s), 2866 (vs), 1502 (vs), 1463 (s), 1254 (m), 1202 (s), 1148 (m), 1096 (s), 1059 (m), 1037 (s), 1017 (s), 1004 (s), 993 (s), 981 (s), 942 (m), 931 (m), 919 (m), 884 (s), 863 (m), 830 (s), 810 (m), 804 (m), 775 (m), 709 (m), 677 (s), 668 (s), 662 (s).

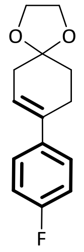

8-(4-Fluorophenyl)-1,4-dioxaspiro[4.5]dec-7-ene (4r)

Using potassium trifluoro(1,4-dioxaspiro[4.5]dec-7-en-8-yl)borate **1n** and (4-fluorophenyl)magnesium bromide **A1** according to general procedure **D**, provided **4r** (0.34 mmol, 80 mg, 86%) as colourless oil.

***R*f** = 0.15 (hexane/EtOAc 98:2, UV, PAA). **^{1}H NMR** (400 MHz, $CDCl_3$) δ 7.38-7.31 (m, 2H), 7.02-6.95 (m, 2H), 5.93 (tt, *J* = 3.9, 1.6 Hz, 1H), 4.02 (s, 4H), 2.63 (tq, *J* = 6.4, 2.1 Hz, 2H), 2.48-2.44 (m, 2H), 1.92 ppm (t, *J* = 6.5 Hz, 2H). **^{13}C NMR** (101 MHz, $CDCl_3$) δ 162.1 (d, *J* = 245.4 Hz), 137.7 (d, *J* = 3.2 Hz), 135.5, 126.8 (d, *J* = 7.8 Hz), 121.6 (d, *J* = 1.4 Hz), 115.1 (d, *J* = 21.2 Hz), 107.8, 64.6, 36.2, 31.4, 27.1 ppm. **LRMS** (DEP/EI-Orbitrap): *m/z* (%): 234.1 (25), 219.1 (5), 146.0 (20), 133.0 (20), 86.0 (100). **HRMS** (EI-Orbitrap): m/z: [M$^+$] Calcd. for $C_{14}H_{15}FO_2^+$: 234.1056; found: 234.1049. **IR** (Diamond-ATR, neat) $\tilde{\nu}_{max}$ (cm^{-1}):

3406 (w), 2925 (w), 1709 (s), 1599 (m), 1509 (m), 1500 (m), 1412 (m), 1360 (s), 1277 (m), 1261 (m), 1221 (vs), 1158 (s), 1092 (s), 1031 (s), 1014 (s), 946 (m), 931 (m), 900 (m), 881 (m), 828 (s), 811 (s), 748 (m), 682 (m), 668 (m).

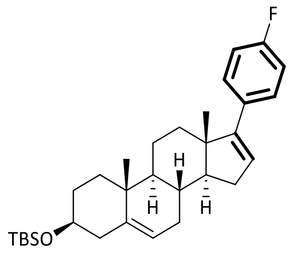

***tert*-Butyl(((*3S*,*8R*,*9S*,*10R*,*13S*,*14S*)-17-(4-fluorophenyl)-10,13-dimethyl-2,3,4,7,8,9,10,11,12,13,14,15-dodecahydro-1*H*-cyclopenta[*a*]phenanthren-3-yl)oxy)dimethylsilane (5a)**

Using potassium ((*3S*,*8S*,*9S*,*10R*,*13S*,*14S*)-3-((*tert*-butyldimethylsilyl)oxy)-10,13-dimethyl-2,3,4,7,8,9,10,11,12,13,14,15-dodecahydro-1*H*-cyclopenta[*a*]phenanthren-17-yl)trifluoroborate **1o** and (4-fluorophenyl)magnesium bromide **A1** according to general procedure **D**, provided **5a** (0.25 mmol, 121mg, 63%) as colourless oil.

$\boldsymbol{R}_{\mathbf{f}}$ = 0.2 (hexane/EtOAc 98:2, UV, PAA, $KMnO_4$). **^{1}H NMR** (400 MHz, $CDCl_3$) δ 7.32 (dd, *J* = 8.7, 5.5 Hz, 2H), 7.01-6.95 (m, 2H), 5.85 (dd, *J* = 3.4, 1.8 Hz, 1H), 5.38-5.33 (m, 1H), 3.50 (tt, *J* = 11.0, 4.7 Hz, 1H), 2.34-2.15 (m, 3H), 2.09-1.96 (m, 3H), 1.85-1.41 (m, 10H), 1.33-1.20 (m, 1H), 1.06 (s, 3H), 1.03 (s, 3H), 0.90 (s, 9H), 0.07 ppm (s, 6H). **^{13}C NMR** (101 MHz, $CDCl_3$) δ 161.9 (d, *J* = 245.4 Hz), 153.8, 141.9, 133.4 (d, *J* = 3.5 Hz), 128.2 (d, *J* = 7.7 Hz), 127.1, 120.9, 114.9 (d, *J* = 21.0 Hz), 72.6, 57.7, 50.5, 50.4, 47.2, 42.9, 37.3, 36.8, 35.4, 32.1, 31.6, 30.5, 26.0, 20.9, 19.4, 18.3, 16.6, −4.4 ppm. **LRMS** (DEP/EI-Orbitrap): *m/z* (%): 423.3 (50), 348.2 (5), 207.0 (100). **HRMS** (EI-Orbitrap): m/z: [M^+] Calcd. for $C_{31}H_{45}FOSi^+$: 480.3224; found: 480.3211. **IR** (Diamond-ATR, neat) $\tilde{\nu}_{max}$ (cm^{-1}): 2929 (m), 2900 (m), 2855 (m), 1714 (w), 1600 (w), 1507 (m), 1471 (w), 1462 (m), 1437 (w), 1408 (w), 1380 (w), 1371 (w), 1361 (w), 1294 (w), 1250 (m), 1227 (m), 1159 (m), 1089 (s), 1006 (m), 959 (w), 938 (w), 925 (w), 888 (m), 870 (m), 835 (vs), 806 (s), 774 (s), 736 (w), 718 (w), 668 (m).

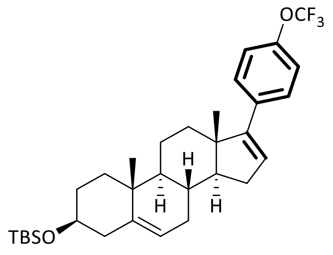

***tert*-Butyl(((*3S*,*8R*,*9S*,*10R*,*13S*,*14S*)-10,13-dimethyl-17-(4-(trifluoromethoxy)phenyl)-2,3,4,7,8,9,10,11,12,13,14,15-dodecahydro-1*H*-cyclopenta[*a*]phenanthren-3-yl)oxy)dimethylsilane (5b)**

Using potassium ((*3S*,*8S*,*9S*,*10R*,*13S*,*14S*)-3-((*tert*-butyldimethylsilyl)oxy)-10,13-dimethyl-2,3,4,7,8,9,10,11,12,13,14,15-dodecahydro-1*H*-cyclopenta[*a*]phenanthren-17-yl)trifluoroborate **1o** and (4-fluorophenyl)magnesium bromide **A9** according to general procedure **D**, provided **5b** (0.28 mmol, 153 mg, 70%) as colourless oil.

$\boldsymbol{R}_\mathrm{f}$ = 0.3 (hexane/EtOAc 98:2, UV, PAA, $KMnO_4$). **^{1}H NMR** (400 MHz, $CDCl_3$) δ 7.42-7.35 (m, 2H), 7.16-7.09 (m, 2H), 5.92 (dd, *J* = 3.3, 1.8 Hz, 1H), 5.38-5.33 (m, 1H), 3.49 (tt, *J* = 11.0, 4.7 Hz, 1H), 2.35-2.16 (m, 3H), 2.09-1.97 (m, 3H), 1.87-1.41 (m, 10H), 1.30-1.21 (m, 1H), 1.06 (s, 3H), 1.04 (s, 3H), 0.89 (s, 9H), 0.06 (s, 6H). **^{13}C NMR** (101 MHz, $CDCl_3$) δ 153.7, 148.1, 142.0, 136.2, 128.4, 128.0, 121.0, 120.8, 120.6 (q, *J* = 256.7 Hz), 77.2, 72.7, 57.8, 50.6, 47.4, 43.0, 37.4, 36.9, 35.5, 32.2, 31.8, 31.7, 30.6, 26.1, 21.0, 19.5, 18.4, 16.7, -4.4 ppm. **LRMS** (DEP/EI-Orbitrap): *m/z* (%): 489.2 (100), 413.1 (5), 329.1 (10). **HRMS** (EI-Orbitrap): m/z: [M$^+$] Calcd. for $C_{32}H_{45}F_3O_2Si^+$: 546.3141; found: 546.3138. **IR** (Diamond-ATR, neat) $\tilde{\nu}_{max}$ (cm^{-1}): 2959 (w), 2936 (w), 2930 (w), 2897 (w), 2857 (w), 1749 (w), 1712 (s), 1602 (w), 1507 (w), 1470 (w), 1458 (w), 1437 (w), 1428 (w), 1382 (m), 1361 (s), 1257 (s), 1220 (vs), 1166 (m), 1085 (s), 1062 (m), 1048 (m), 1019 (m), 1003 (m), 957 (m), 937 (m), 922 (m), 911 (m), 888 (s), 869 (s), 838 (s), 815 (m), 802 (m), 771 (m), 734 (m), 672 (m).

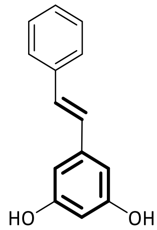

(*E*)-5-Styrylbenzene-1,3-diol (pinosylvin) (5c)

Using potassium (*E*)-trifluoro(styryl)borate **1a** and (3,5-dimethoxyphenyl)magnesium bromide **A3** according to general procedure **D**, provided (*E*)-1,3-dimethoxy-5-styrylbenzene. For removal of the methyl groups, the crude compound, after electrochemical oxidation, was dissolved in CH_2Cl_2 (4 mL), cooled down to -20 °C and treated with a solution of BBr_3 (1.6 mmol, 4 equiv.) dissolved in 1 mL CH_2Cl_2.

The reaction was let warm to room temperature. After completion, the reaction was treated with water and extracted with dichloromethane (3 × 10 mL) and washed with a solution of saturated aqueous NaCl (1 × 10 mL). The combined organic phases were dried over magnesium sulfate, filtered and concentrated in vacuo. The crude was purified by flash column chromatography with appropriate solvent mixture to provide **5c** (0.23 mmol, 62 mg, 57%, *E*/*Z* = 99:1) as colourless solid.

^{1}H NMR (400 MHz, $CDCl_3$) δ 7.51-7.46 (m, 2H), 7.39-7.33 (m, 2H), 7.29-7.26 (m, 1H), 7.05 (d, *J* = 16.3 Hz, 1H), 6.96 (d, *J* = 16.3 Hz, 1H), 6.58 (d, *J* = 2.2 Hz, 2H), 6.28 (t, *J* = 2.2 Hz, 1H), 4.76 ppm (s, 2H). **^{13}C NMR** (101 MHz, $CDCl_3$) δ 156.9, 140.0, 136.8, 129.6, 128.7, 127.9, 127.1, 126.6, 126.3, 106.2, 102.2 ppm. **HRMS** (EI-Orbitrap): m/z: [M^+] Calcd. for $C_{14}H_{12}O_2^+$: 212.0837; found: 212.0830. Analytical data in accordance to literature.[34]

[34] J. Yang; C. Wang; Y. Sun; X. Man; Y. Li; F. Sun, *Chem. Commun.* **2019**, *13*, 1903.

3. NMR Spectra

Potassium (*Z*)-trifluoro(2-(6-methoxynaphthalen-2-yl)vinyl)borate (1g)

^{1}H NMR (400 MHz, MeCN-*d*$_3$) and **^{13}C NMR** (101 MHz, MeCN-*d*$_3$)

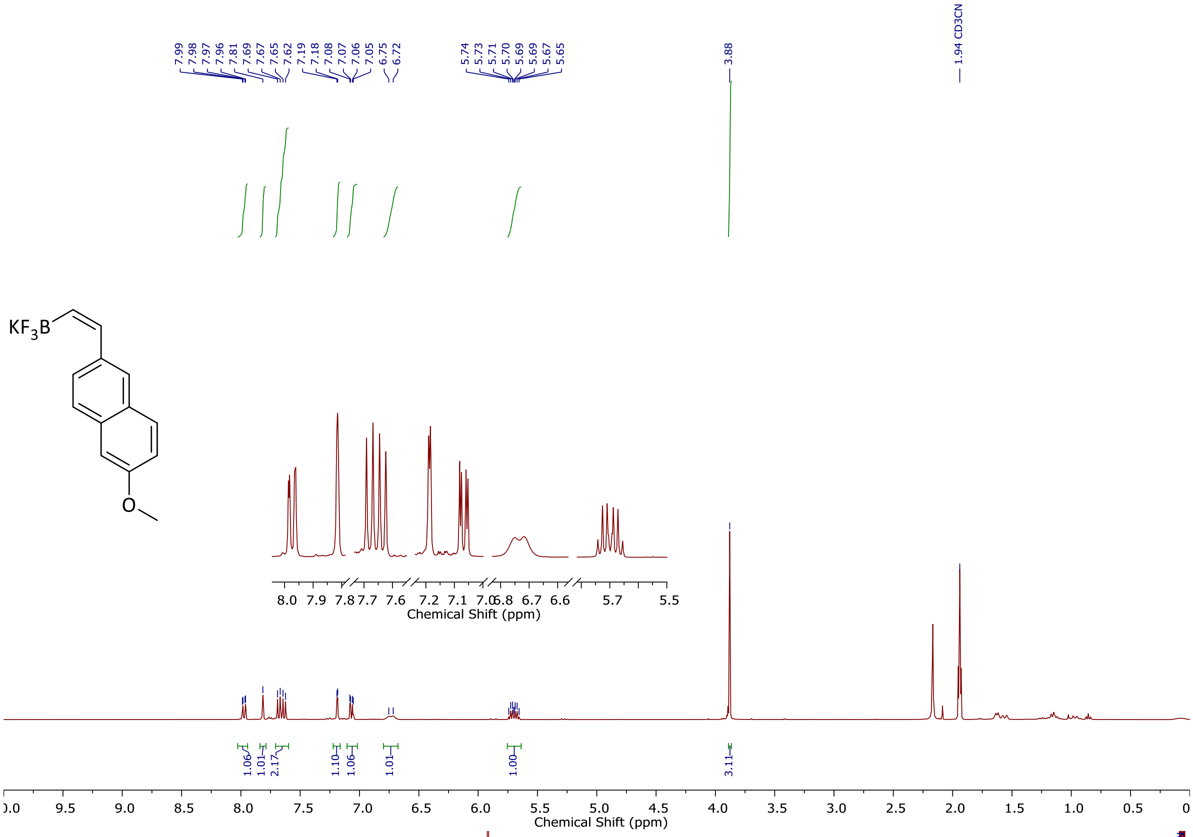

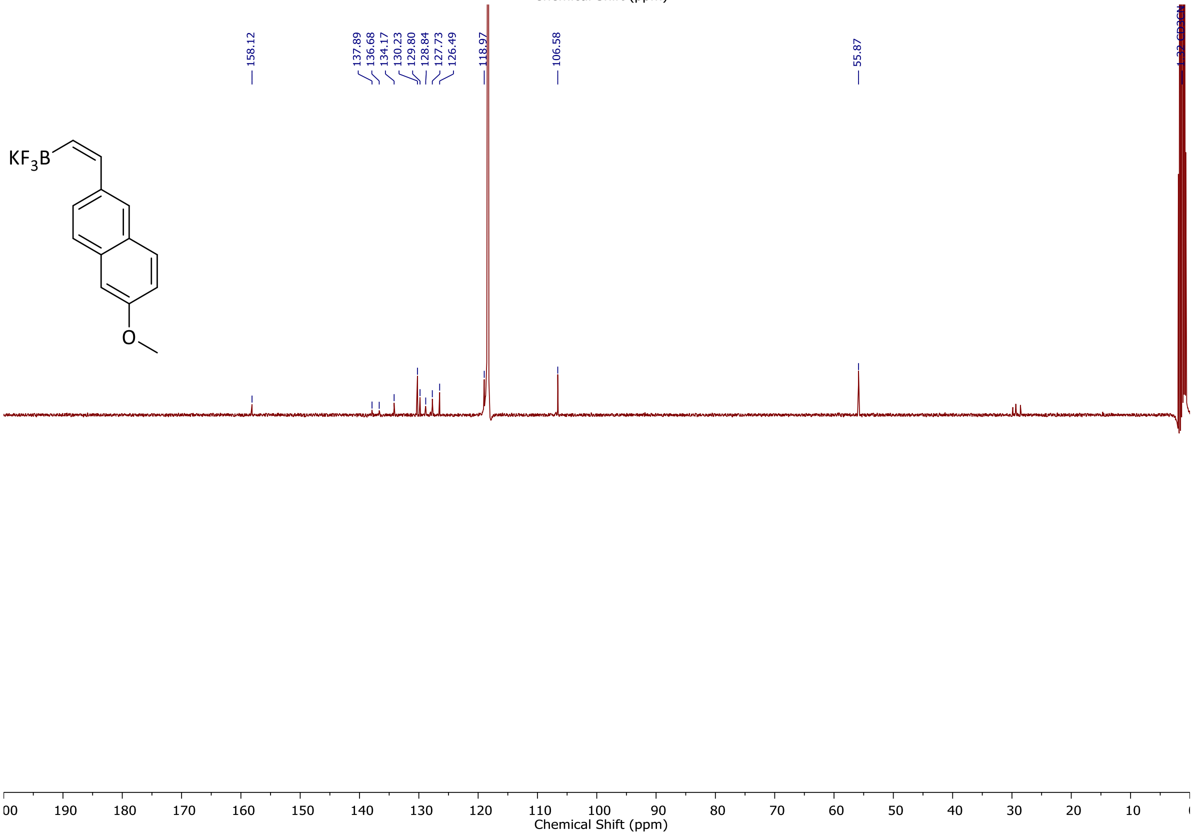

Potassium cyclopent-1-en-1-yltrifluoroborate (1i)

^{1}H NMR (400 MHz, MeCN-d_3) and **^{13}C NMR** (101 MHz, MeCN-d_3)

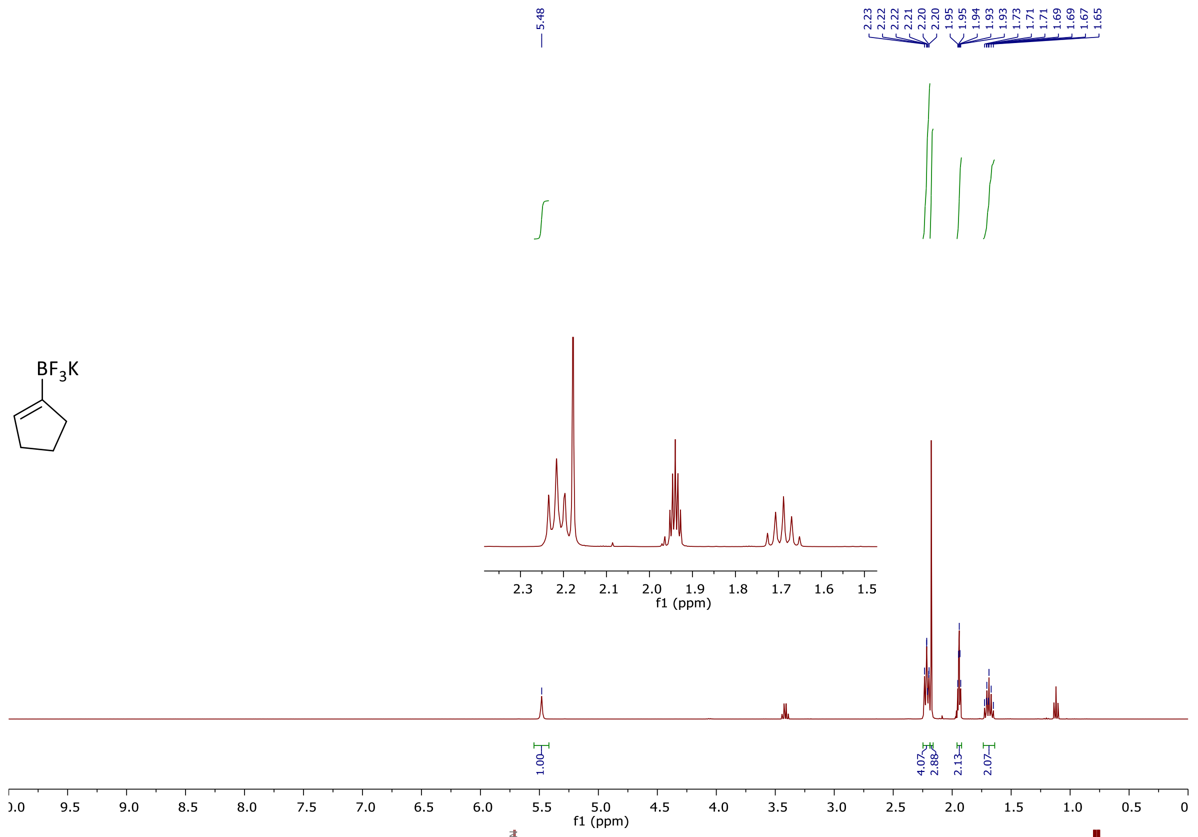

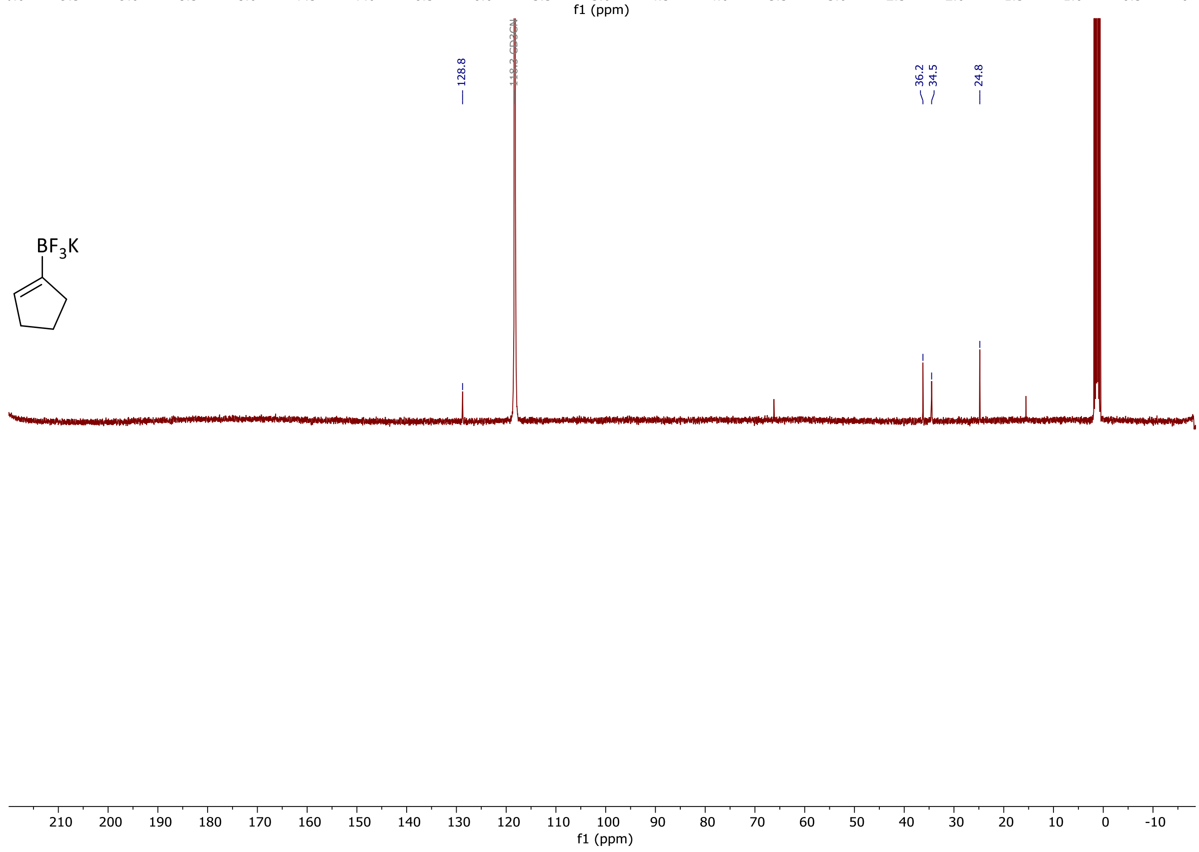

Potassium (3,6-dihydro-2H-pyran-4-yl)trifluoroborate (1k)

^{1}H NMR (400 MHz, MeCN-d_3) and **^{13}C NMR** (101 MHz, MeCN-d_3)

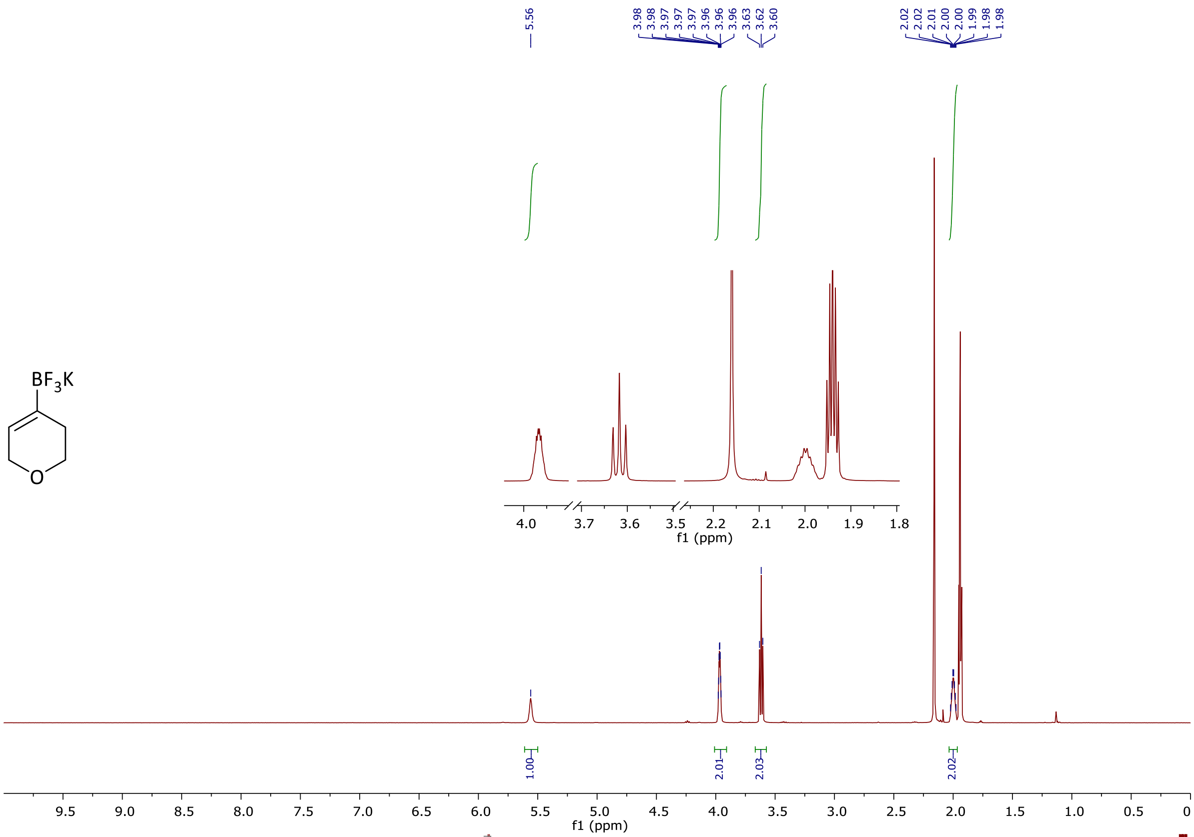

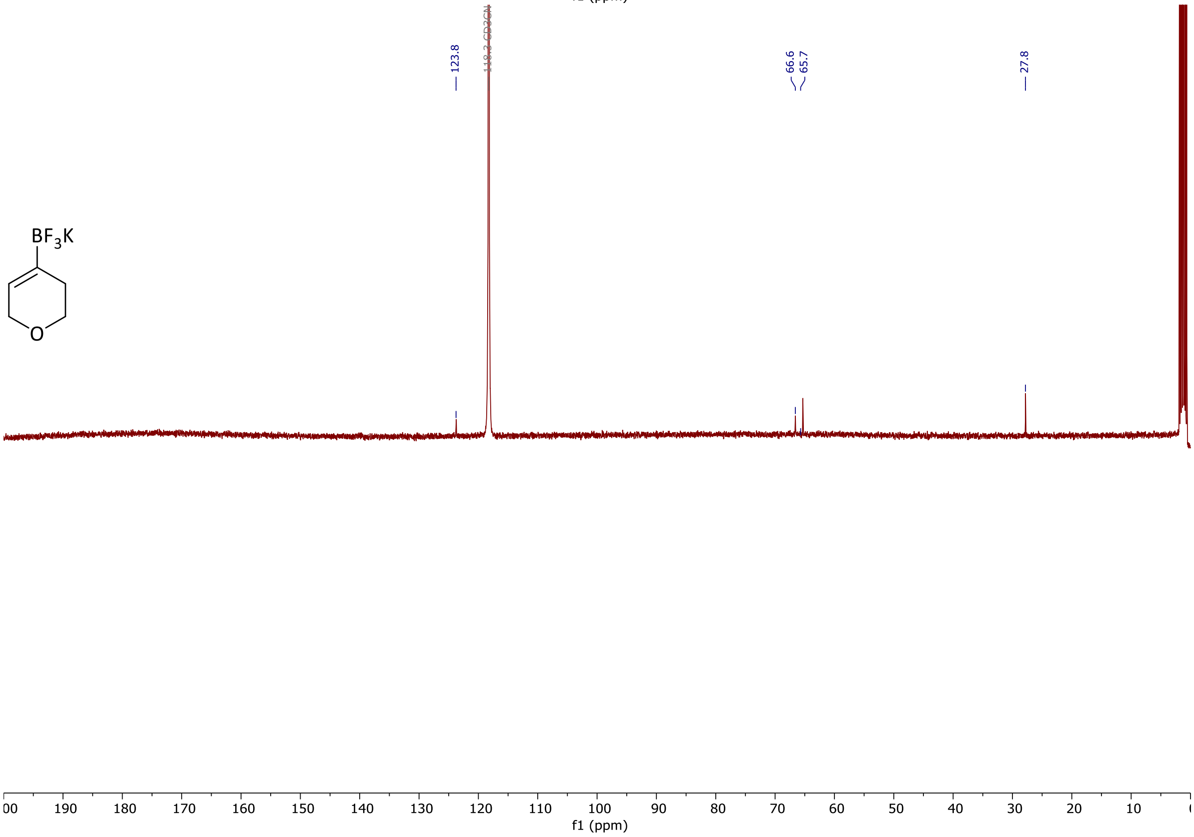

Potassium (3,6-dihydro-2H-thiopyran-4-yl)trifluoroborate (1l)

^{1}H NMR (400 MHz, MeCN-d_3) and **^{13}C NMR** (101 MHz, MeCN-d_3)

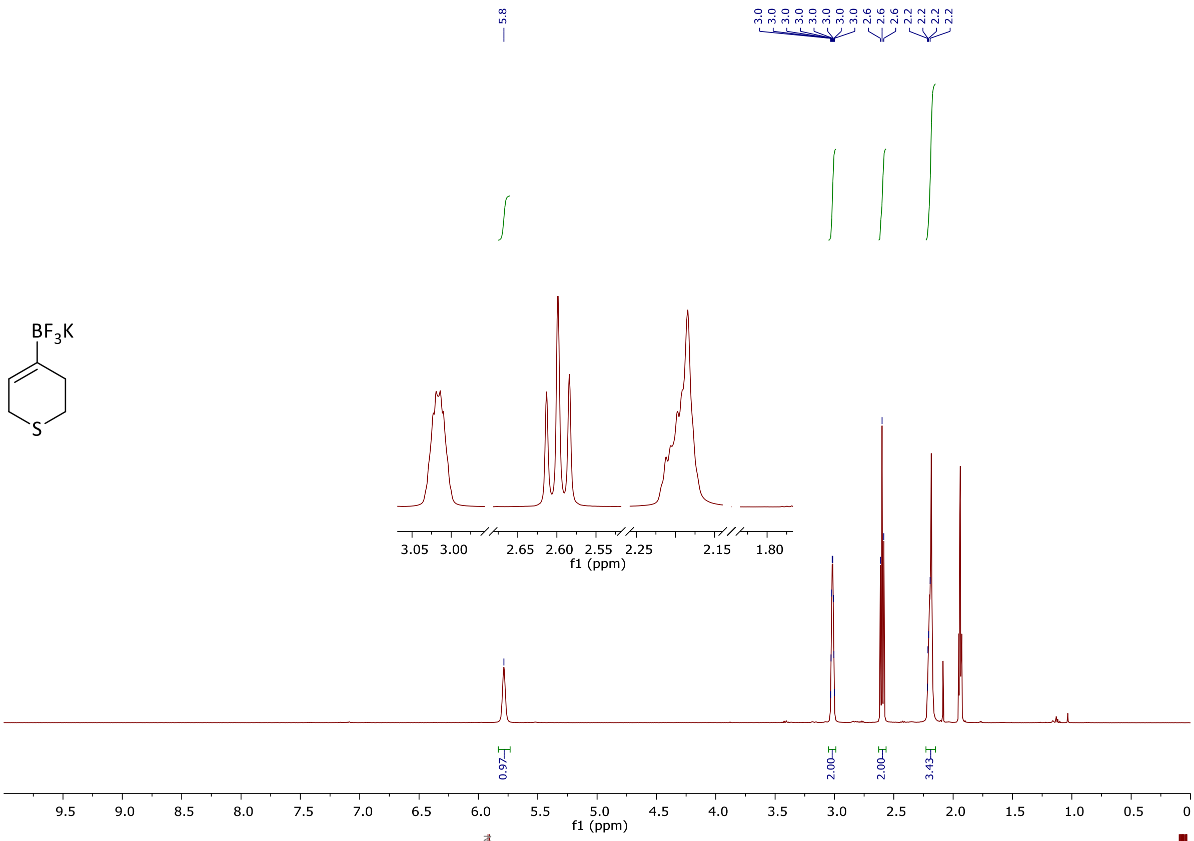

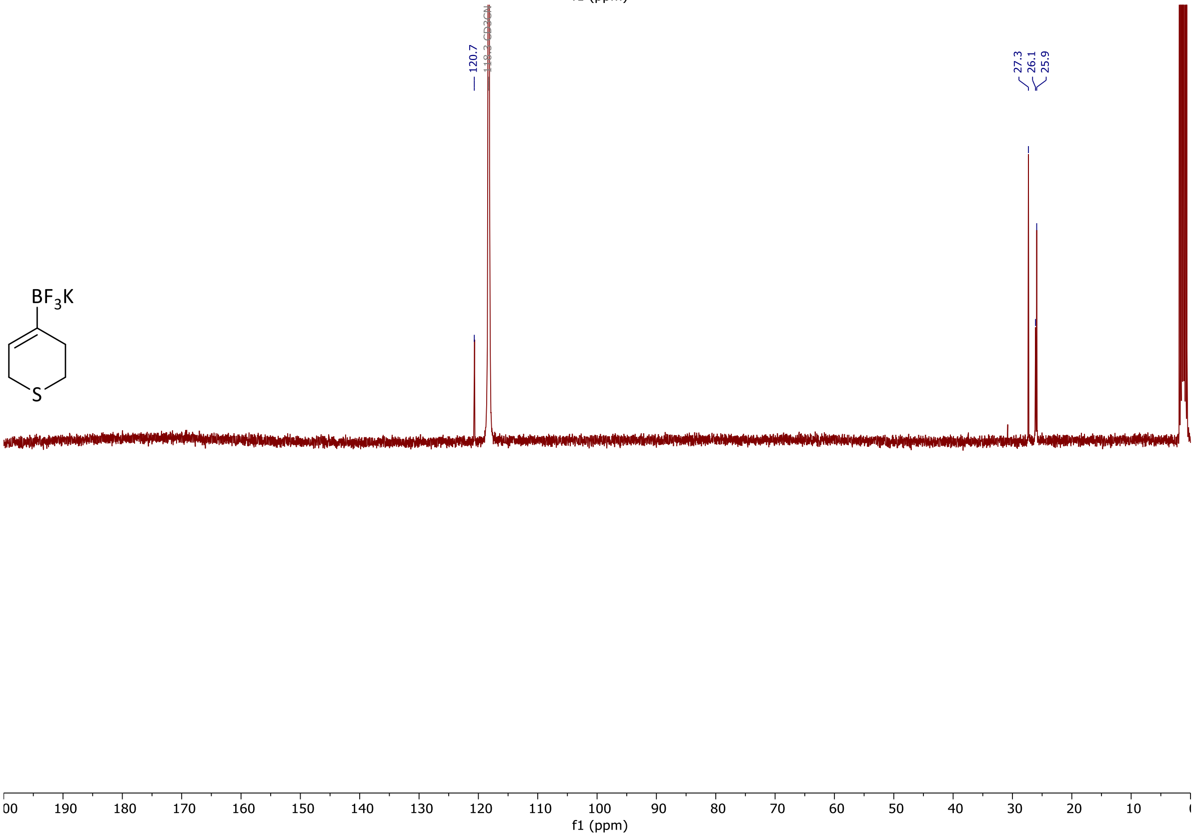

Potassium trifluoro(1,4-dioxaspiro[4.5]dec-7-en-8-yl)borate (1n)

^{1}H NMR (400 MHz, MeCN-d_3) and **^{13}C NMR** (101 MHz, MeCN-d_3)

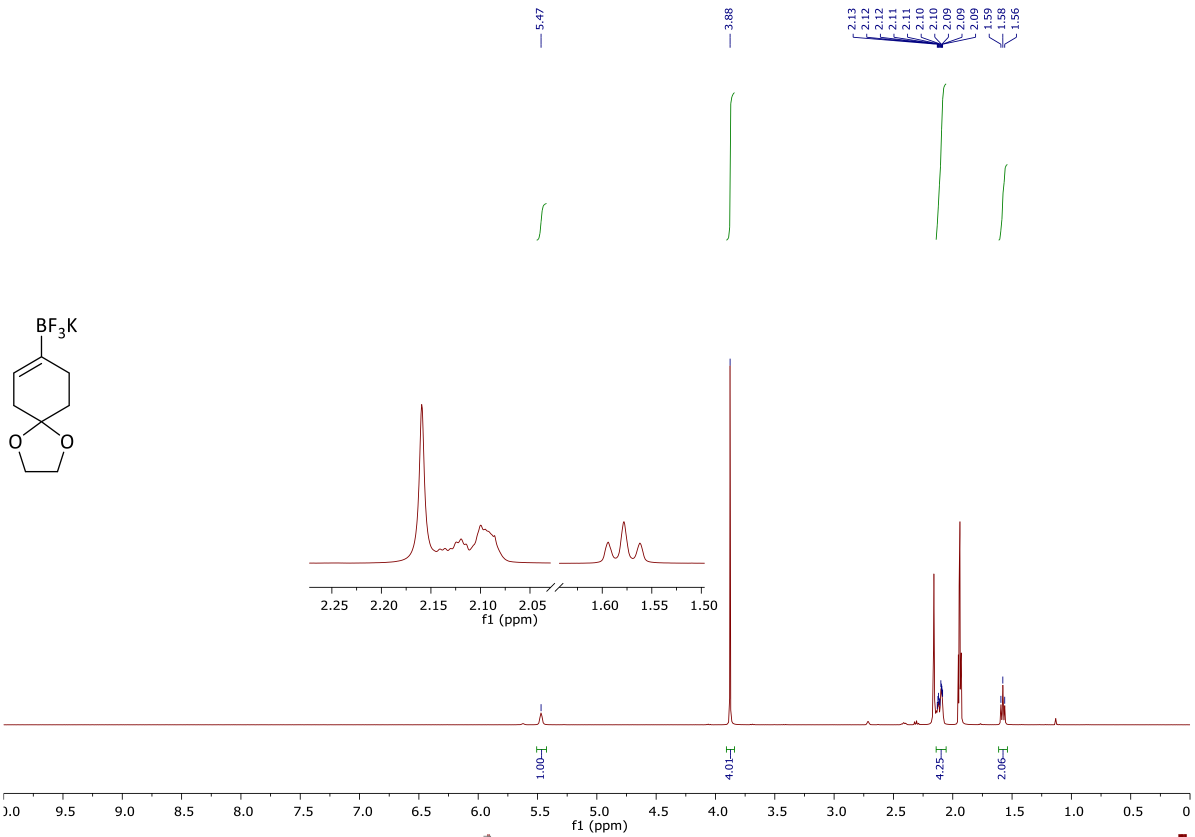

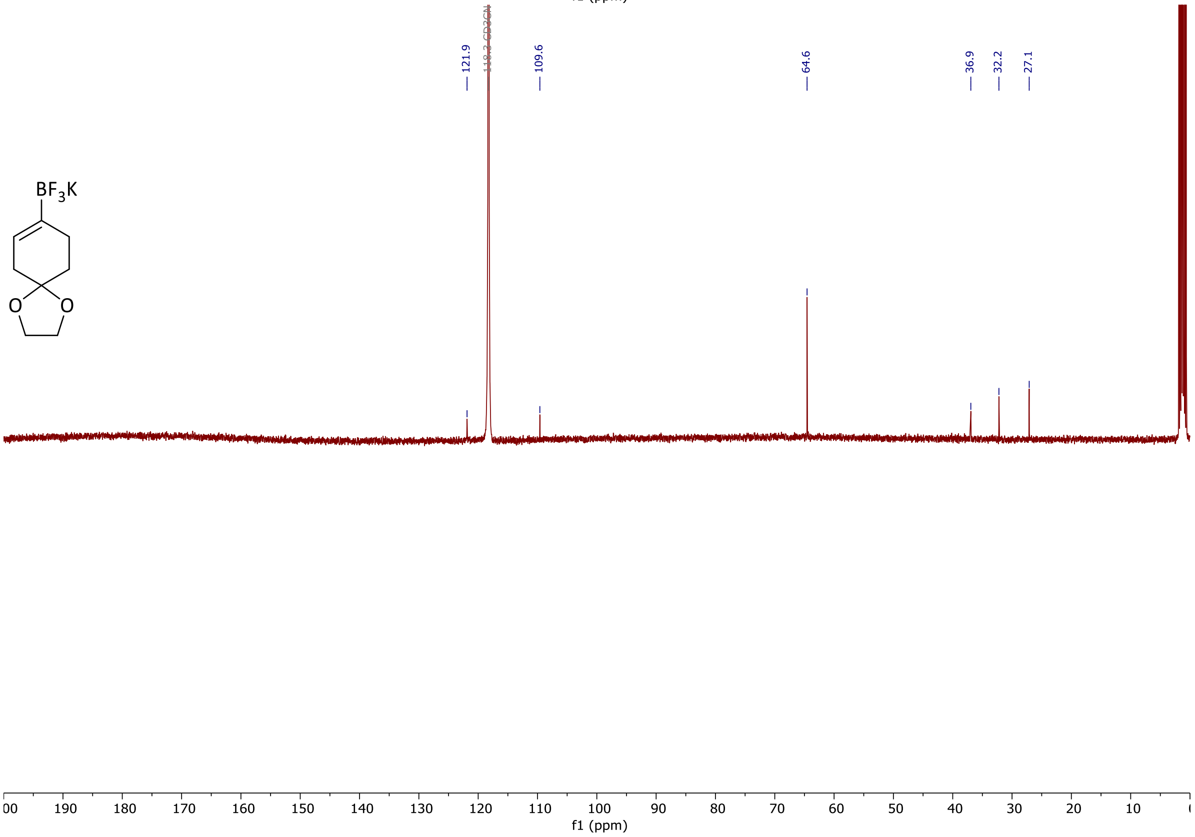

tert-butyl(((*3S*,*8S*,*9S*,*10R*,*13S*,*14S*)-10,13-dimethyl-17-(4,4,5,5-tetramethyl-1,3,2-dioxaborolan-2-yl)-2,3,4,7,8,9,10,11,12,13,14,15-dodecahydro-1*H*-cyclopenta[*a*]phenanthren-3-yl)oxy)dimethylsilane **(1o-SM)**

^{1}H NMR (400 MHz, $CDCl_3$) and **^{13}C NMR** (101 MHz, $CDCl_3$)

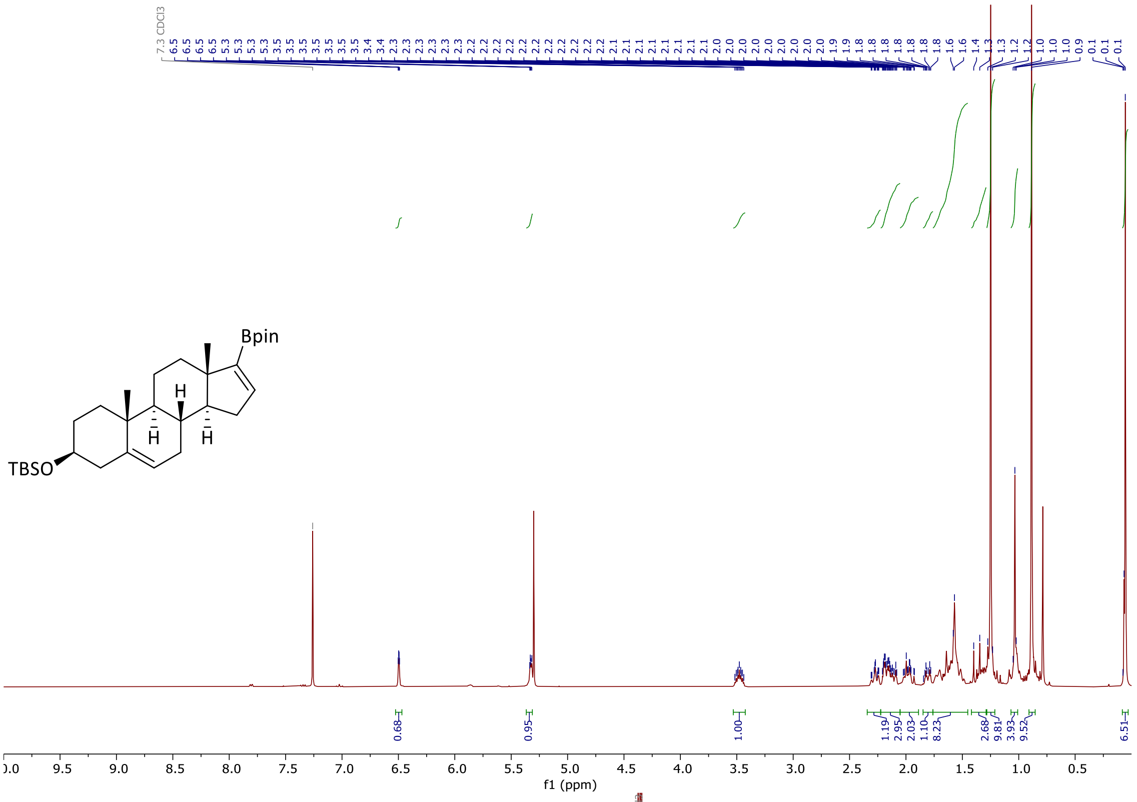

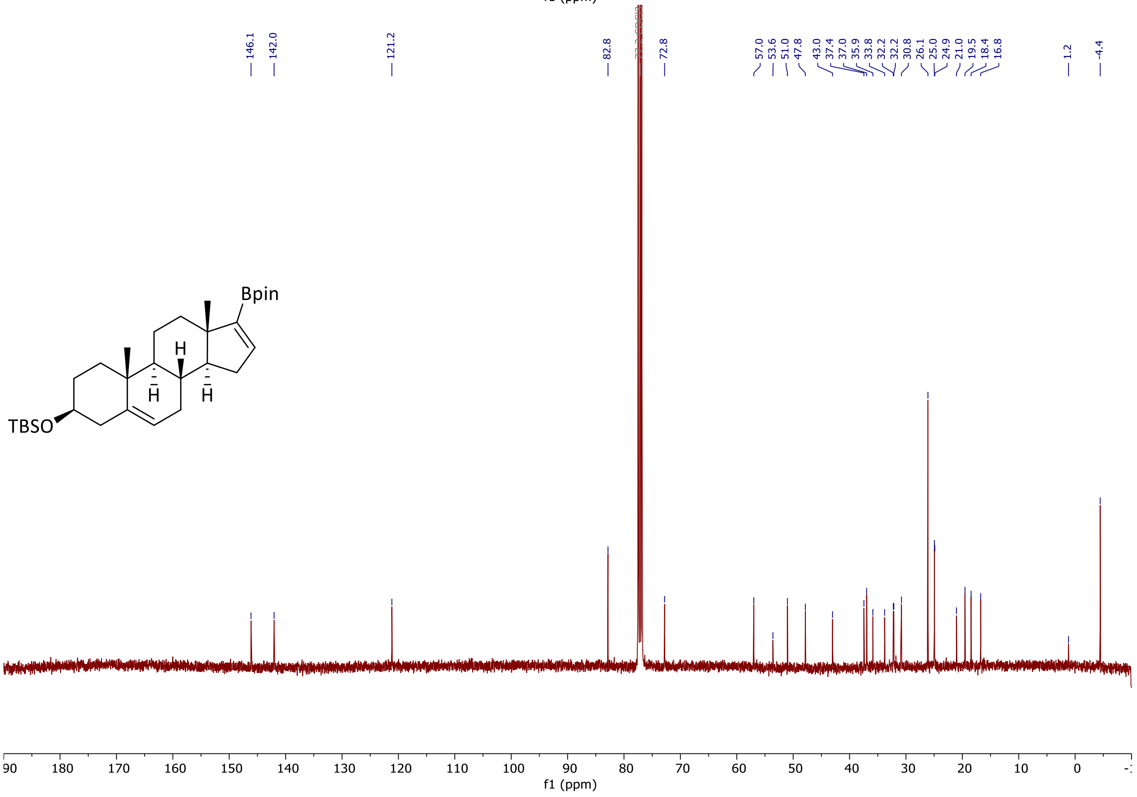

Potassium((*3S*,*9S*,*10R*,*13S*,*14S*)-3-((*tert*-butyldimethylsilyl)oxy)-10,13-dimethyl-2,3,4,7,8,9,10,11,12,13,14,15-dodecahydro-1*H*-cyclopenta[*a*]phenanthren-17-yl)trifluoroborate (1o)

^{1}H NMR (400 MHz, MeCN-d_3)

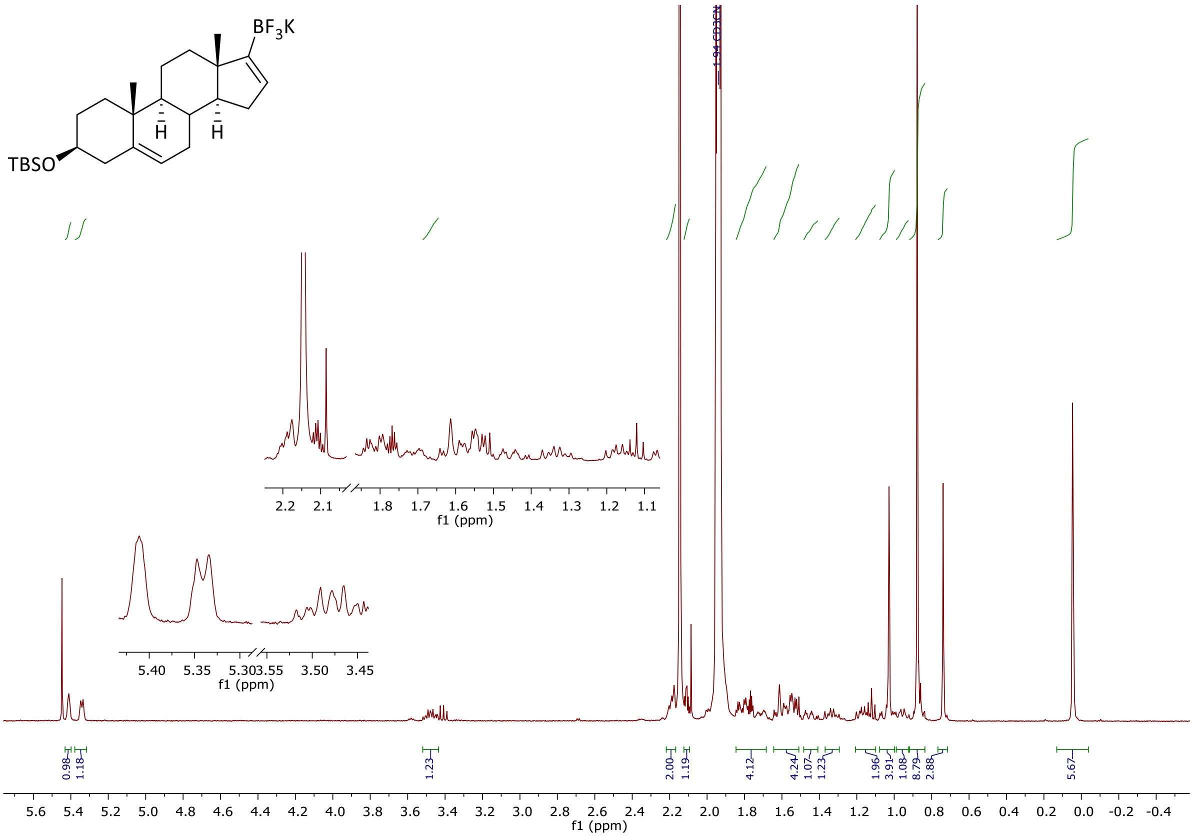

Potassium difluorobis(4-fluorophenyl) borate (1p)

^{1}H NMR (400 MHz, MeCN-d_3) and **^{13}C NMR** (101 MHz, MeCN-d_3)

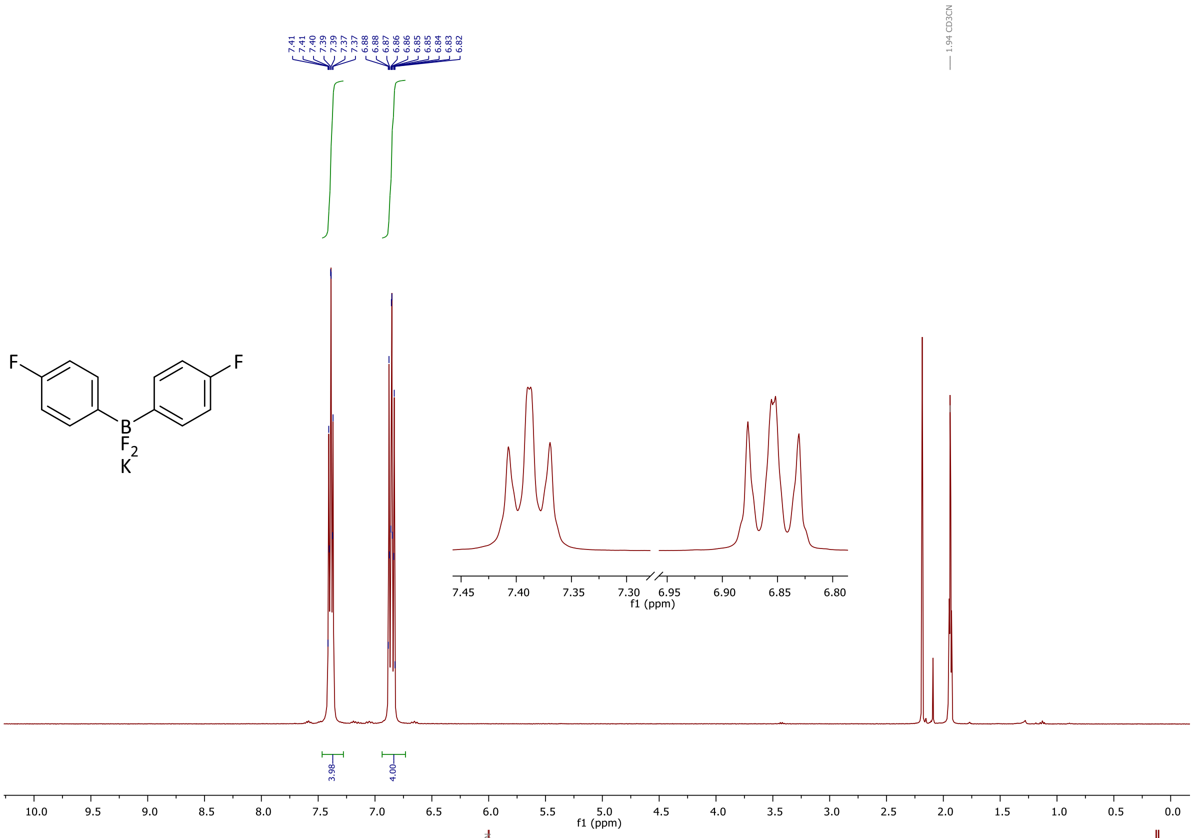

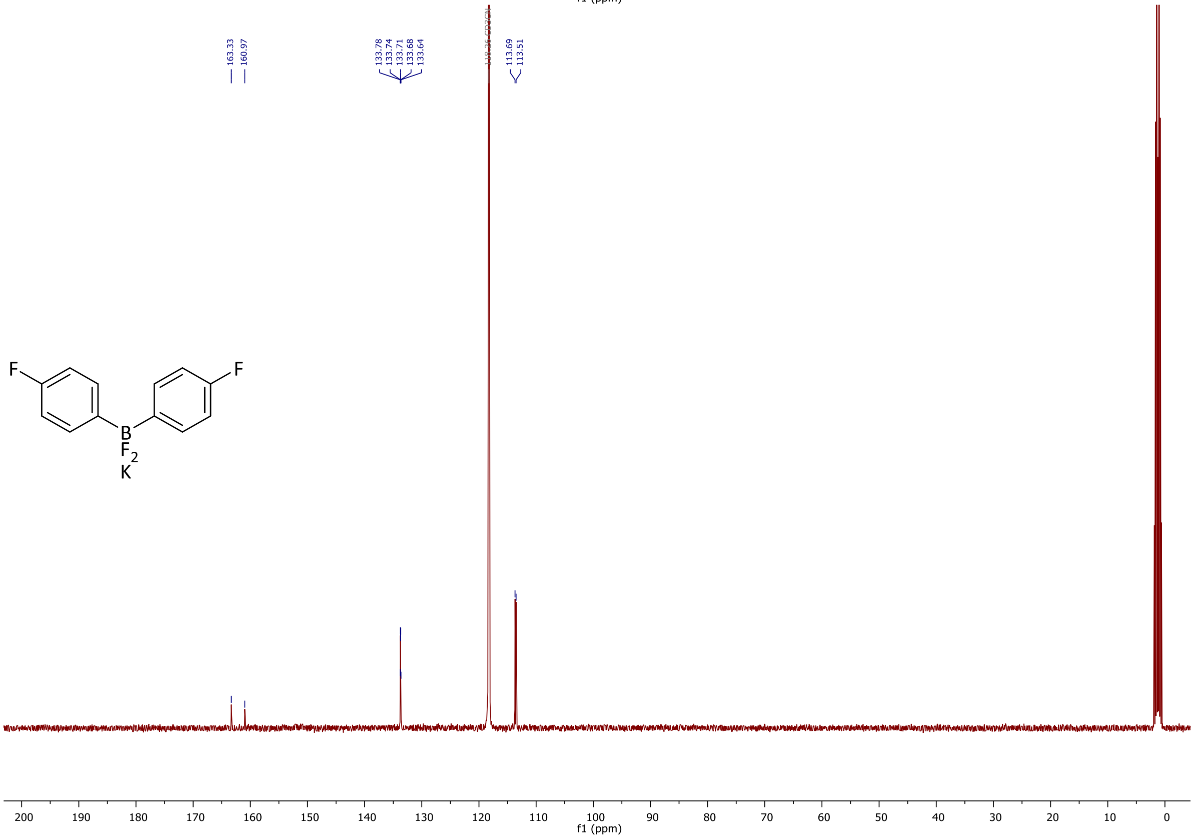

(*E*)-Tris(4-fluorophenyl)(styryl)borate (2a)

^{1}H NMR (400 MHz, Acetone-d_6) and **^{13}C NMR** (101 MHz, Acetone-d_6)

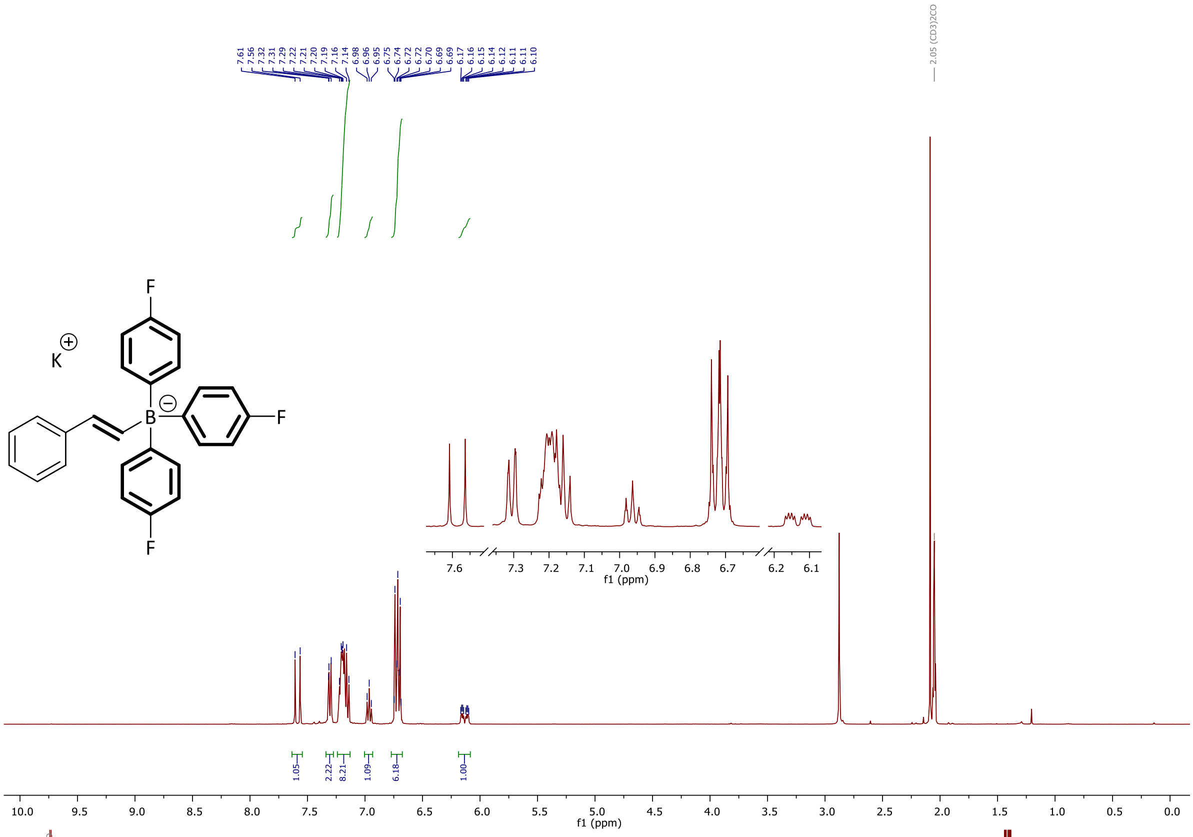

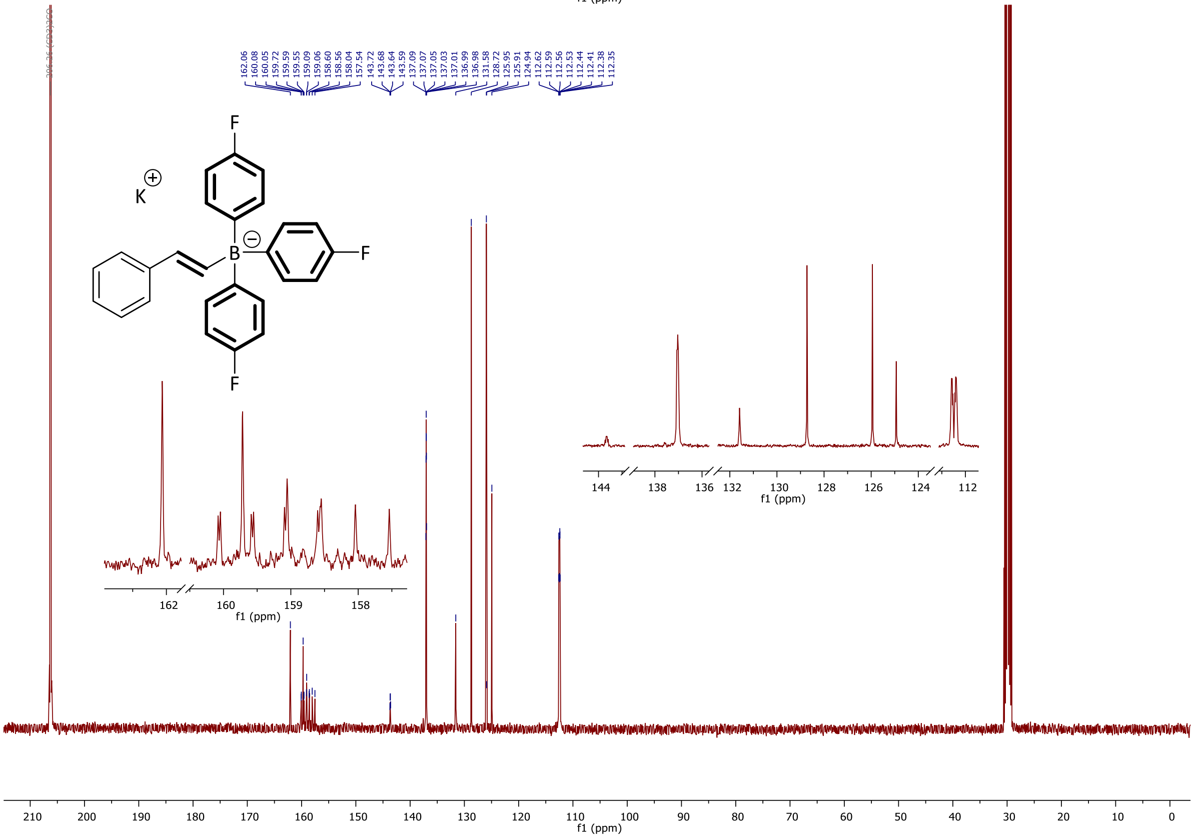

(*E*)-Tris(phenyl)(styryl)borate (2b)

^{1}H NMR (400 MHz, Acetone-d_6) and **^{13}C NMR** (101 MHz, Acetone-d_6)

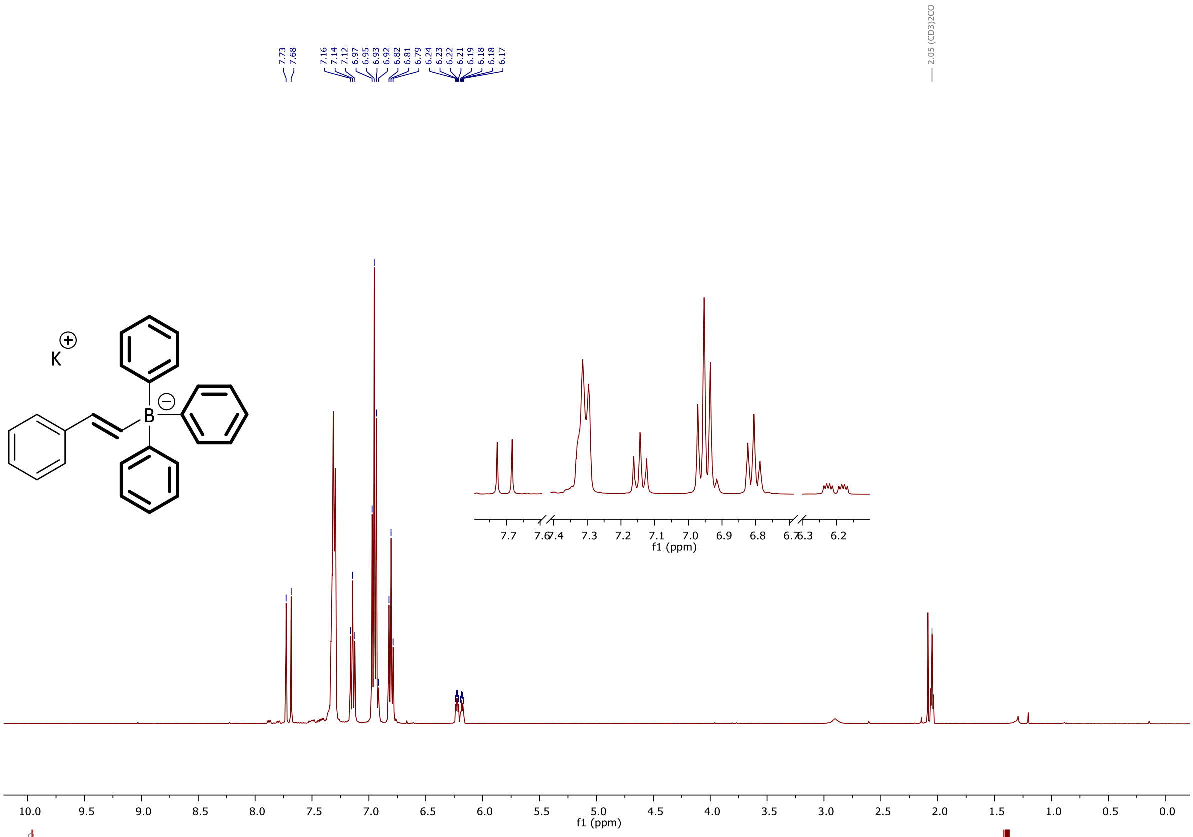

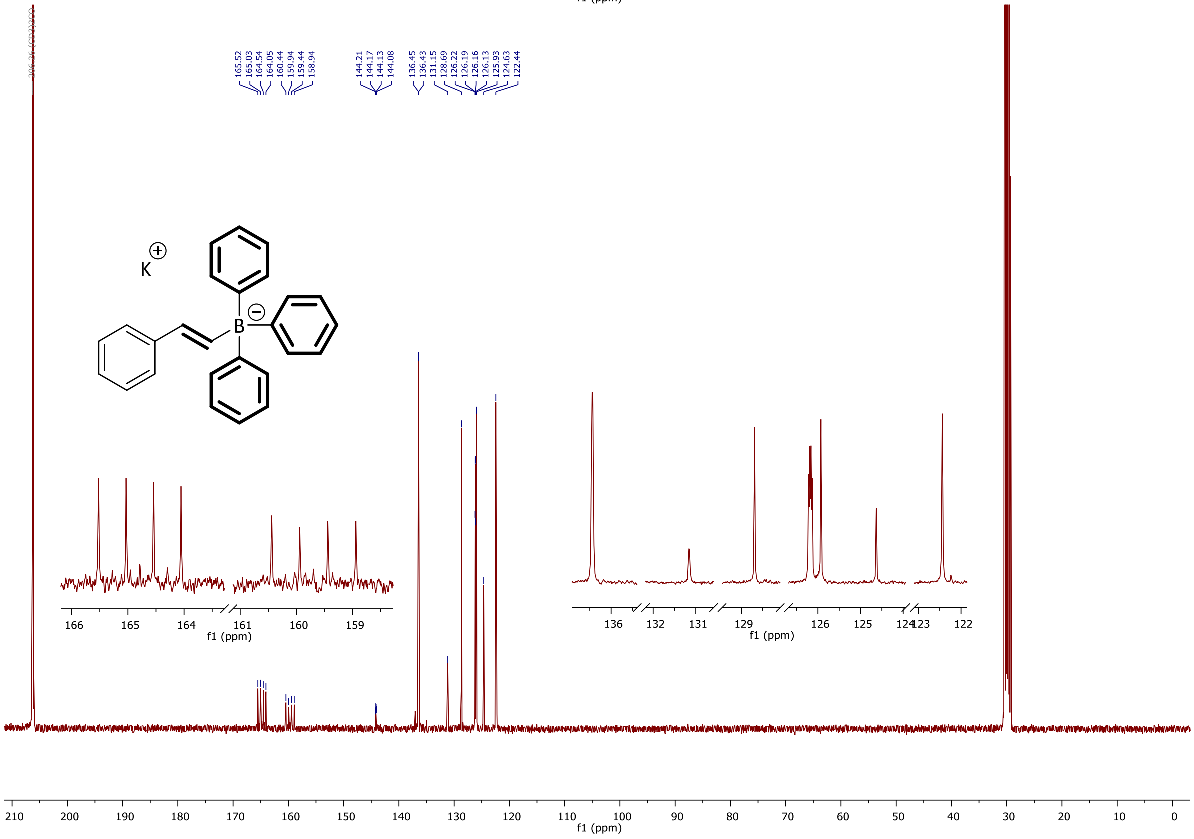

(*E*)-1-Fluoro-4-styrylbenzene (3a/3m) and (*Z*)-1-Fluoro-4-styrylbenzene (3a-*Z*)

^{1}H NMR (400 MHz, $CDCl_3$)

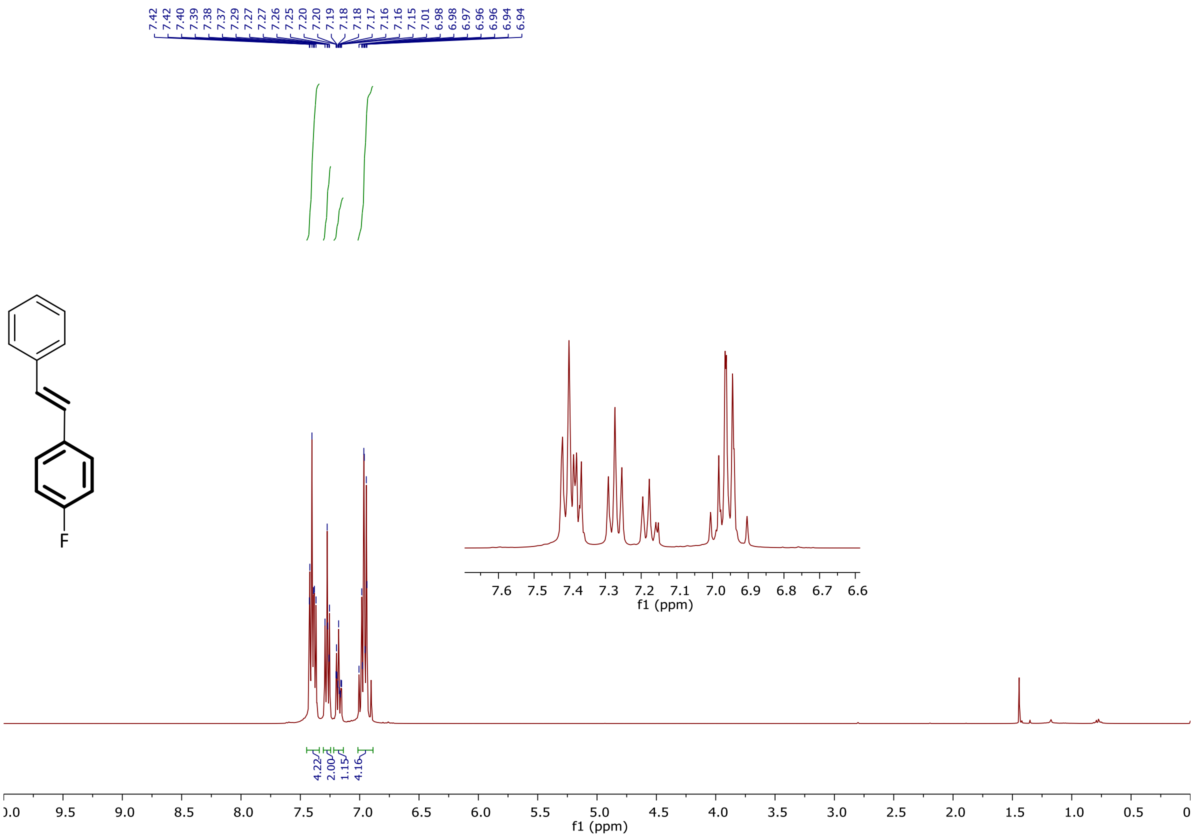

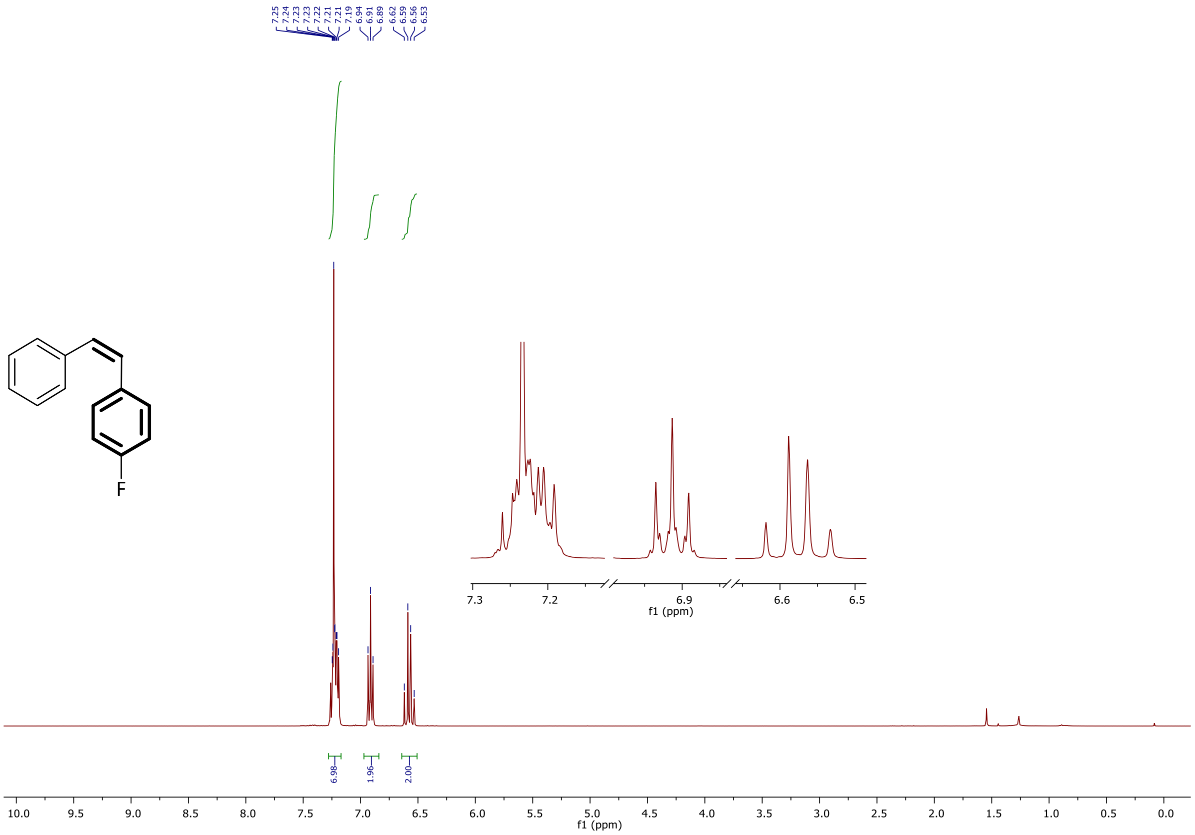

***trans*-2-(4-fluorophenyl)-3-phenyloxirane (3ab)**

^{1}H NMR (400 MHz, $CDCl_3$)

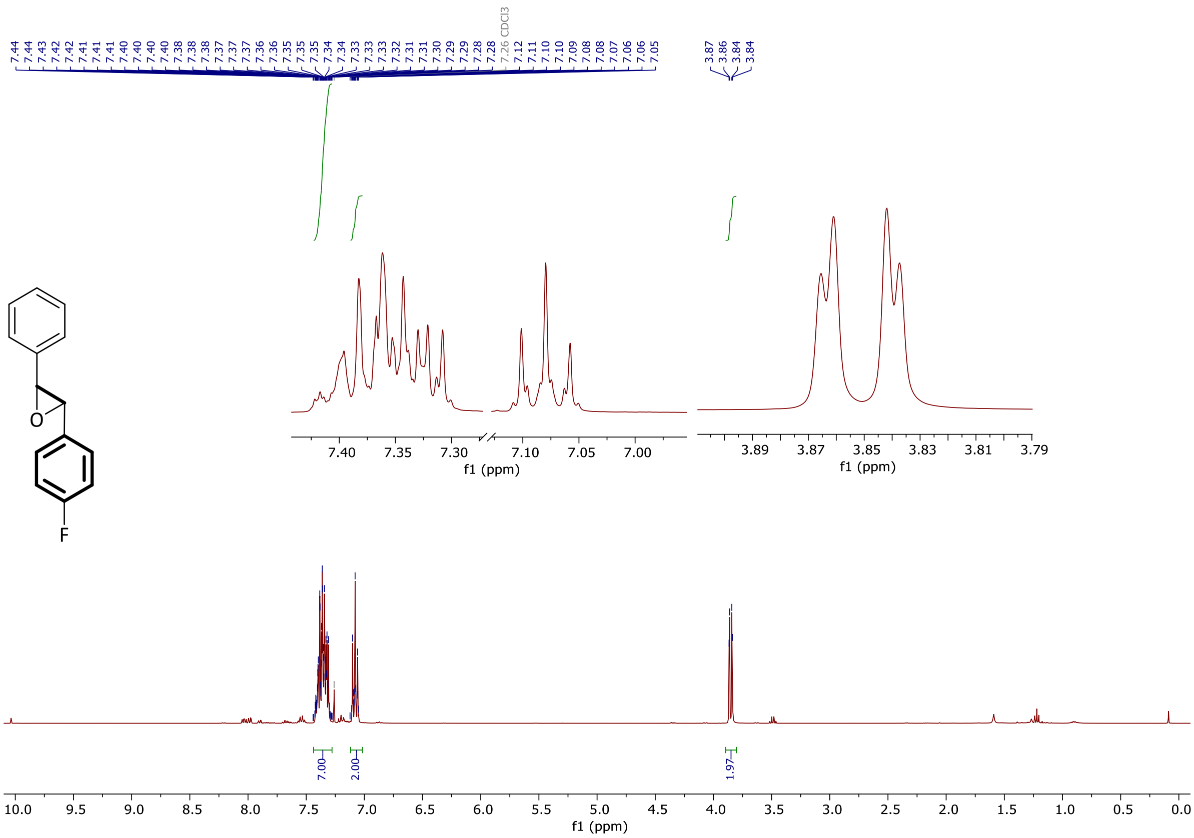

(*E*)-1-Styryl-4-(trifluoromethyl)benzene (3b)

^{1}H NMR (400 MHz, $CDCl_3$)

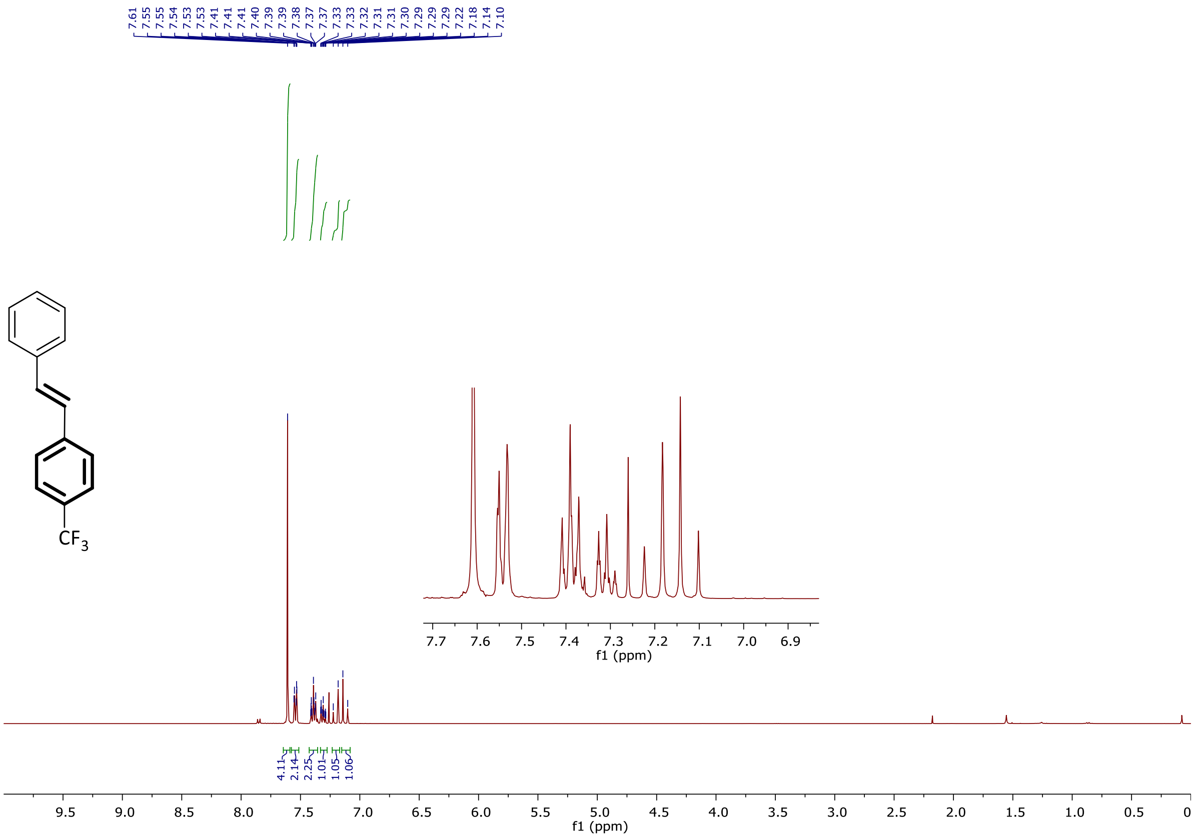

(*E*)-4-Styrylbenzonitrile (3c)

^{1}H NMR (400 MHz, $CDCl_3$)

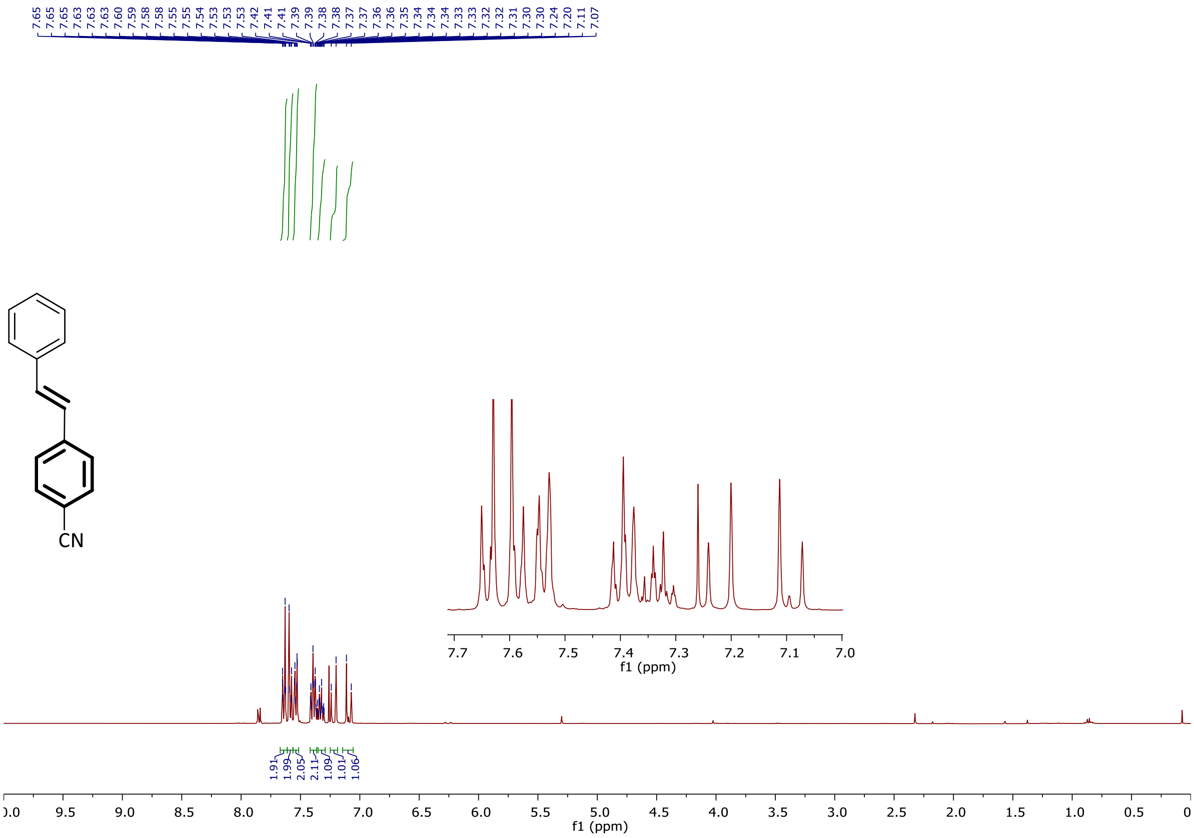

(*E*)-1,3-Dimethoxy-5-styrylbenzene (3d)

^{1}H NMR (400 MHz, $CDCl_3$)

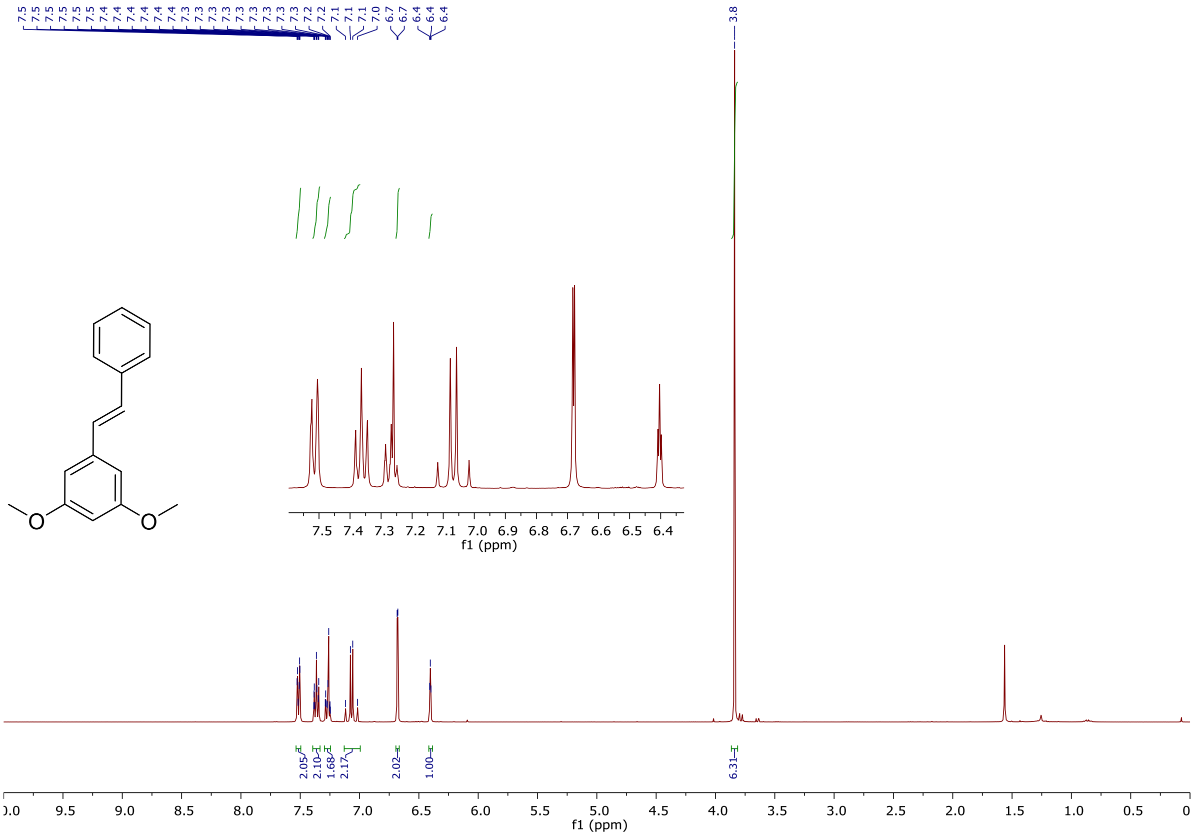

(*E*)-1-Styrylnaphthalene (3e/3q)

^{1}H NMR (400 MHz, $CDCl_3$)

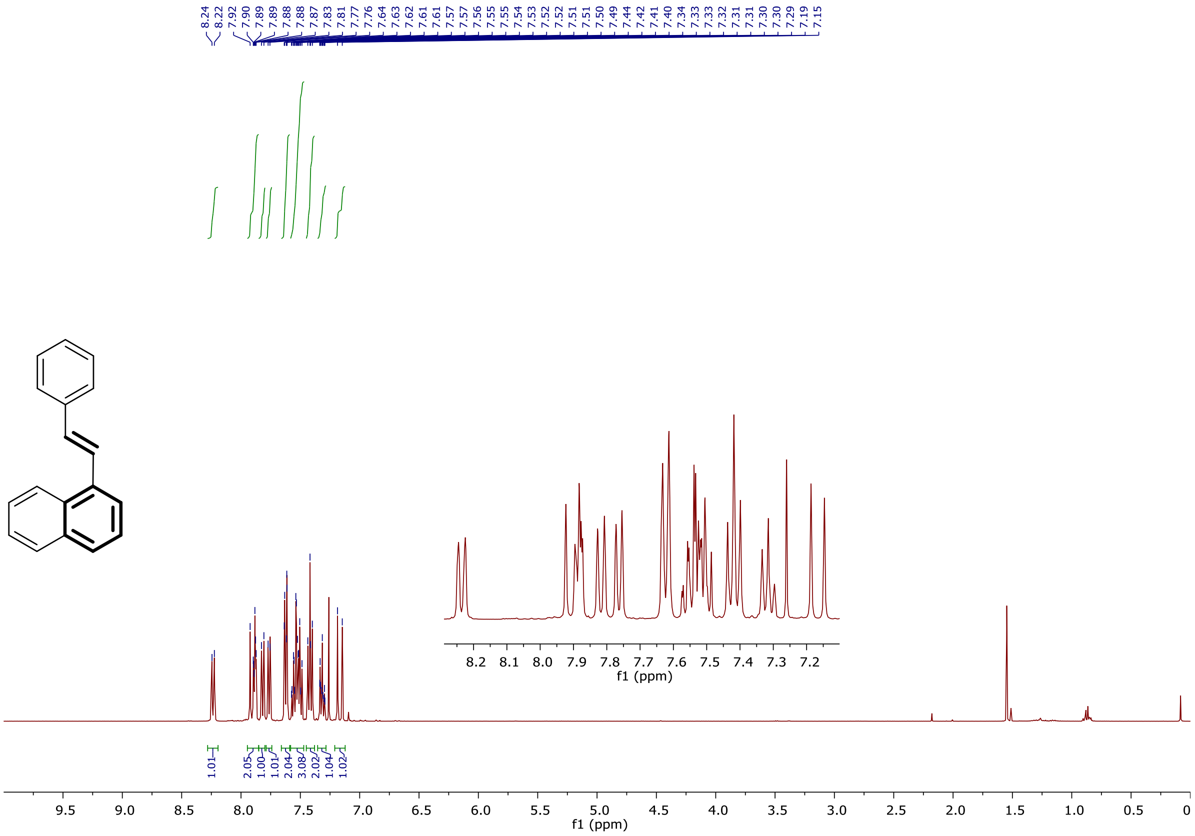

(*E*)-4-(4-Fluorostyryl)-1,1'-biphenyl (3f)

^{1}H NMR (400 MHz, $CDCl_3$)

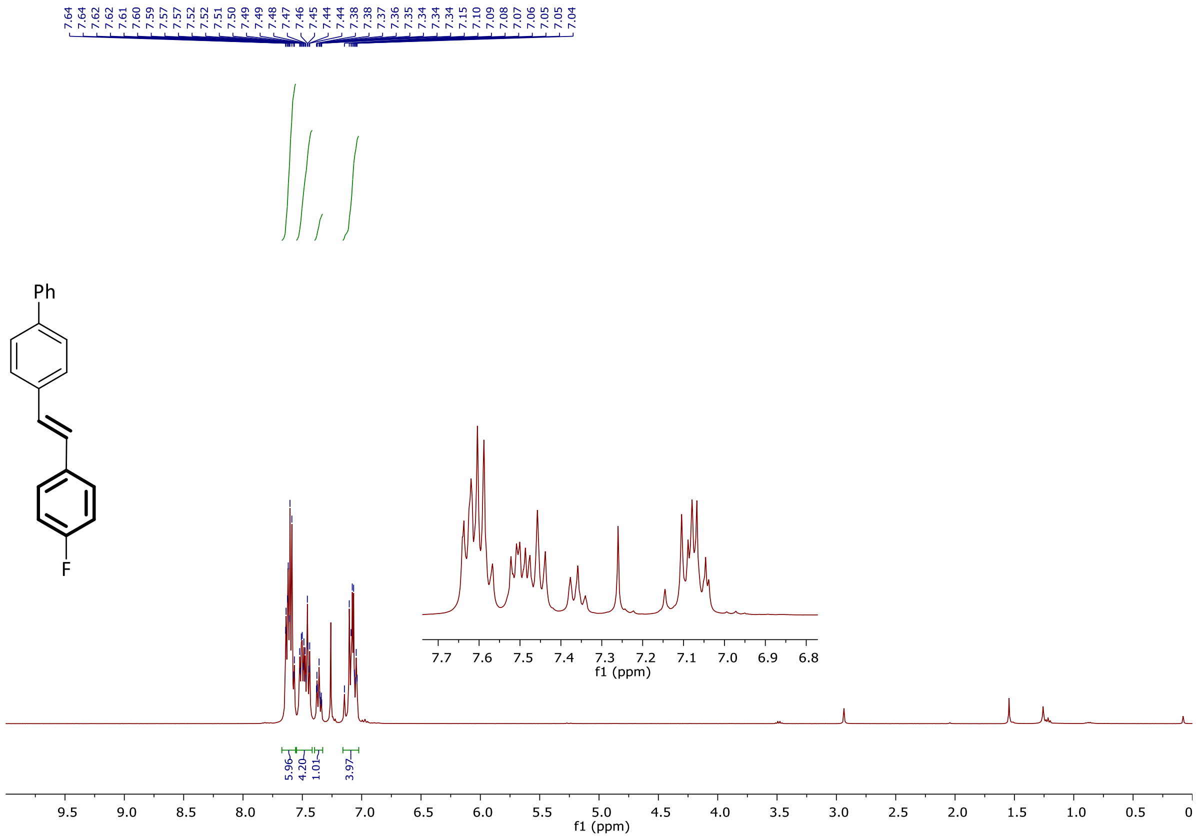

(*E*)-2-Chloro-1-fluoro-4-(4-fluorostyryl)benzene (3g)

^{1}H NMR (400 MHz, $CDCl_3$) and **^{13}C NMR** (101 MHz, $CDCl_3$)

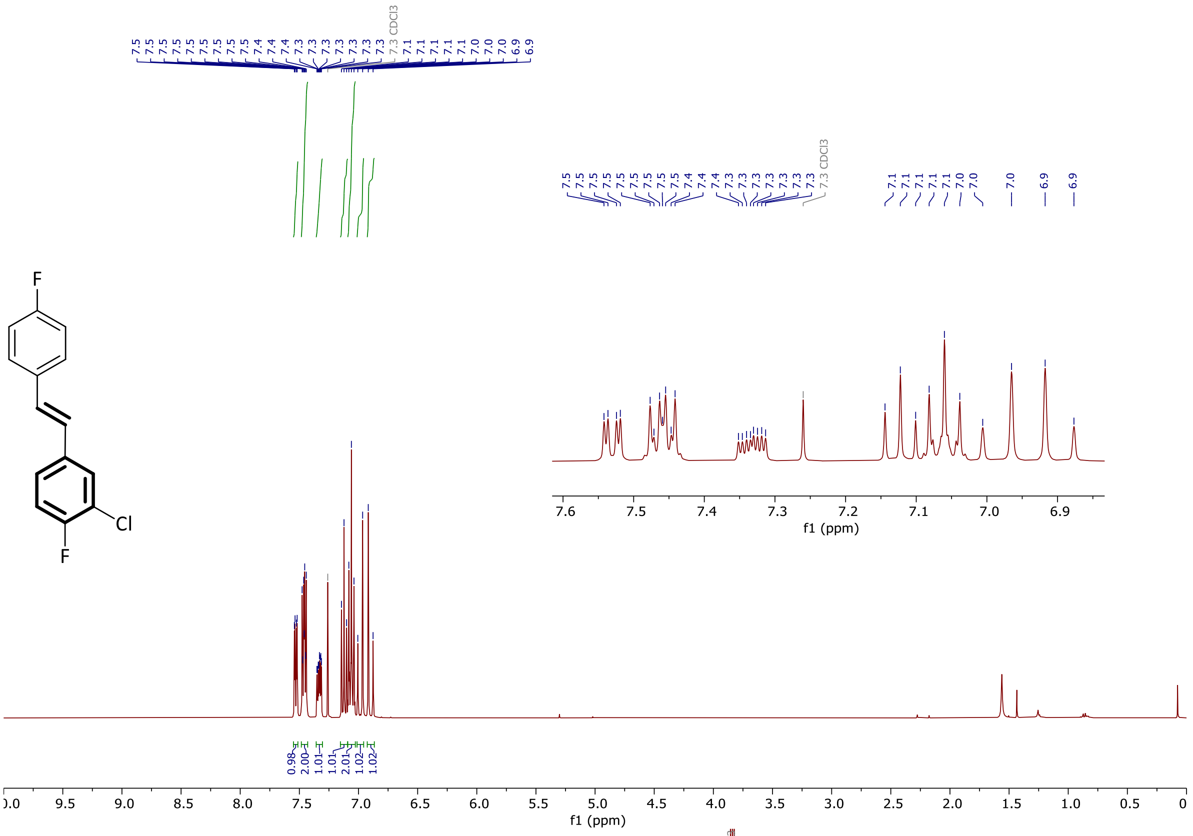

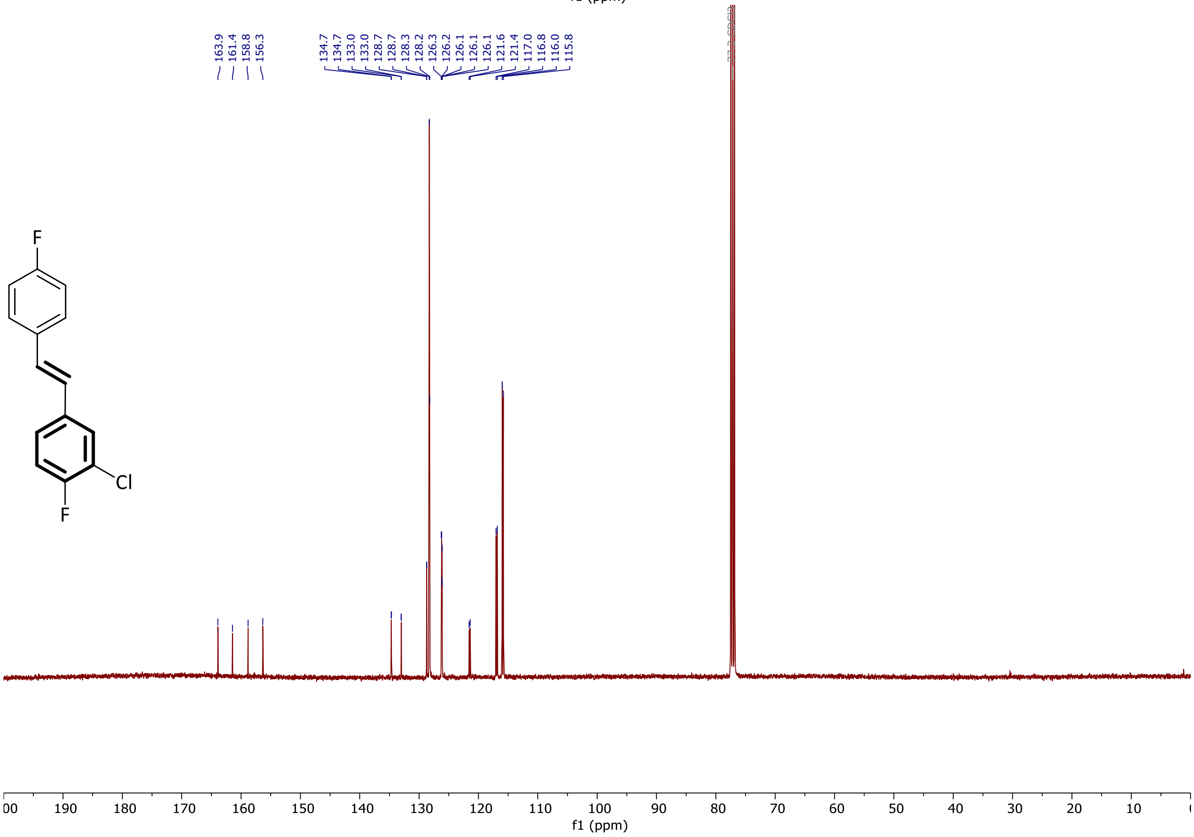

(*E*)-5-Styrylbenzofuran (3h)

^{1}H NMR (400 MHz, $CDCl_3$) and **^{13}C NMR** (101 MHz, $CDCl_3$)

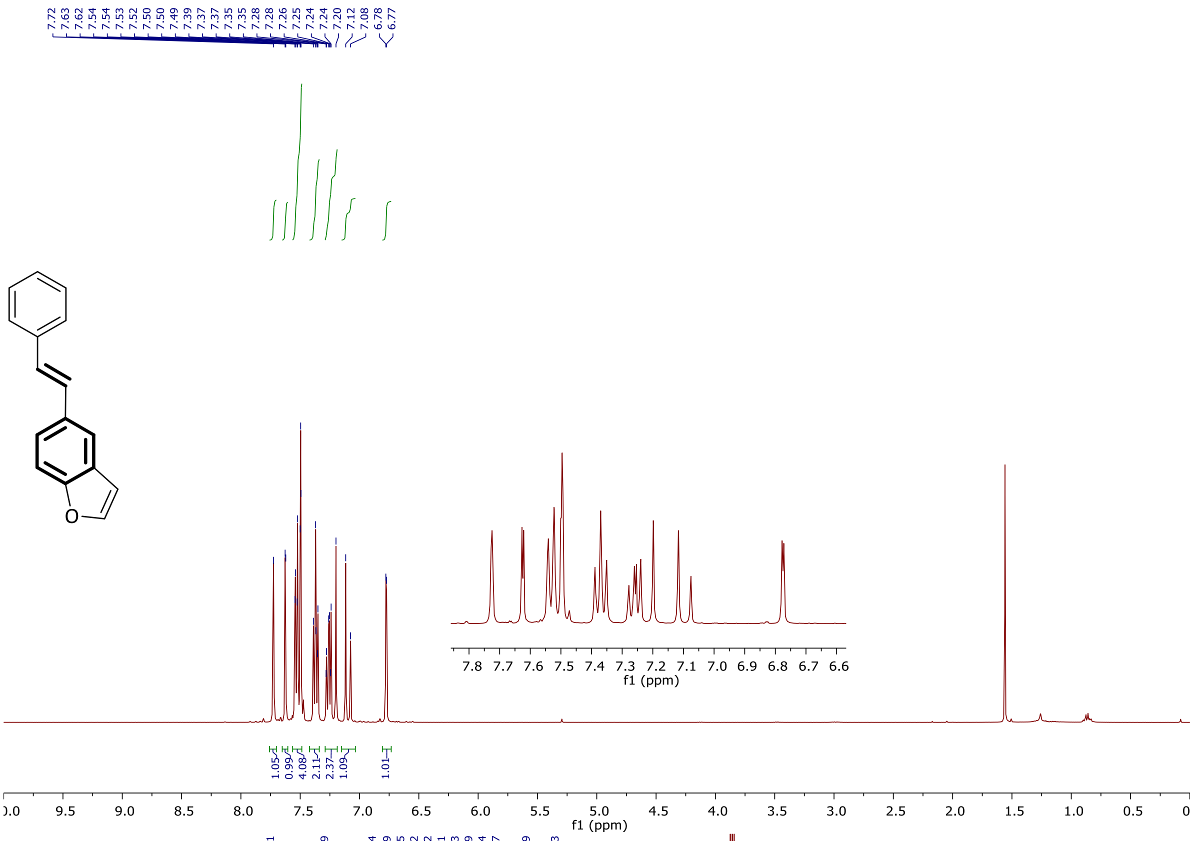

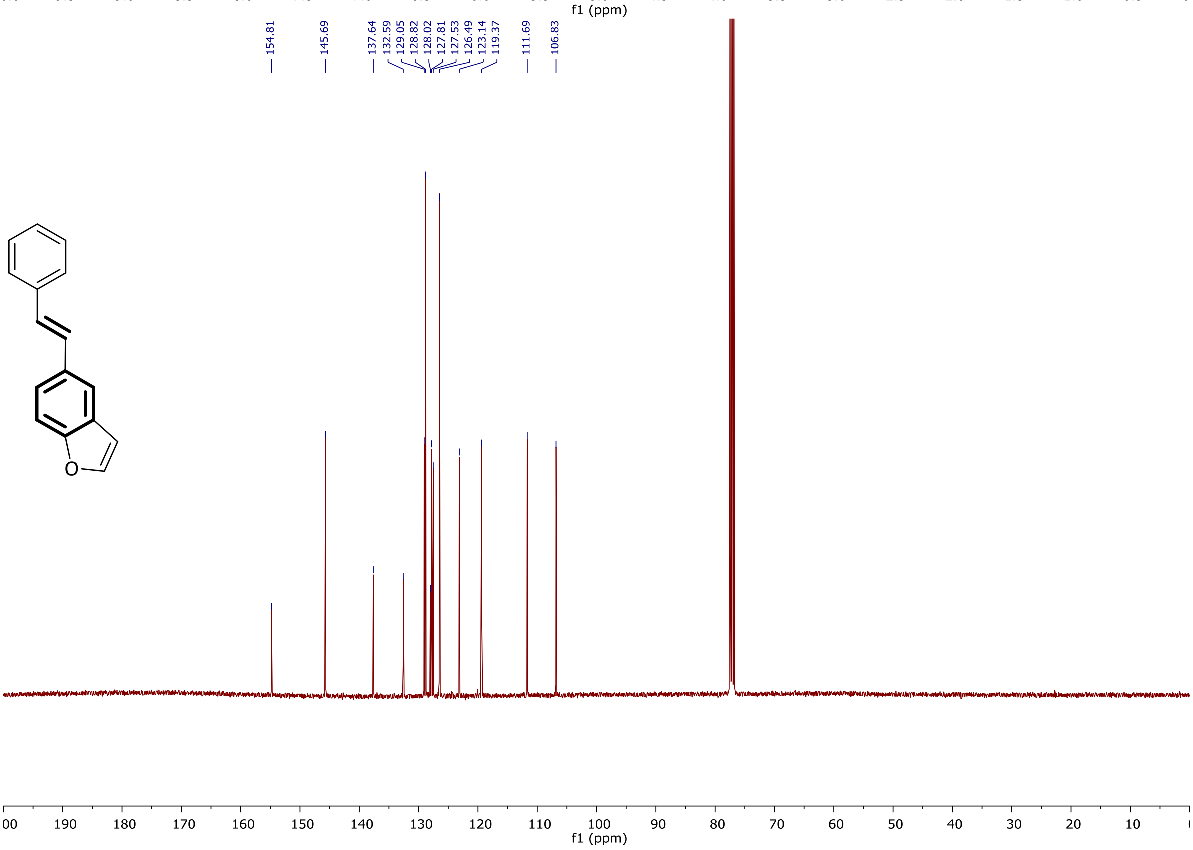

(*E*)-2-Styryldibenzo[*b*,*d*]furan (3i/3s)

^{1}H NMR (400 MHz, $CDCl_3$)

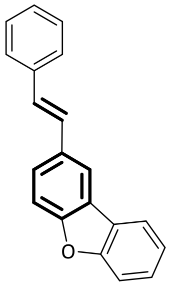

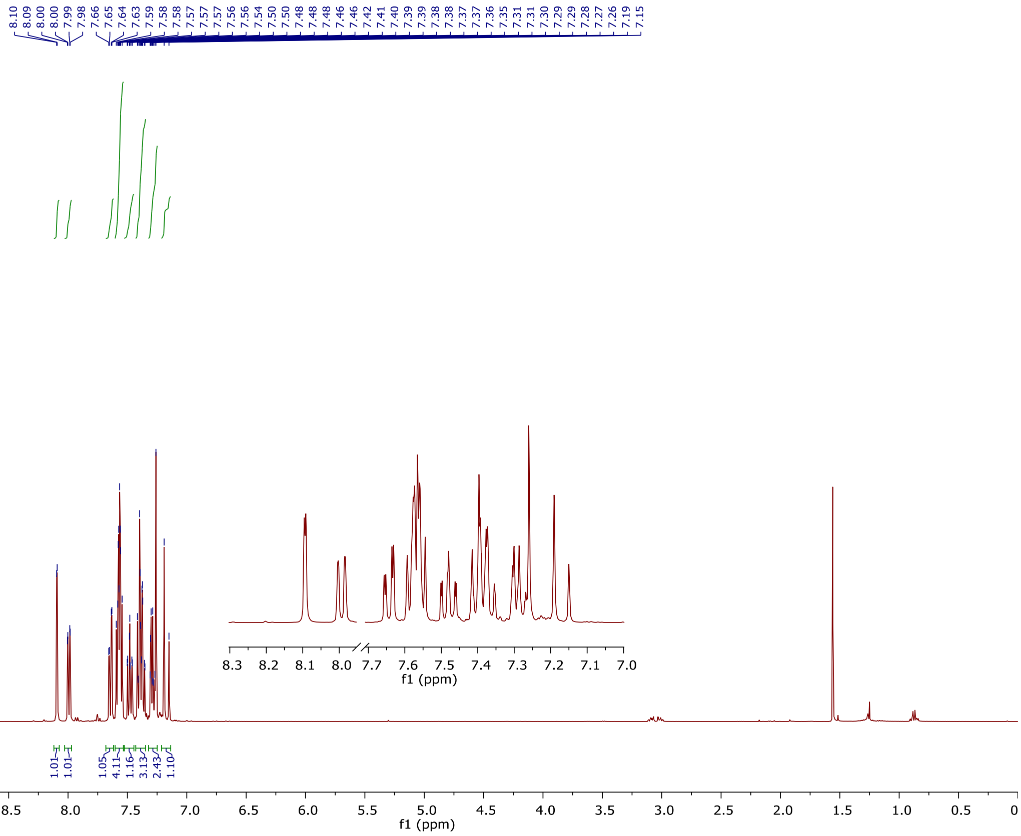

(*E*)-2-(4-Fluorostyryl)dibenzo[*b*,*d*]furan (3j)

^{1}H NMR (400 MHz, $CDCl_3$) and **^{13}C NMR** (101 MHz, $CDCl_3$)

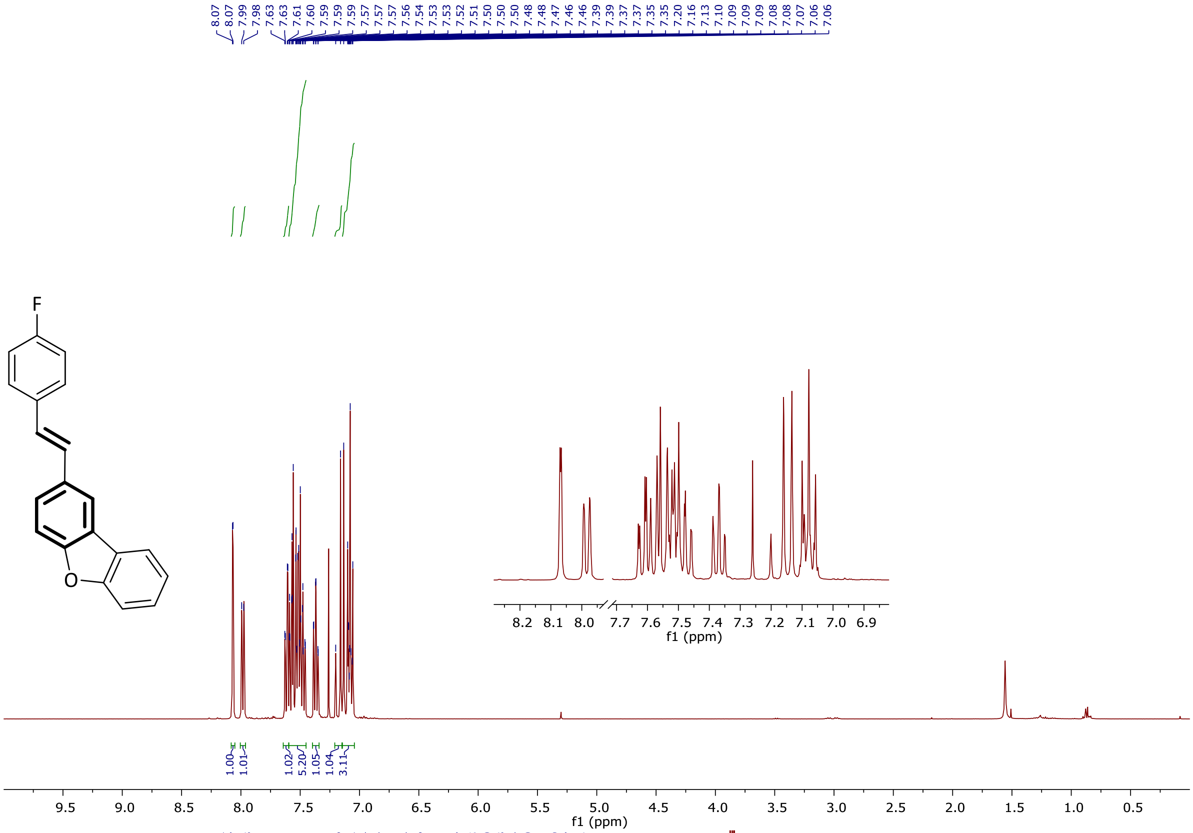

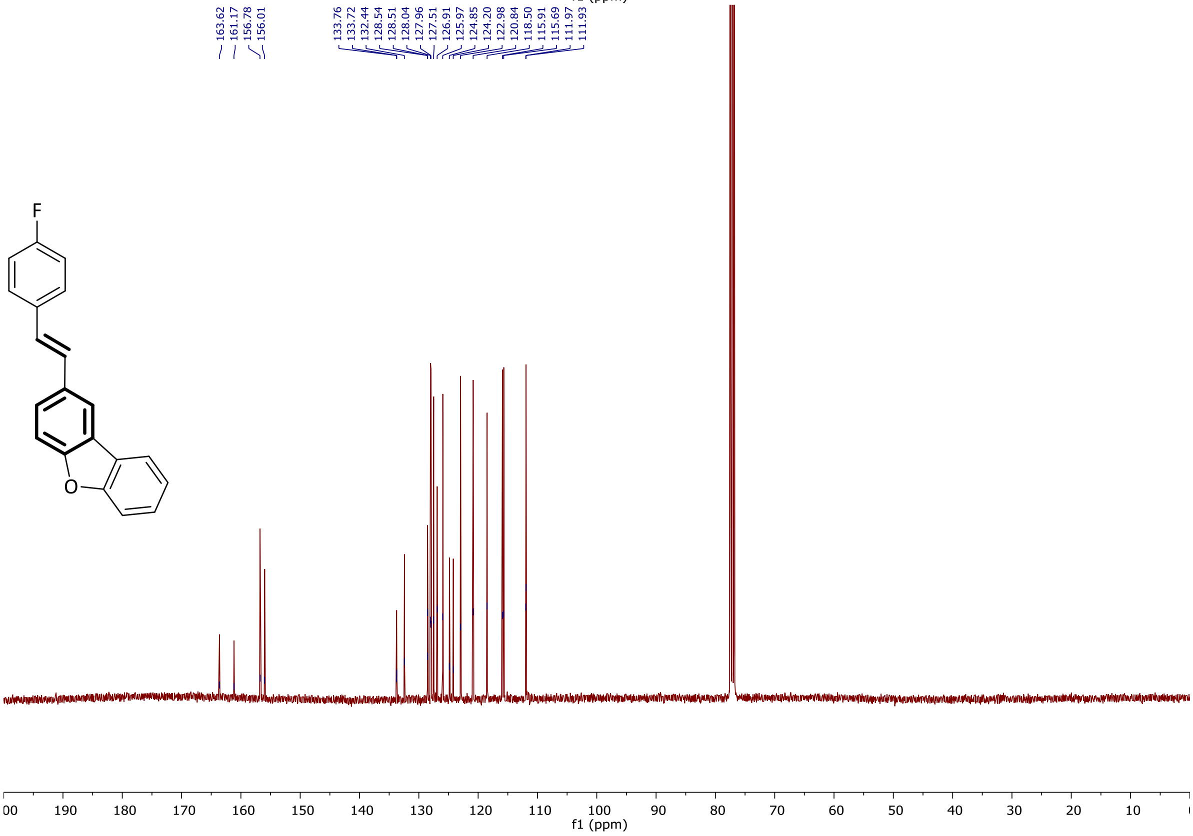

(*E*)-1,3-Difluoro-5-(2-phenylprop-1-en-1-yl)benzene (3k)

^{1}H NMR (400 MHz, $CDCl_3$) and **^{13}C NMR** (101 MHz, $CDCl_3$)

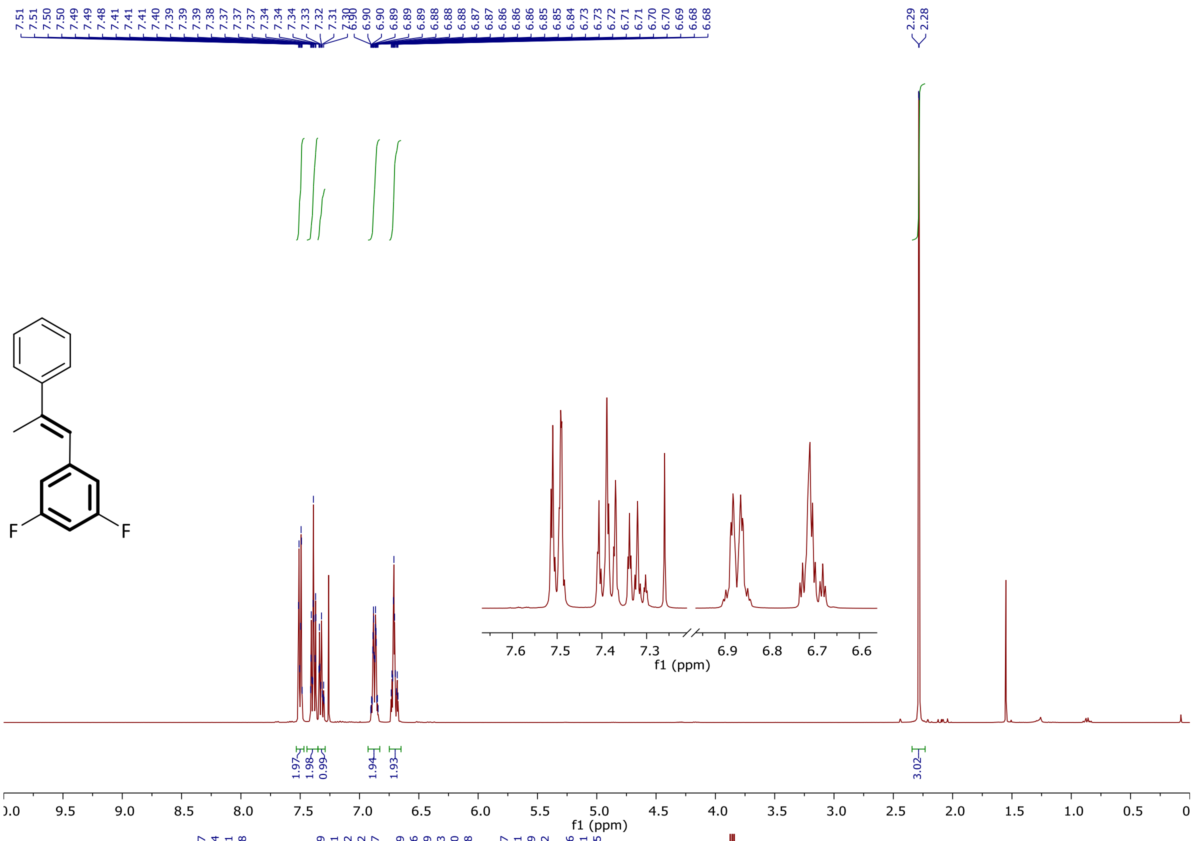

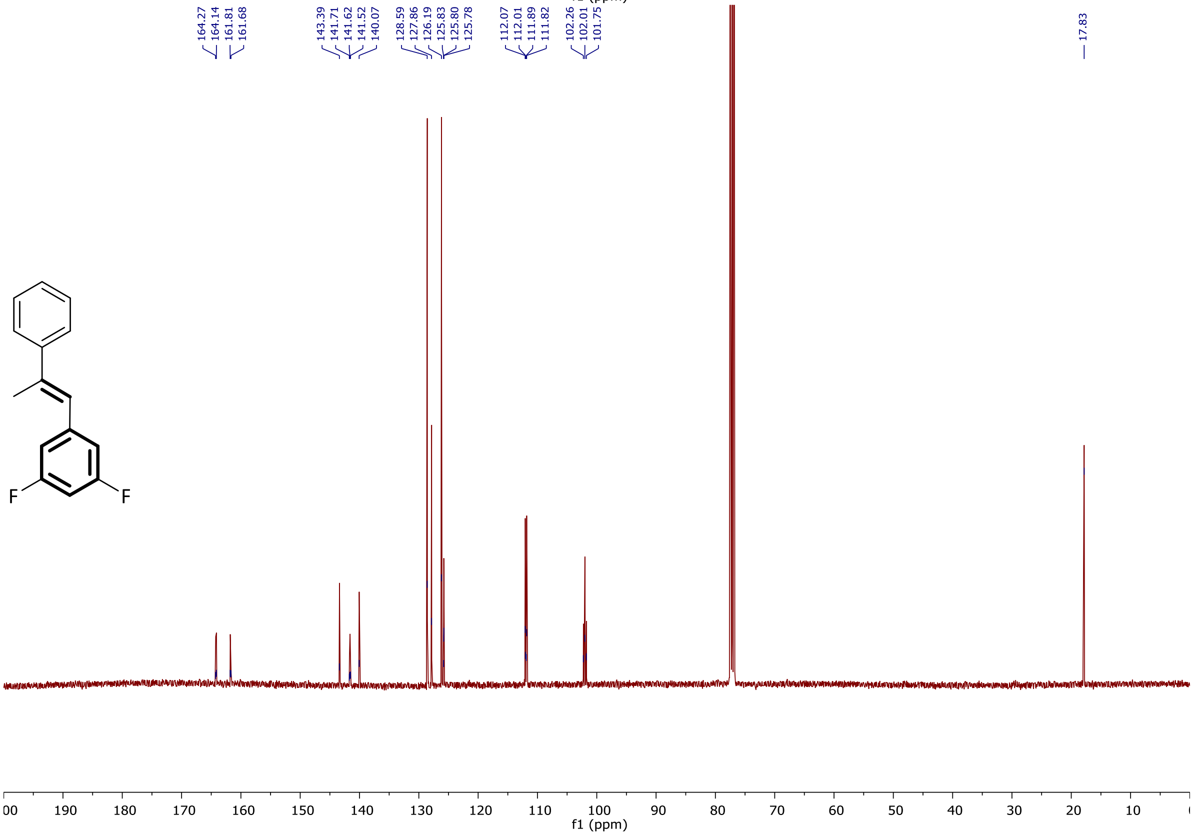

Ethyl (*E*)-3-(pyridin-3-yl)acrylate (3l)

^{1}H NMR (400 MHz, $CDCl_3$) and **^{13}C NMR** (101 MHz, $CDCl_3$)

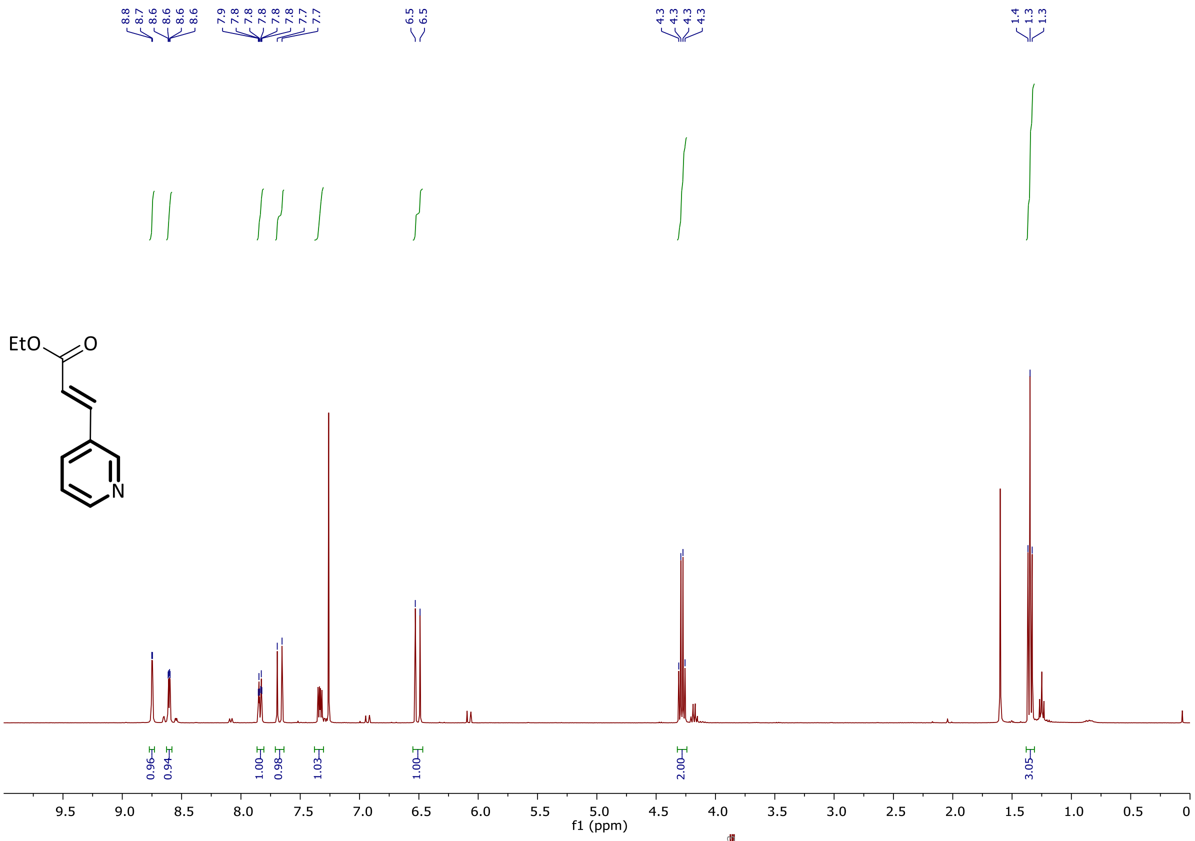

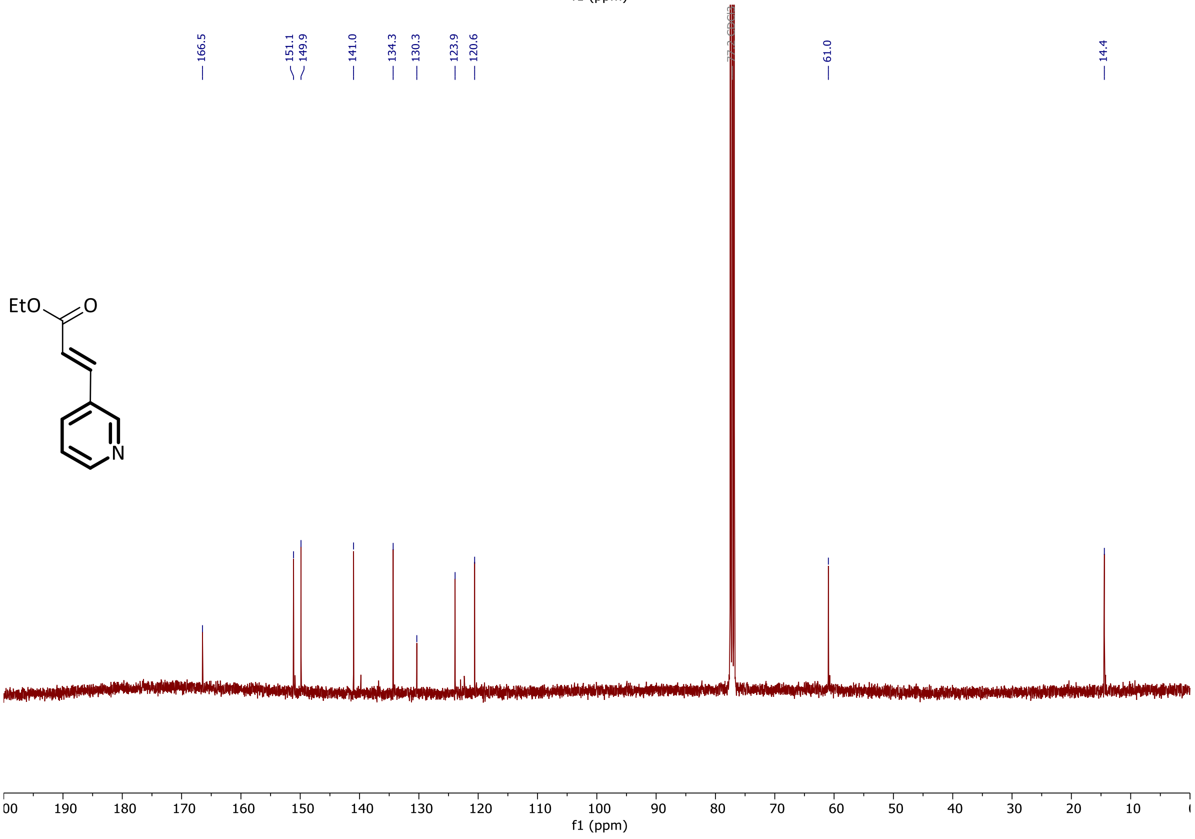

(*E*)-1,3-difluoro-5-styrylbenzene (3n)

^{1}H NMR (400 MHz, $CDCl_3$)

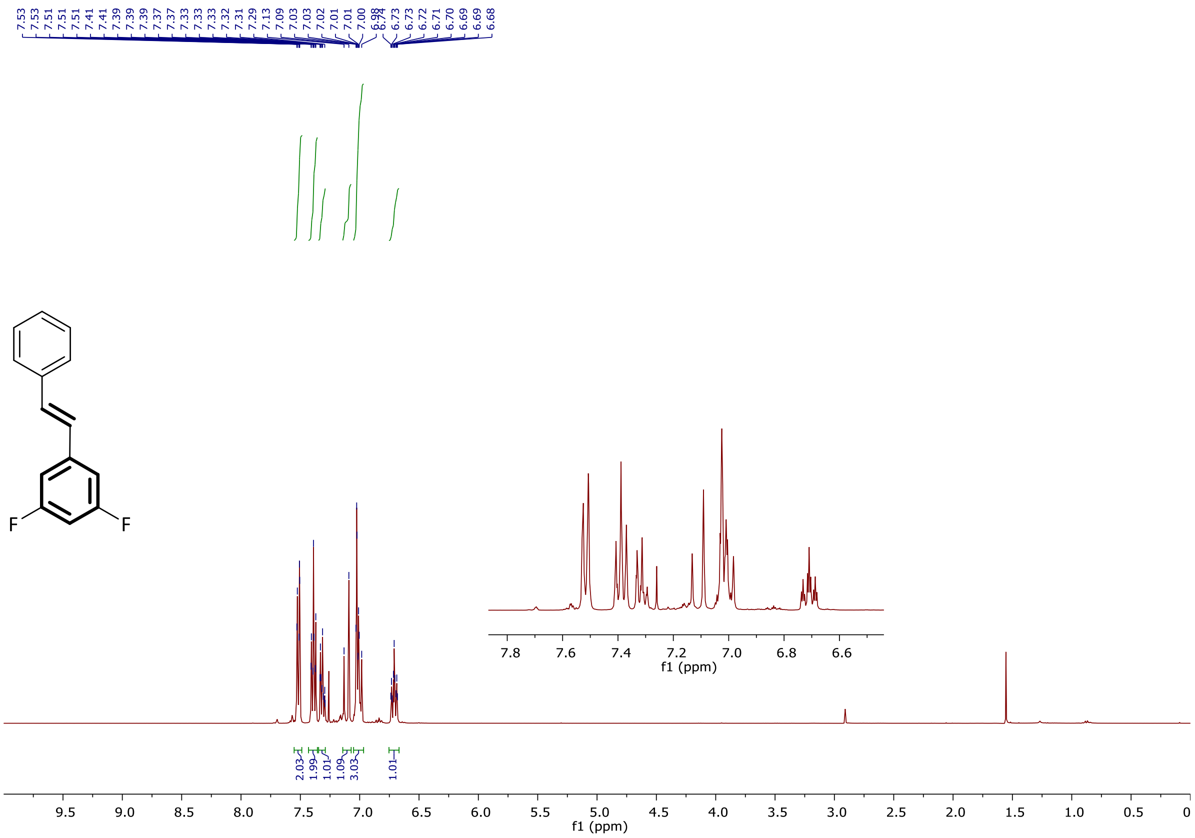

(*E*)-1-Styryl-4-(trifluoromethoxy)benzene (3o)

^{1}H NMR (400 MHz, $CDCl_3$)

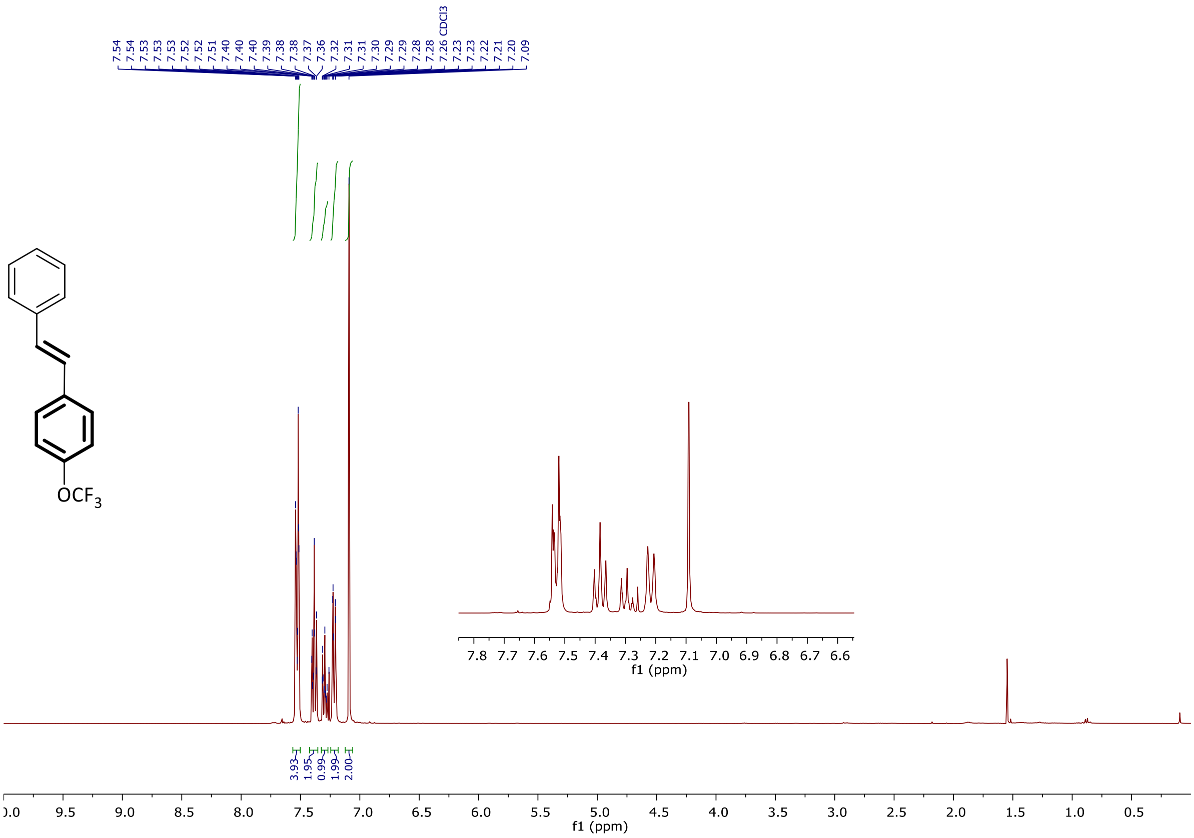

(*E*)-1-Styryl-3,5-bis(trifluoromethyl)benzene (3p)

^{1}H NMR (400 MHz, $CDCl_3$)

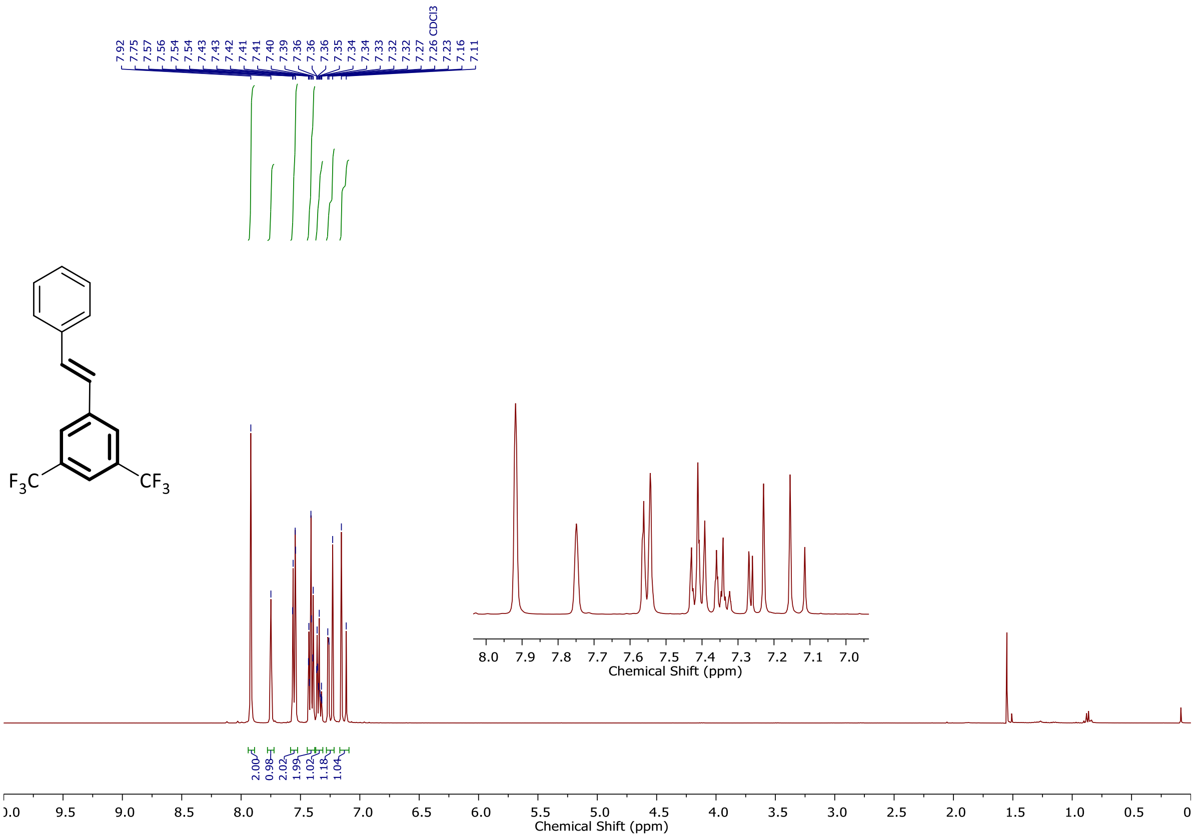

(*E*)-2-(3,5-Bis(trifluoromethyl)styryl)-6-methoxynaphthalene (3r)

^{1}H NMR (400 MHz, $CDCl_3$) and **^{13}C NMR** (101 MHz, $CDCl_3$)

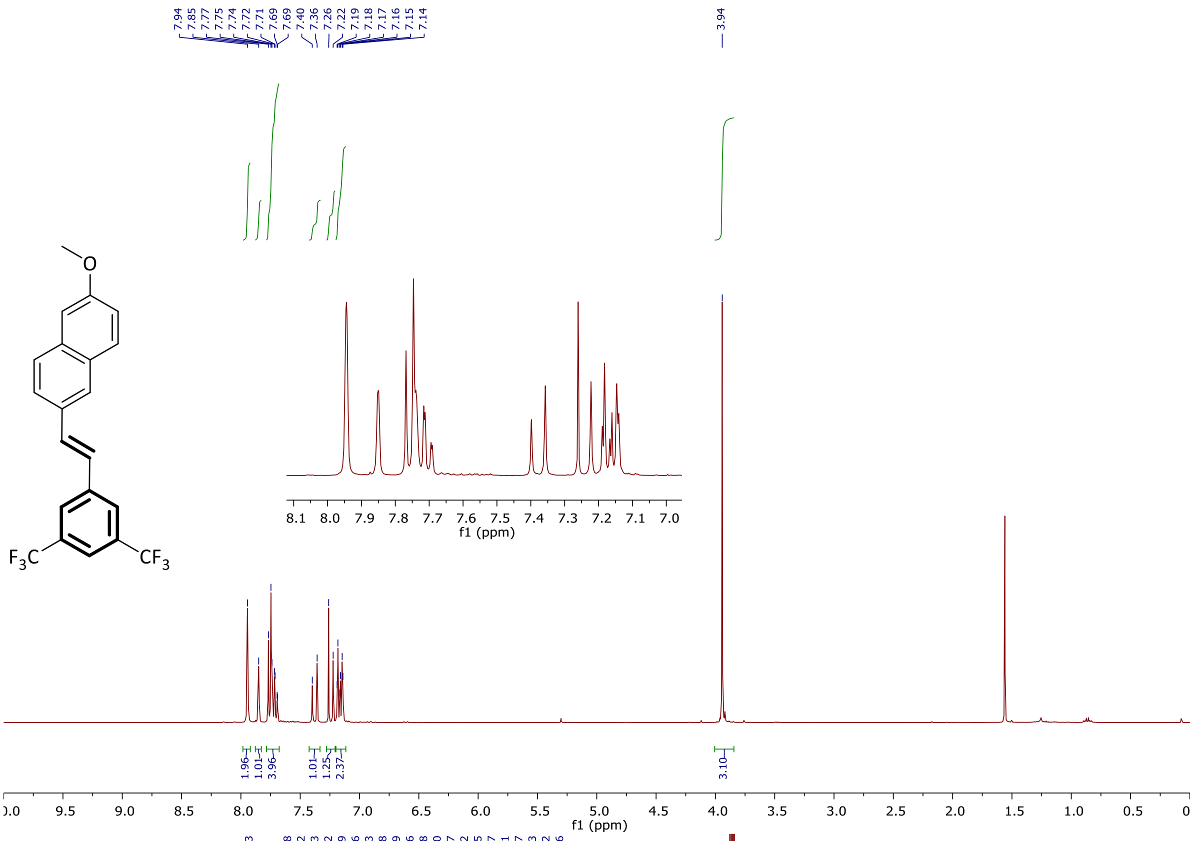

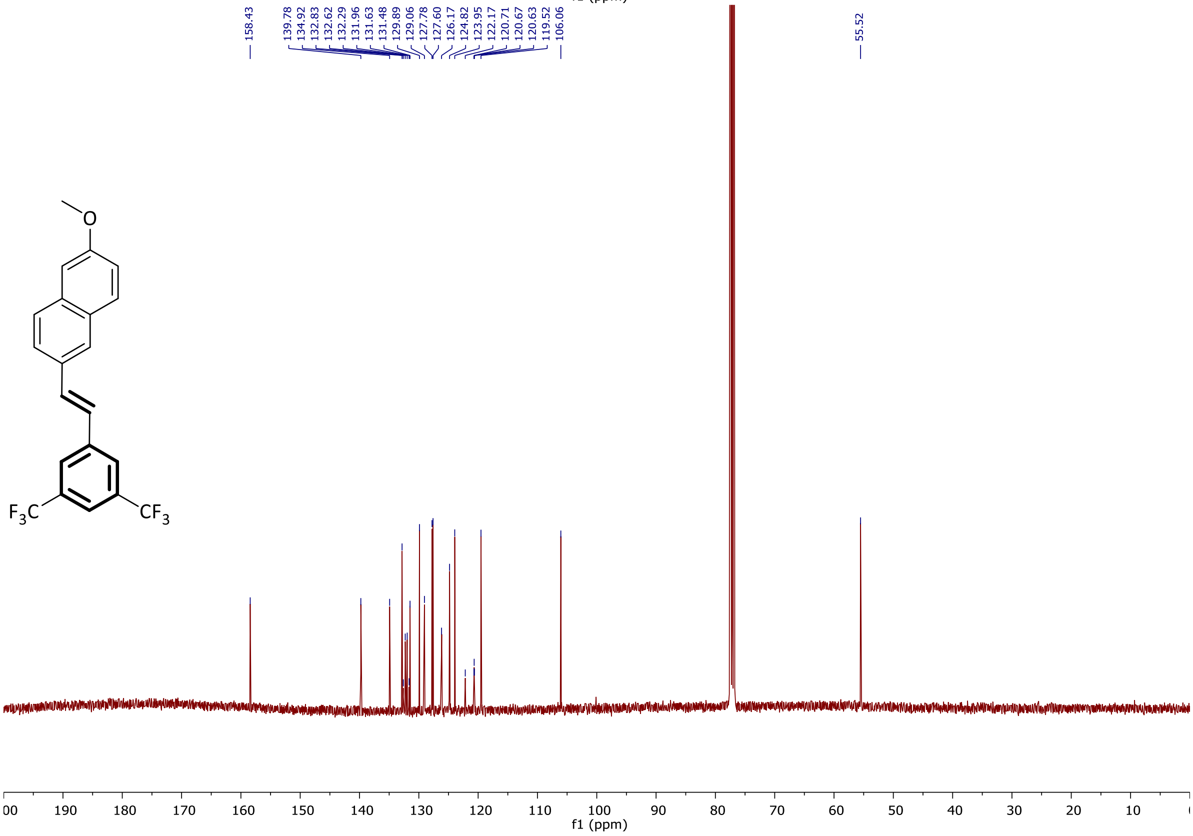

(1-Methoxy-4-(prop-1-en-2-yl)benzene (4a)

¹H NMR (400 MHz, $CDCl_3$)

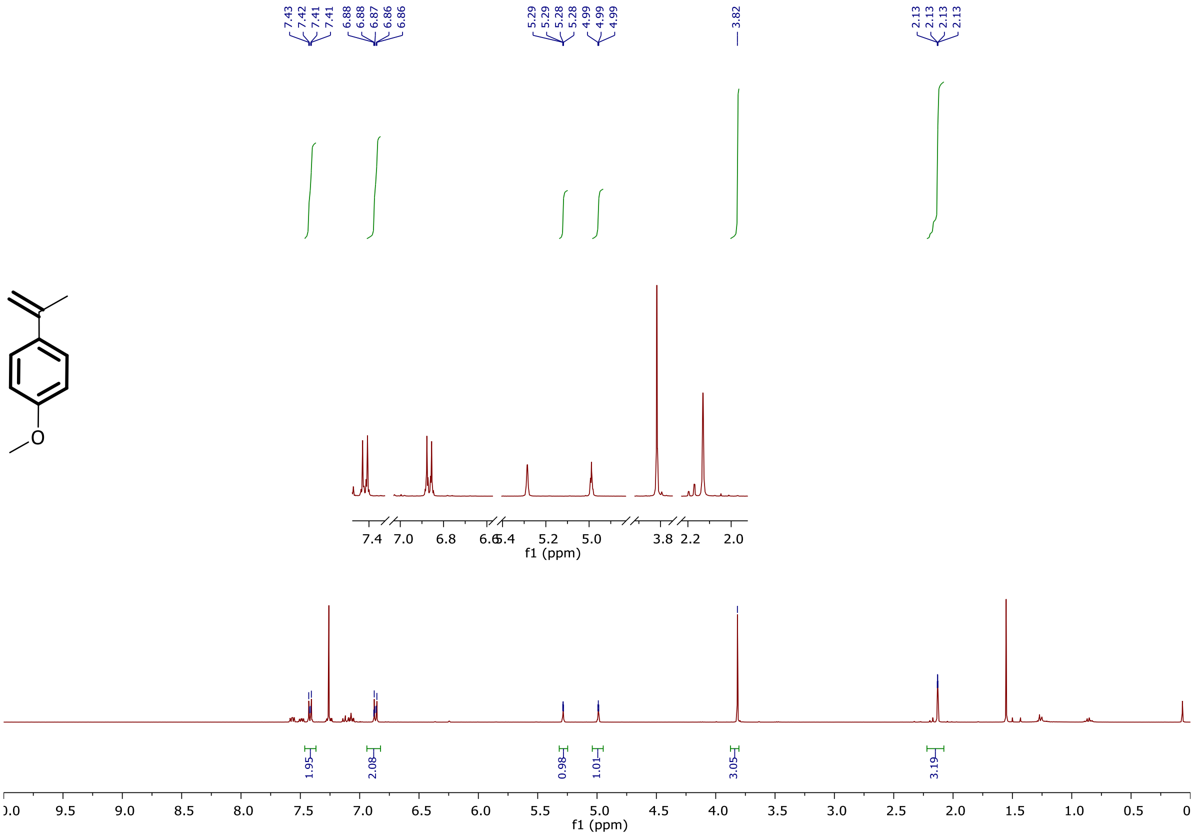

1-(Cyclopent-1-en-1-yl)-3,5-bis(trifluoromethyl)benzene (4b)

^{1}H NMR (400 MHz, $CDCl_3$) and **^{13}C NMR** (101 MHz, $CDCl_3$)

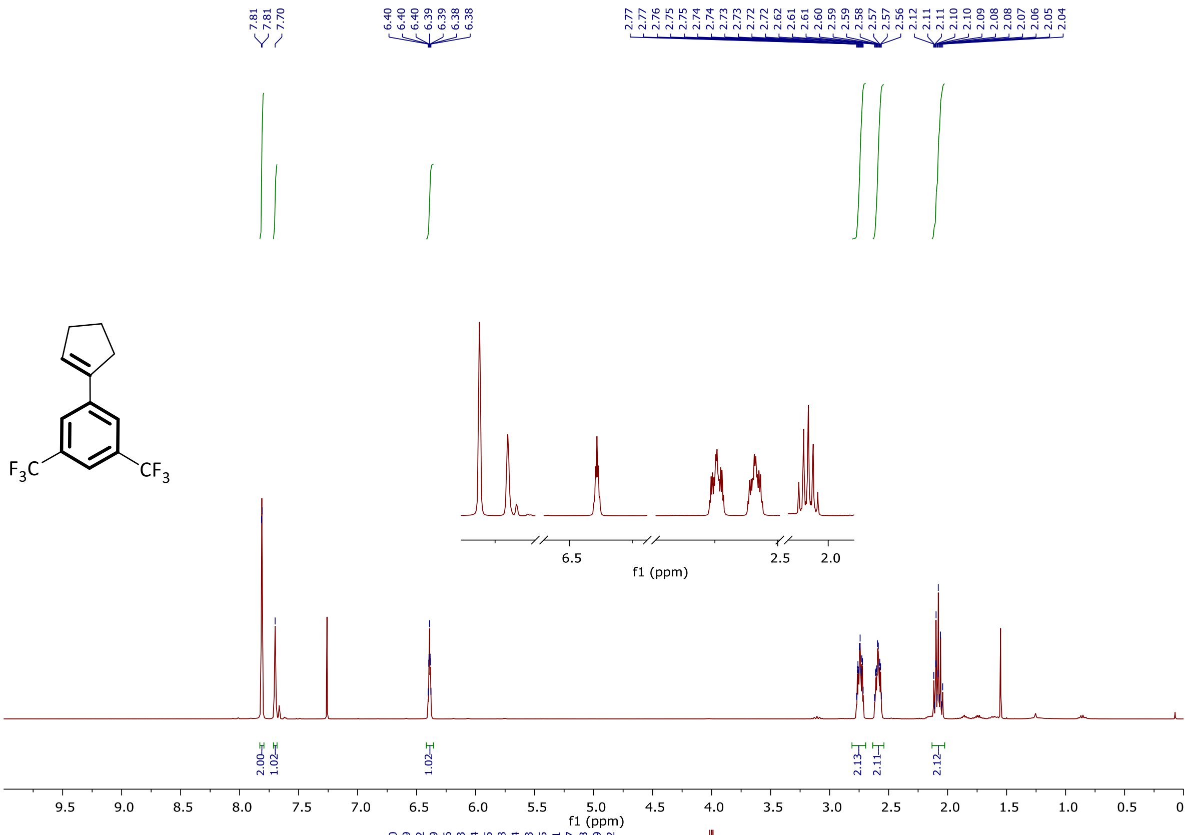

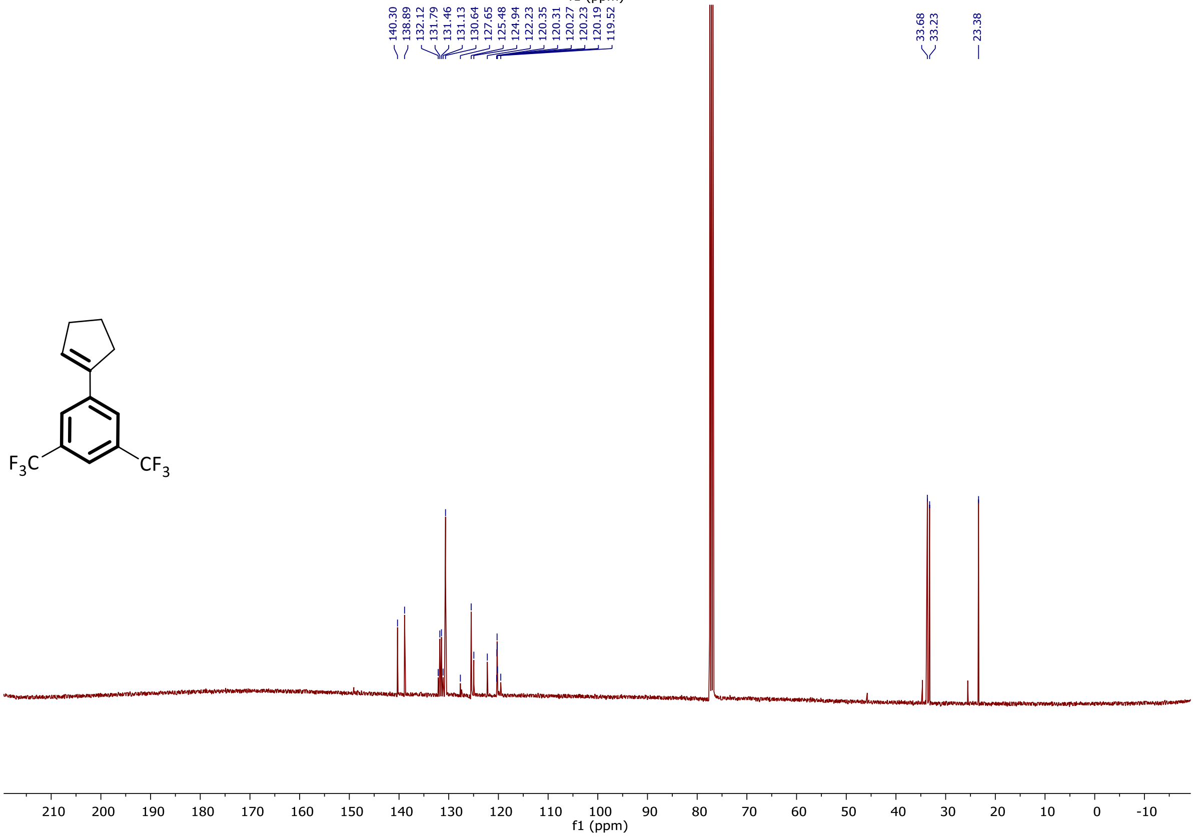

4'-Methoxy-2,3,4,5-tetrahydro-1,1'-biphenyl (4c)

^{1}H NMR (400 MHz, $CDCl_3$)

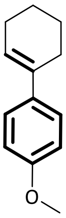

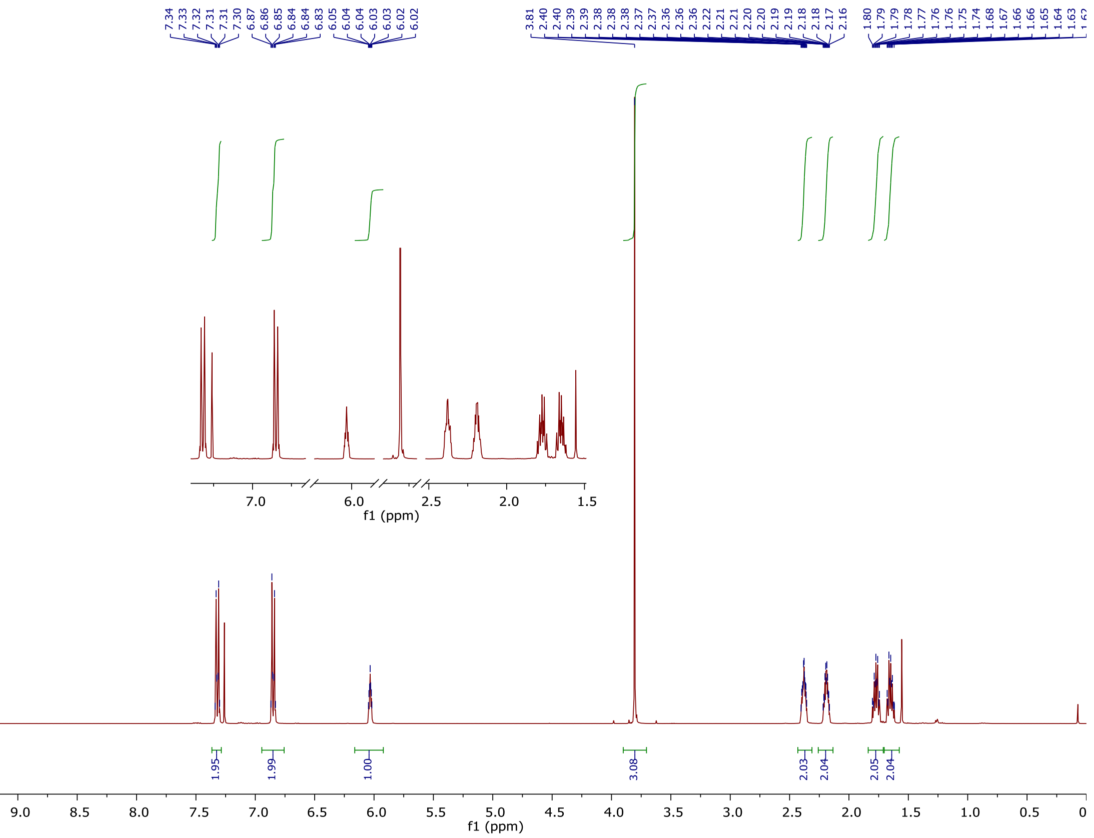

3',4'-Dichloro-2,3,4,5-tetrahydro-1,1'-biphenyl (4d)

^{1}H NMR (400 MHz, $CDCl_3$) and **^{13}C NMR** (101 MHz, $CDCl_3$)

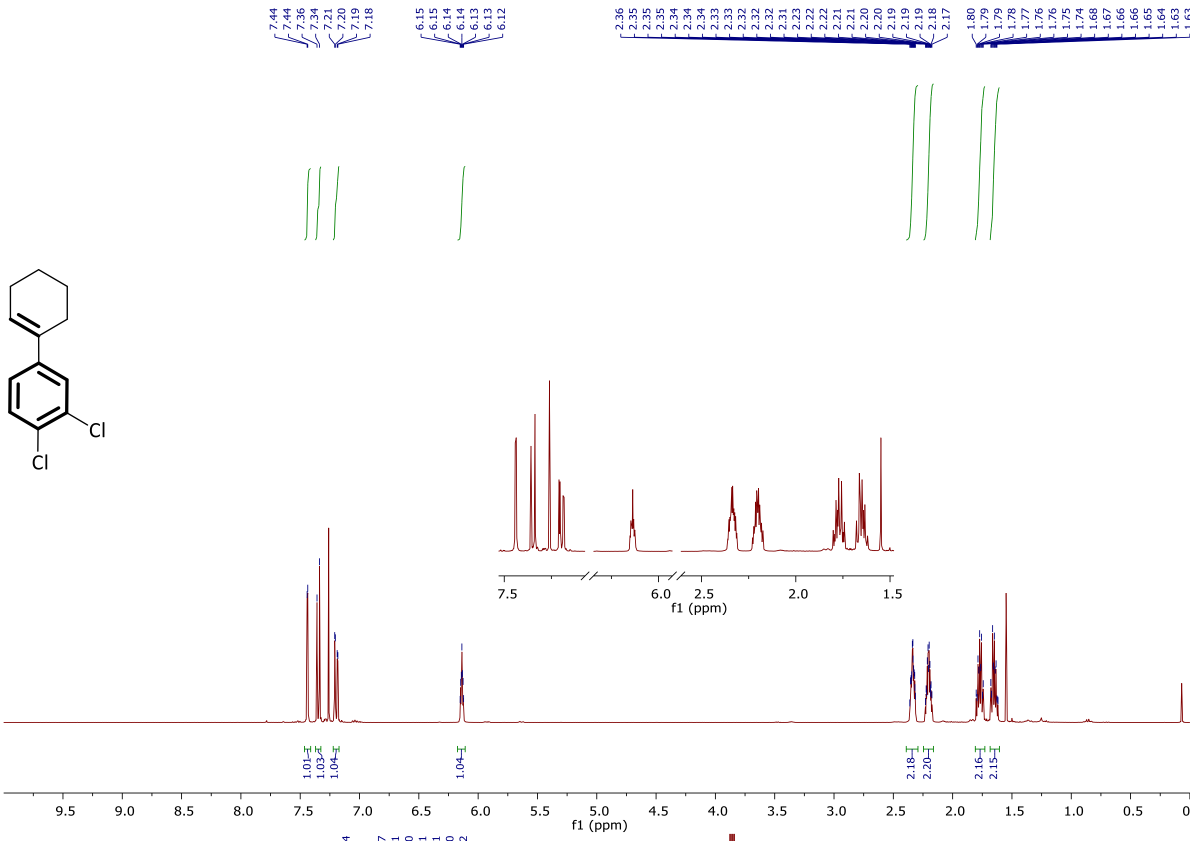

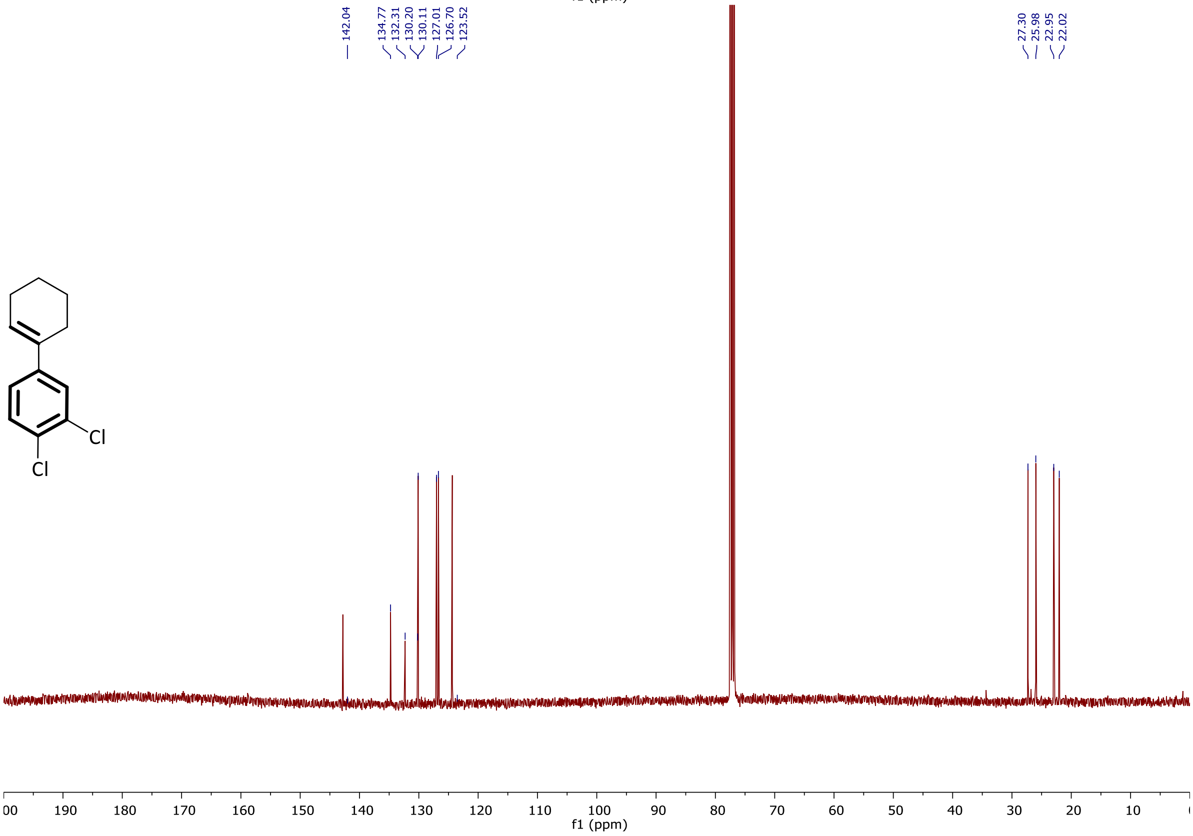

4'-Fluoro-2,3,4,5-tetrahydro-1,1'-biphenyl (4e)

^{1}H NMR (400 MHz, $CDCl_3$)

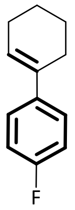

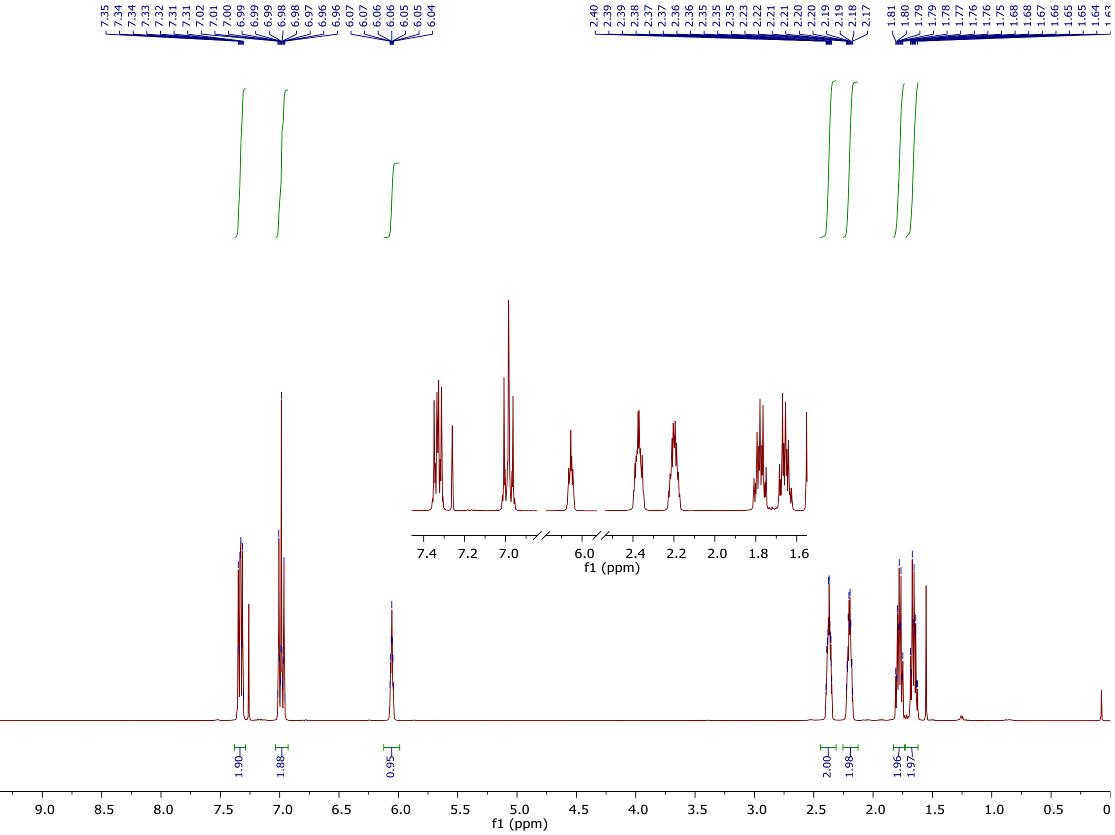

4'-Fluoro-2,3,4,5-tetrahydro-1,1'-biphenyl (4f)

^{1}H NMR (400 MHz, $CDCl_3$)

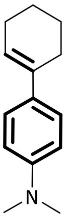

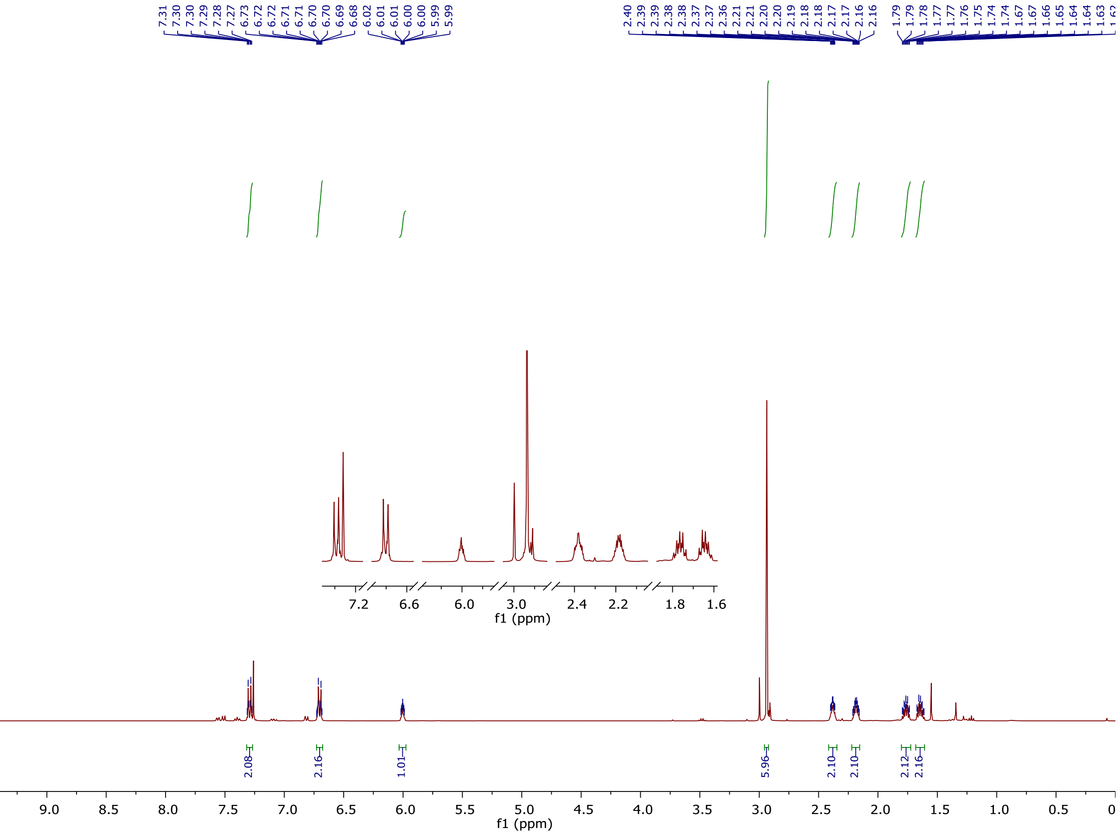

4-Phenyl-3,6-dihydro-2*H*-pyran (4g)

^{1}H NMR (400 MHz, $CDCl_3$)

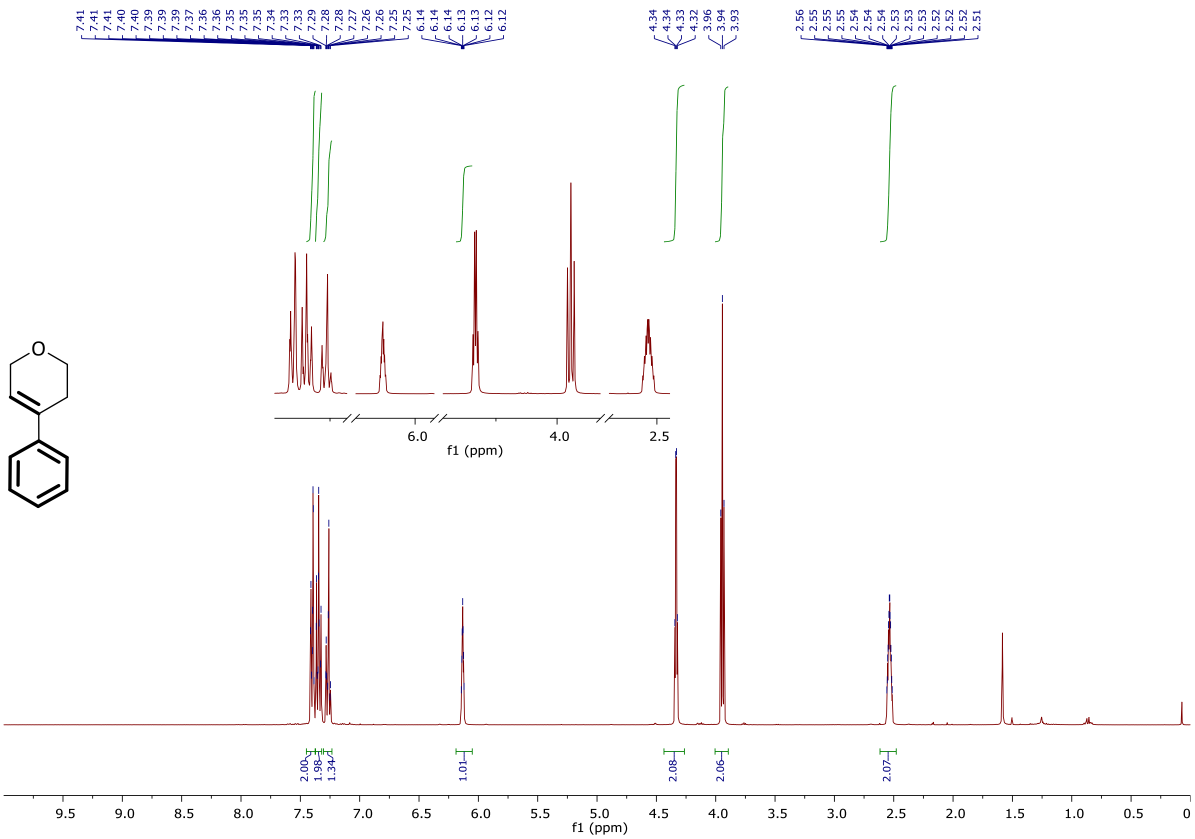

4-(3,5-Bis(trifluoromethyl)phenyl)-3,6-dihydro-2H-pyran (4h)

^{1}H NMR (400 MHz, $CDCl_3$) and **^{13}C NMR** (101 MHz, $CDCl_3$)

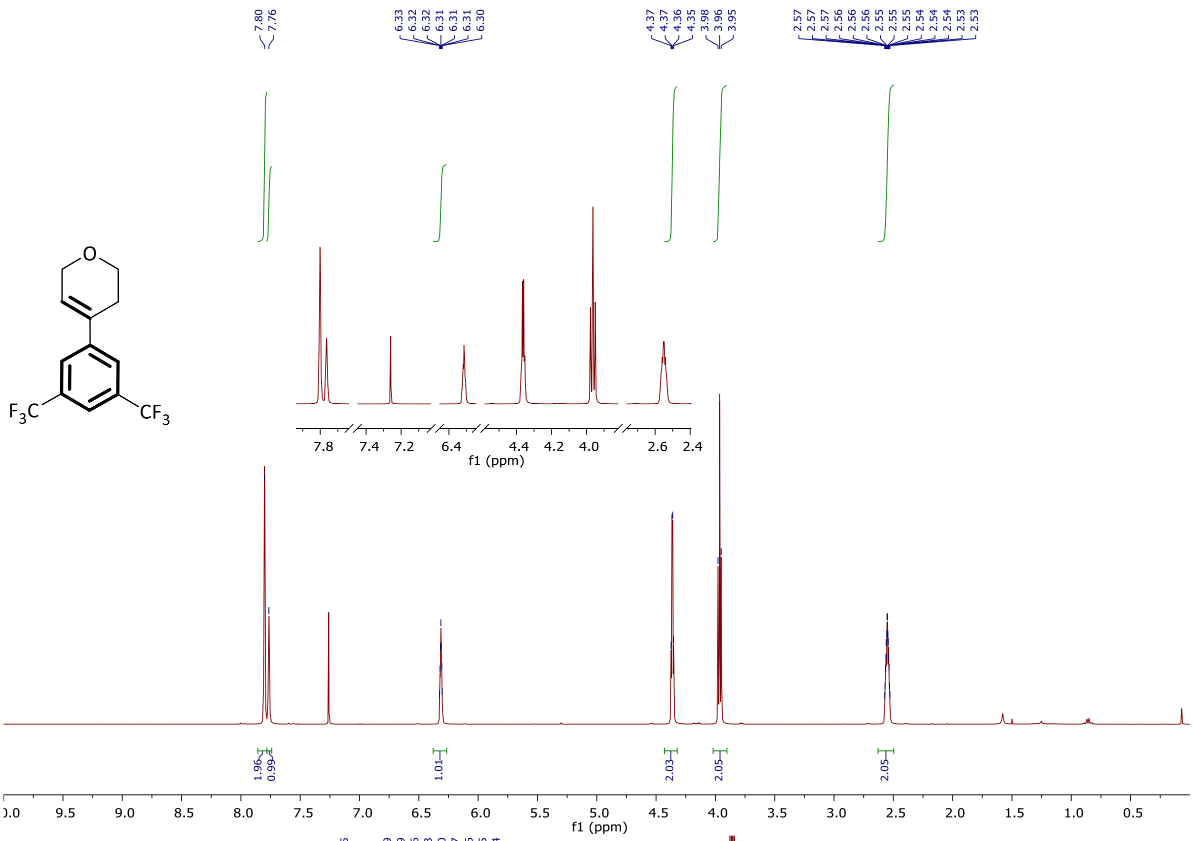

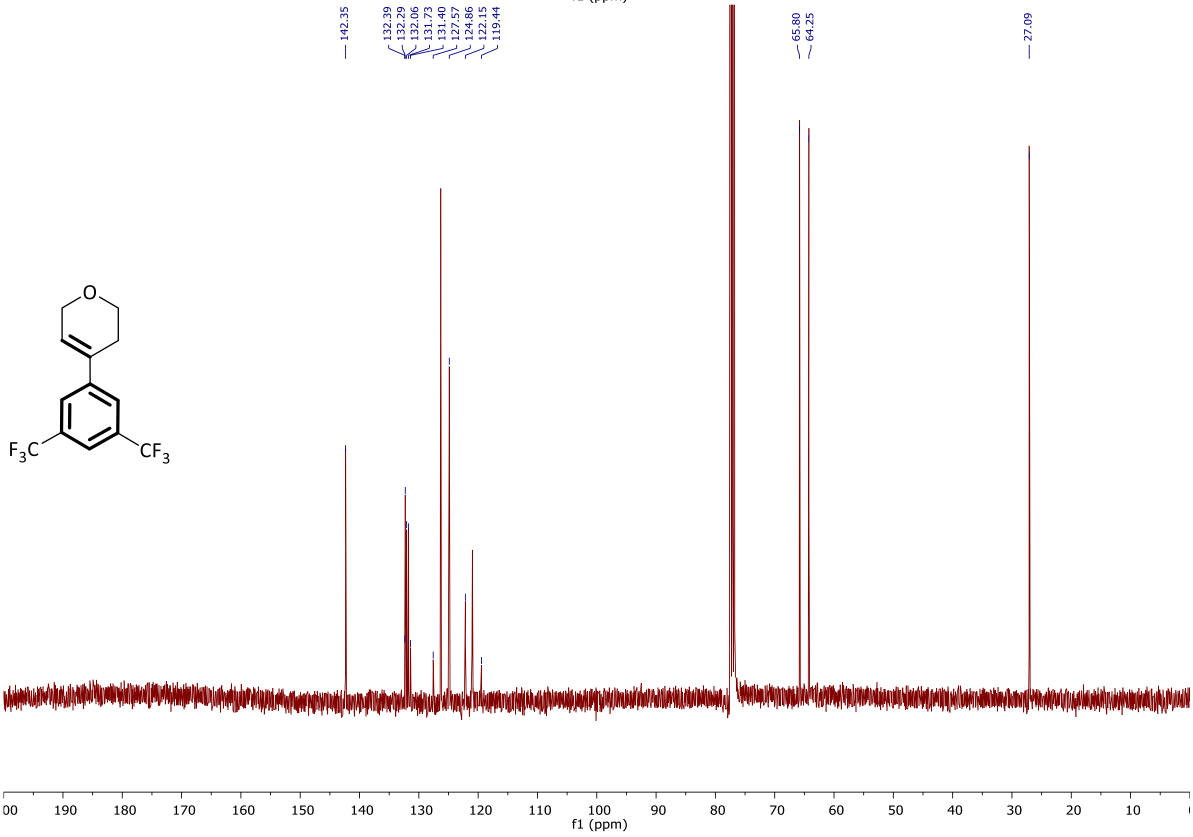

4-(3,5-Dimethylphenyl)-3,6-dihydro-2H-pyran (4i)

^{1}H NMR (400 MHz, $CDCl_3$) and **^{13}C NMR** (101 MHz, $CDCl_3$)

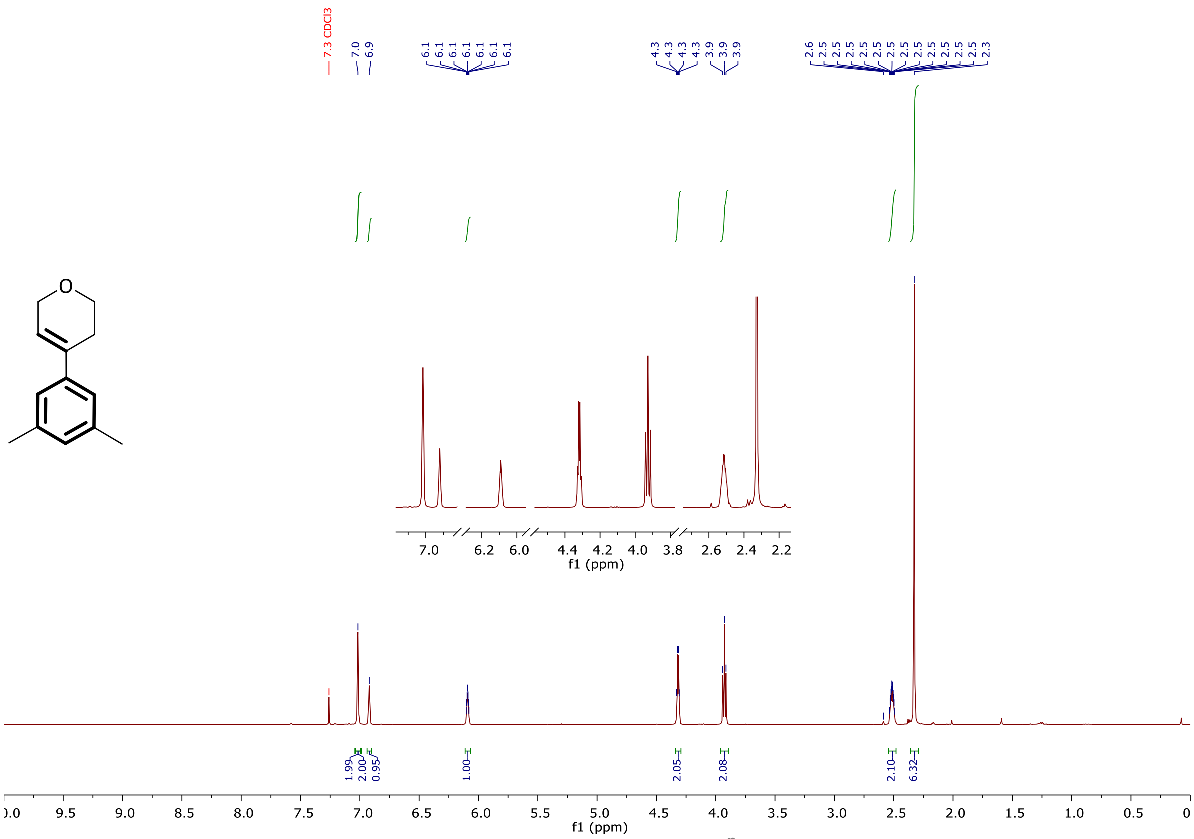

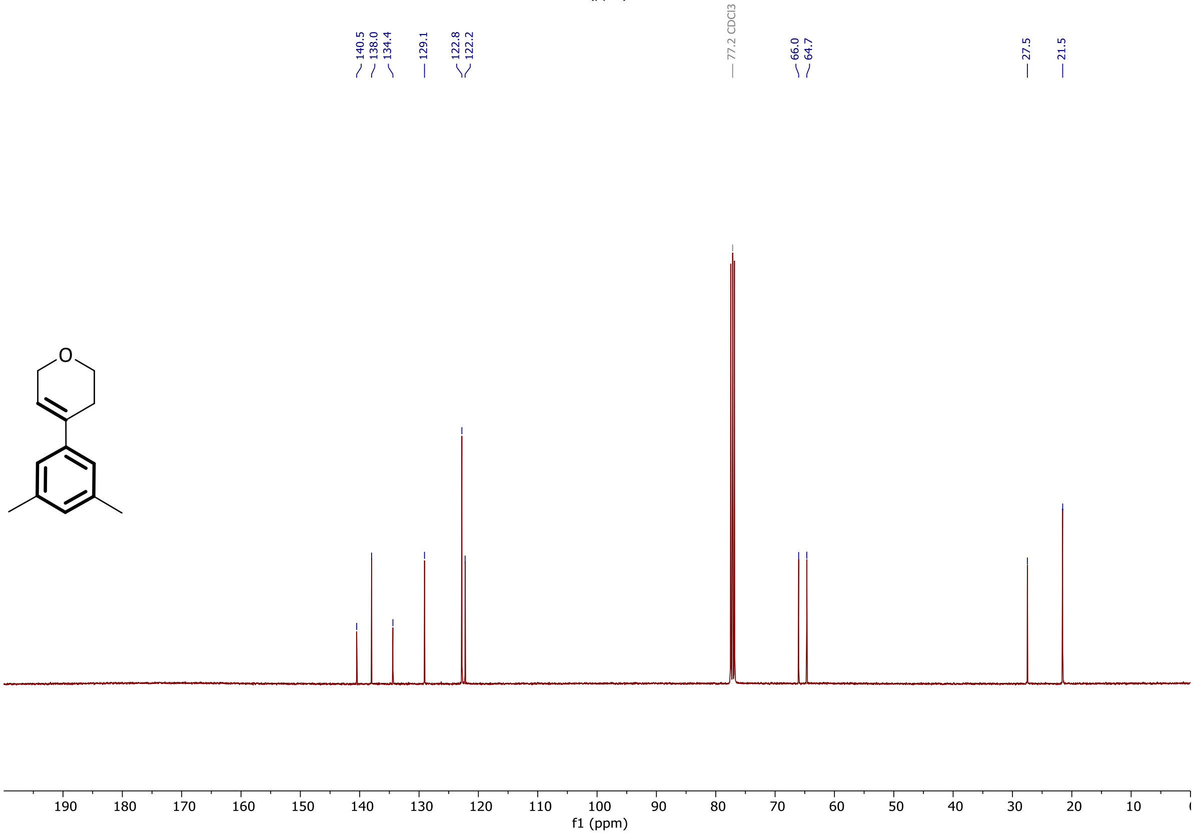

4-(Benzo[*b*]thiophen-5-yl)-3,6-dihydro-2*H*-pyran (4j)

^{1}H NMR (400 MHz, $CDCl_3$) and **^{13}C NMR** (101 MHz, $CDCl_3$)

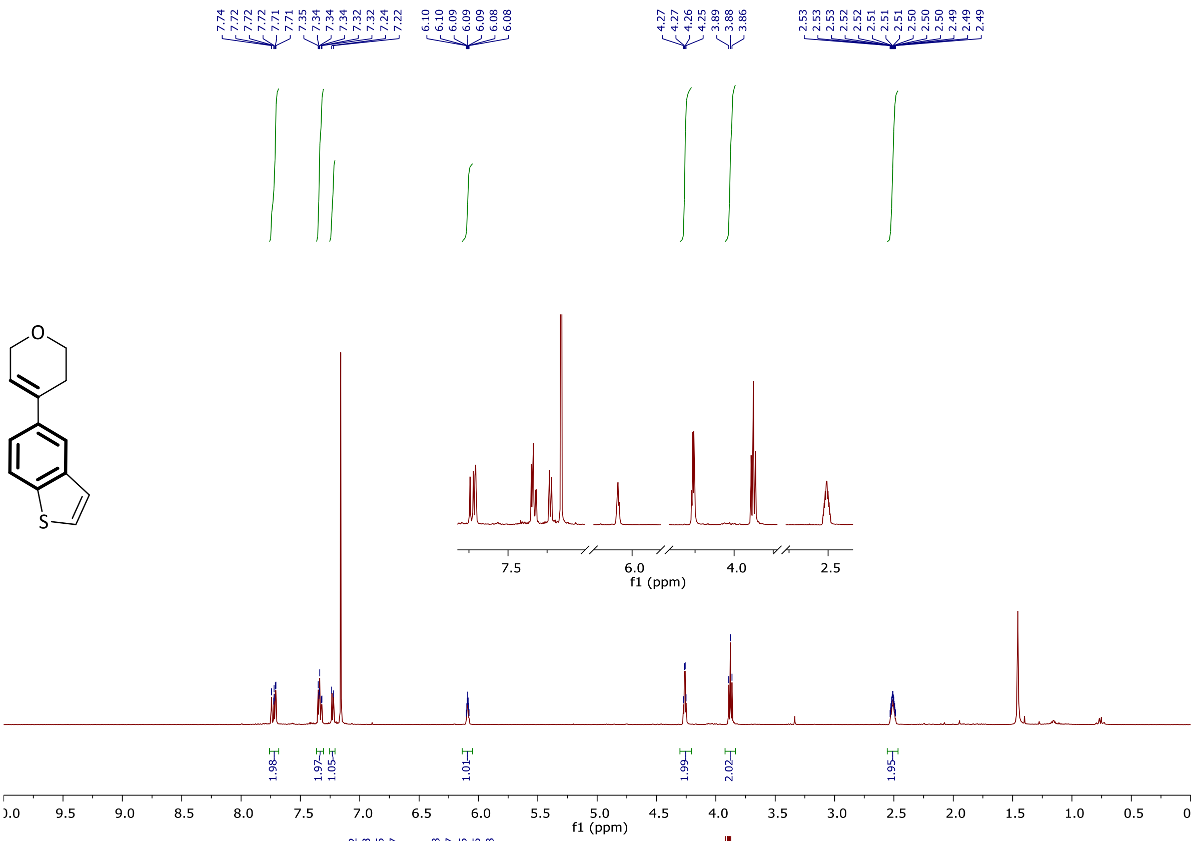

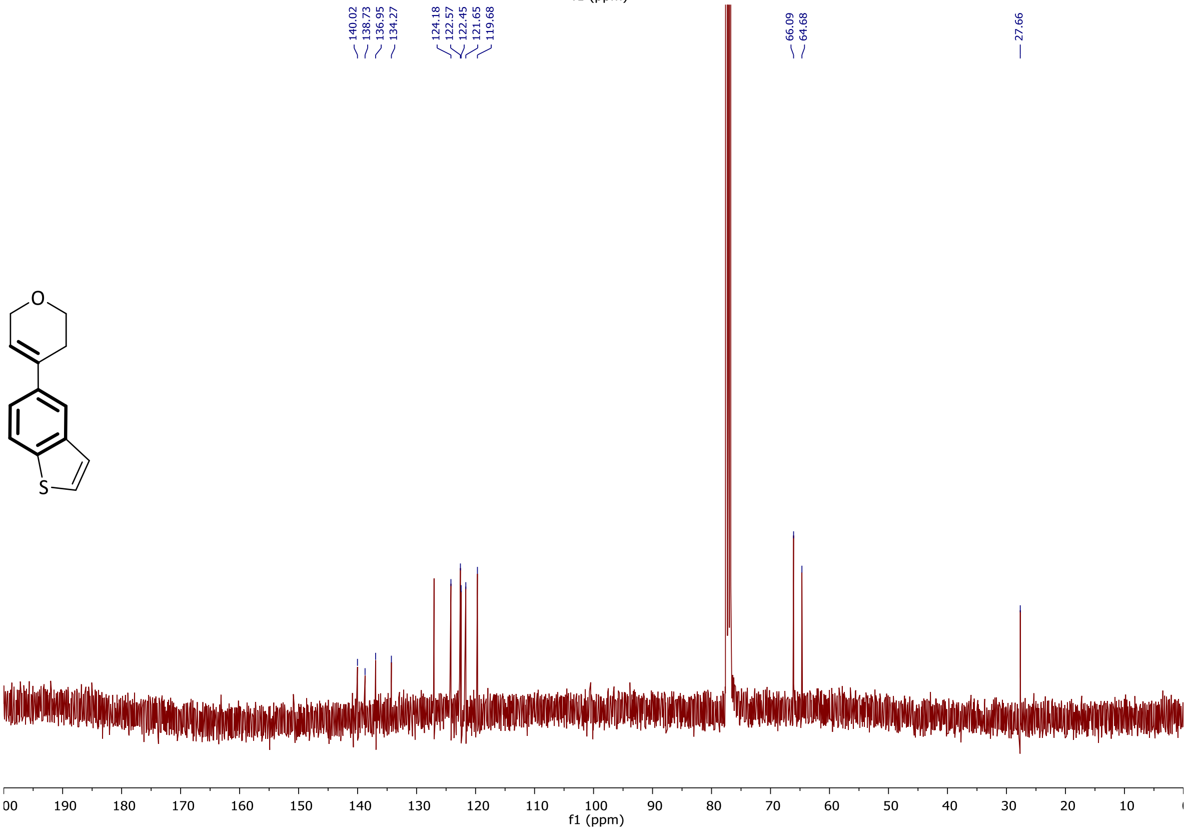

5-(3,6-Dihydro-2*H*-pyran-4-yl)-2,2-difluorobenzo[*d*][1,3]dioxole (4k)

^{1}H NMR (400 MHz, $CDCl_3$) and **^{13}C NMR** (101 MHz, $CDCl_3$)

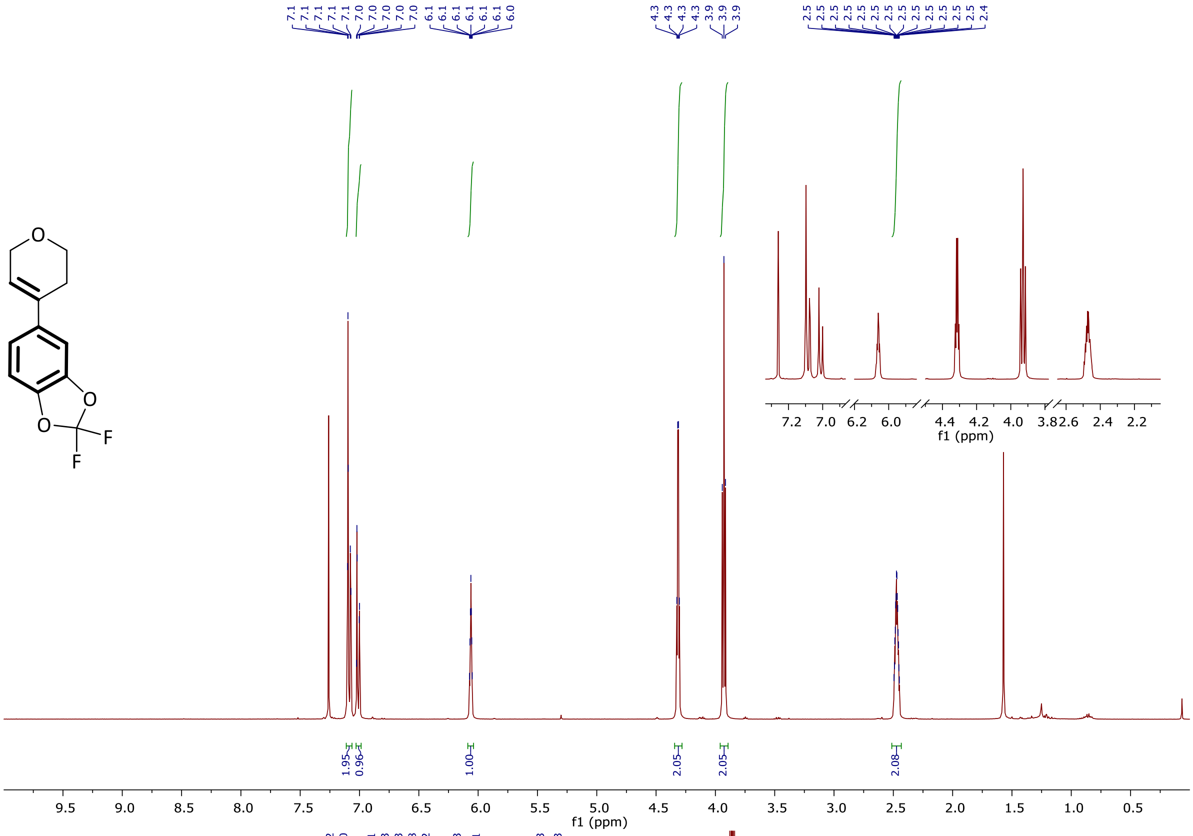

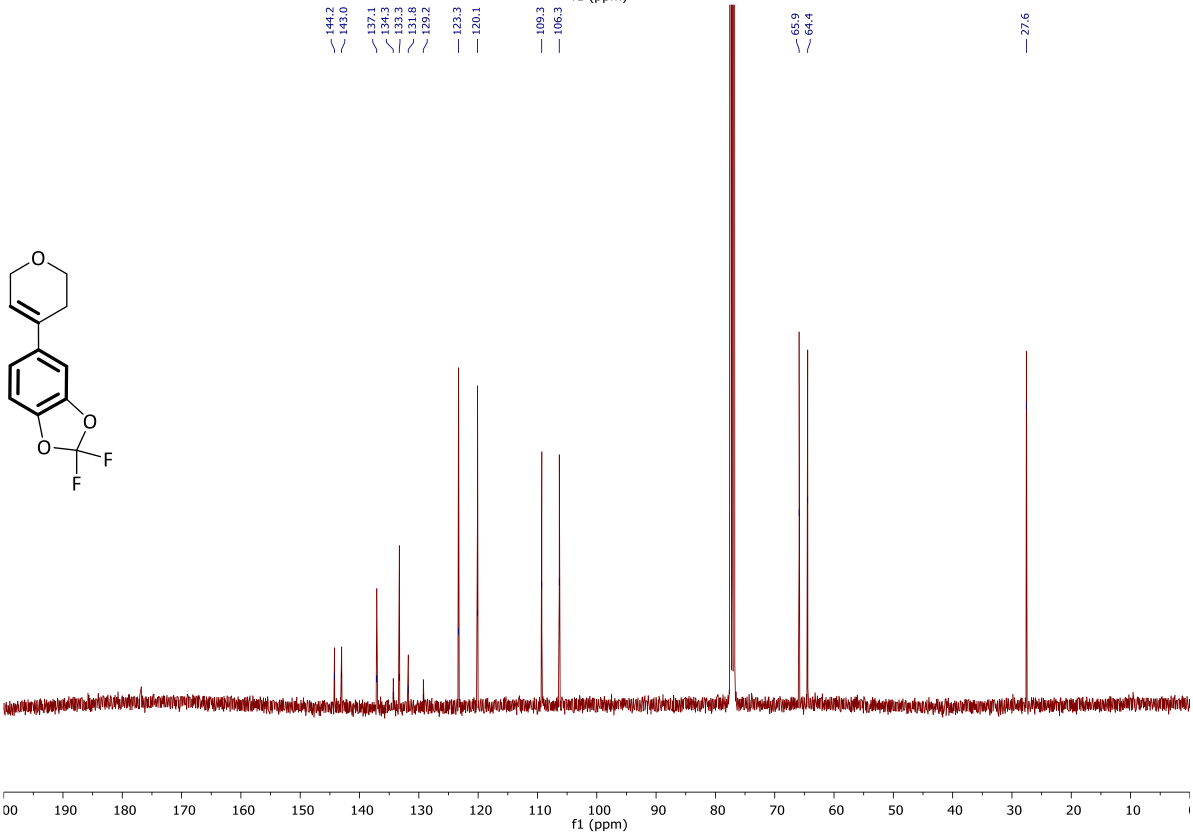

4-(4-Methoxyphenyl)-3,6-dihydro-2*H*-thiopyran (4l)

^{1}H NMR (400 MHz, $CDCl_3$) and **^{13}C NMR** (101 MHz, $CDCl_3$)

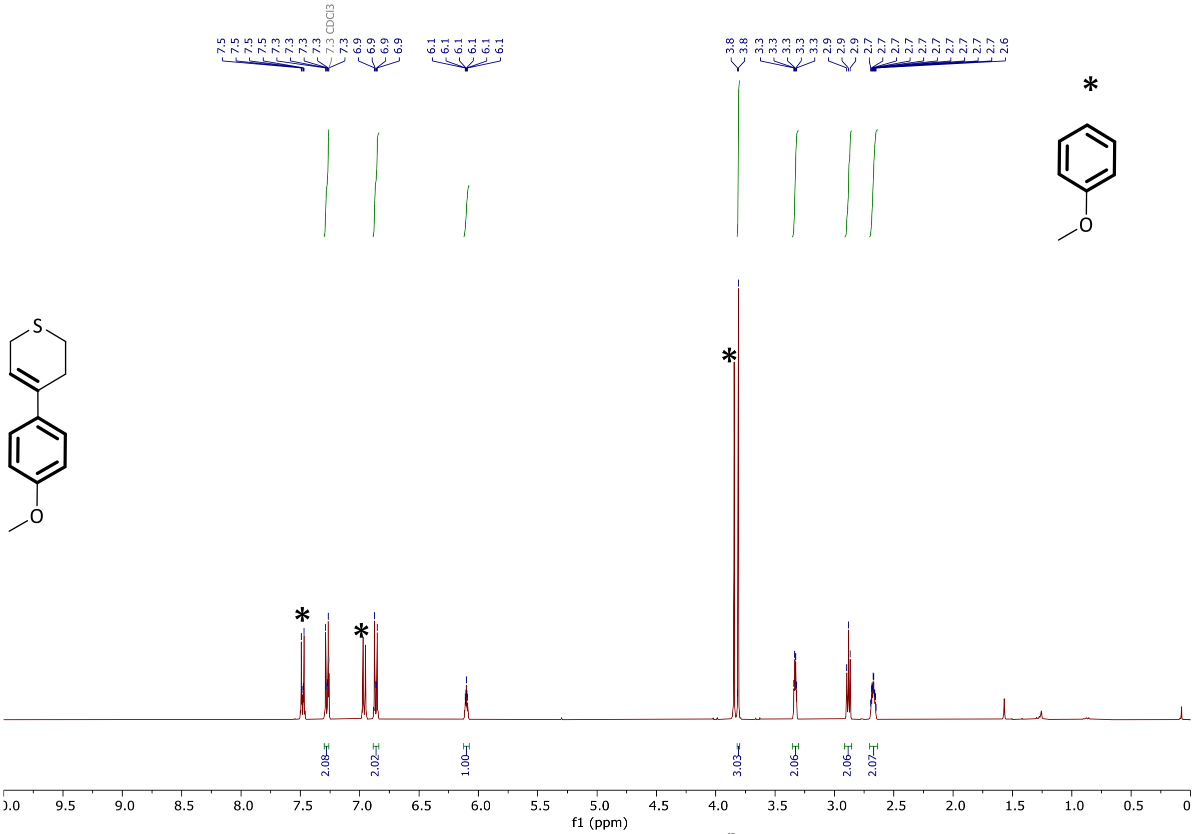

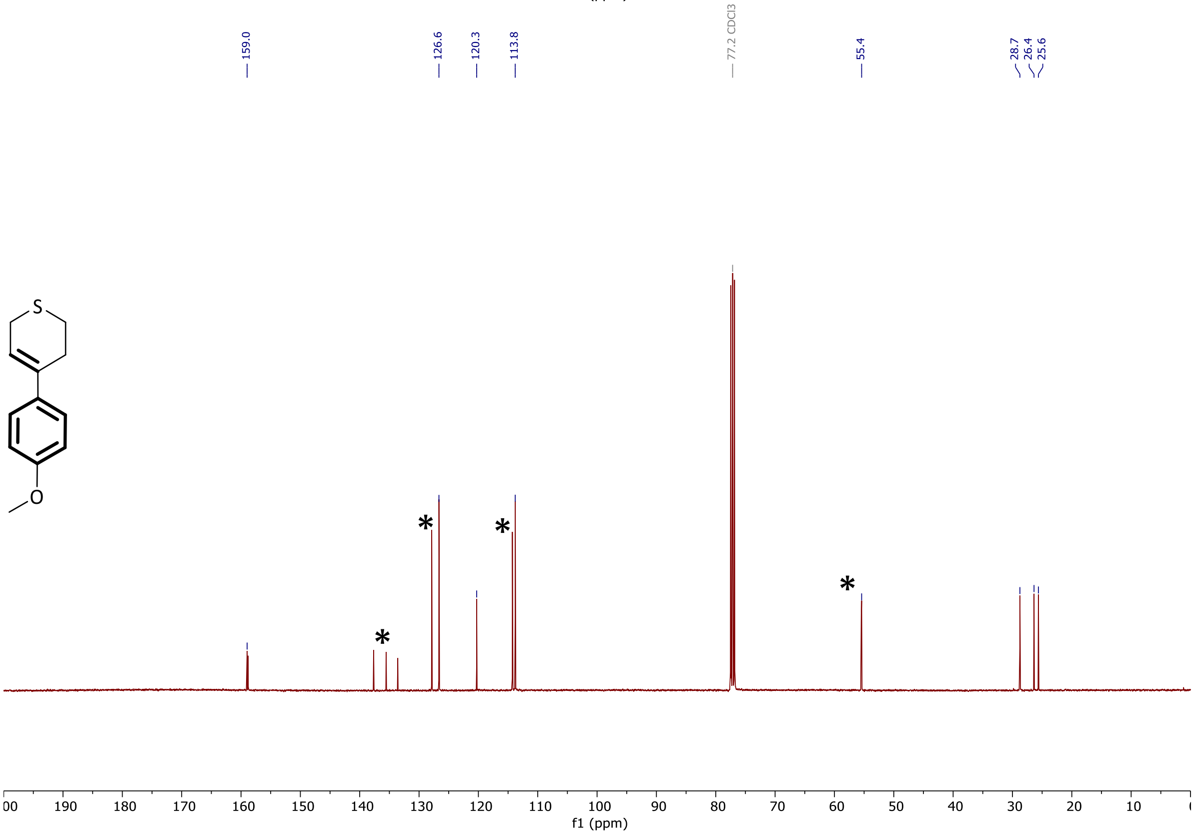

***tert*-Butyl 4-(4-methoxyphenyl)-3,6-dihydropyridine-1(2*H*)-carboxylate (4m)**

^{1}H NMR (400 MHz, $CDCl_3$)

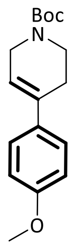

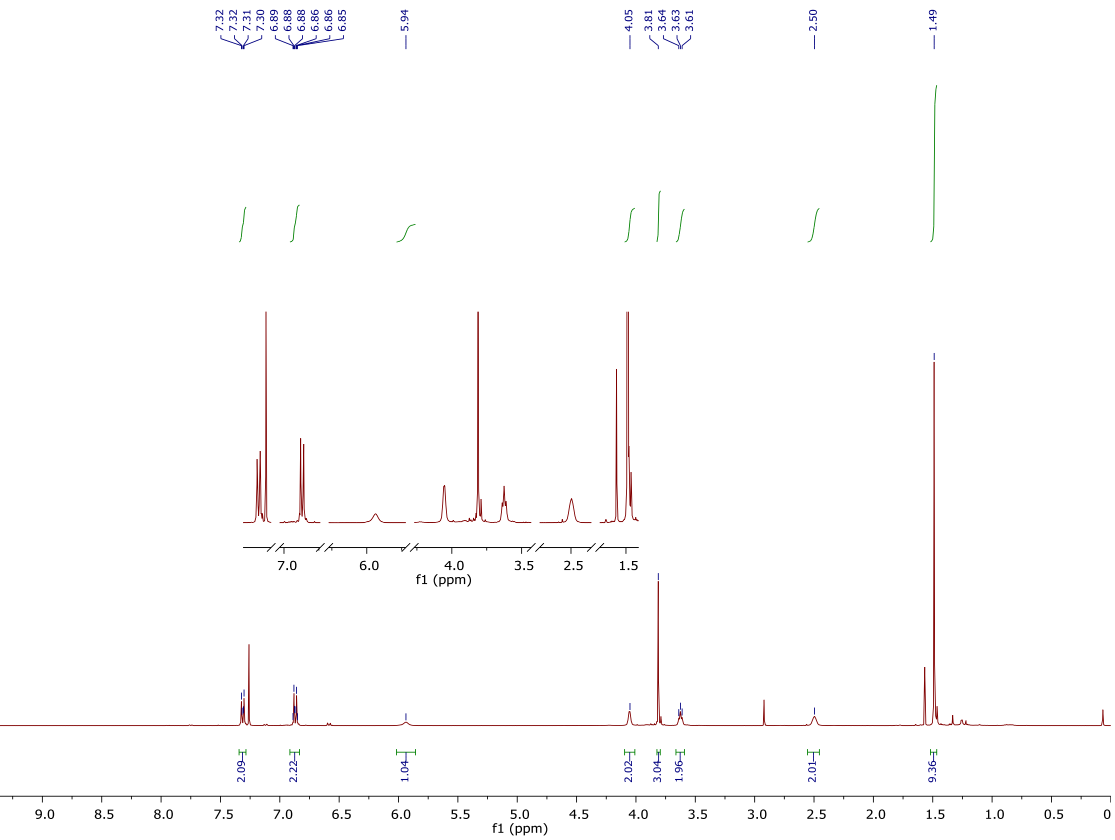

***tert*-Butyl 4-(4-fluorophenyl)-3,6-dihydropyridine-1(2*H*)-carboxylate (4n)**

^{1}H NMR (400 MHz, $CDCl_3$)

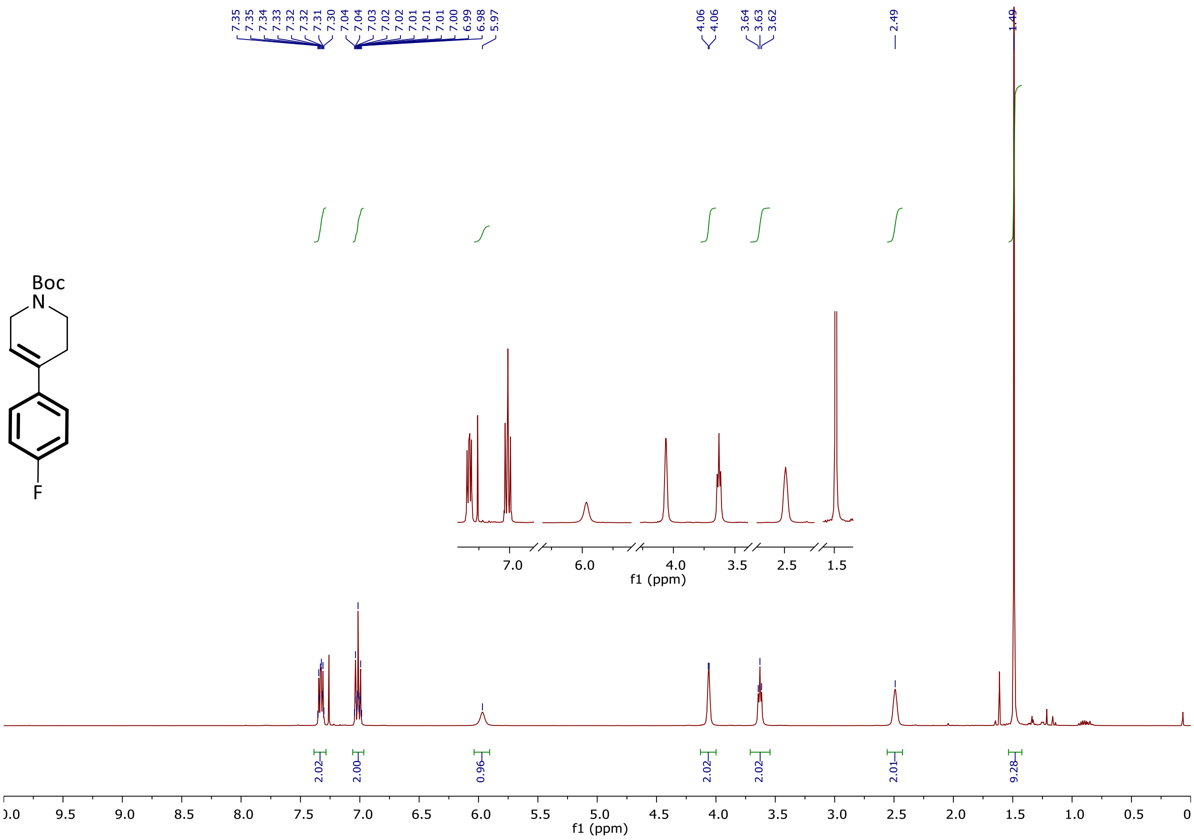

***tert*-Butyl 4-(4-(trifluoromethyl)phenyl)-3,6-dihydropyridine-1(2*H*)-carboxylate (4o)**

^{1}H NMR (400 MHz, $CDCl_3$)

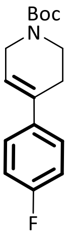

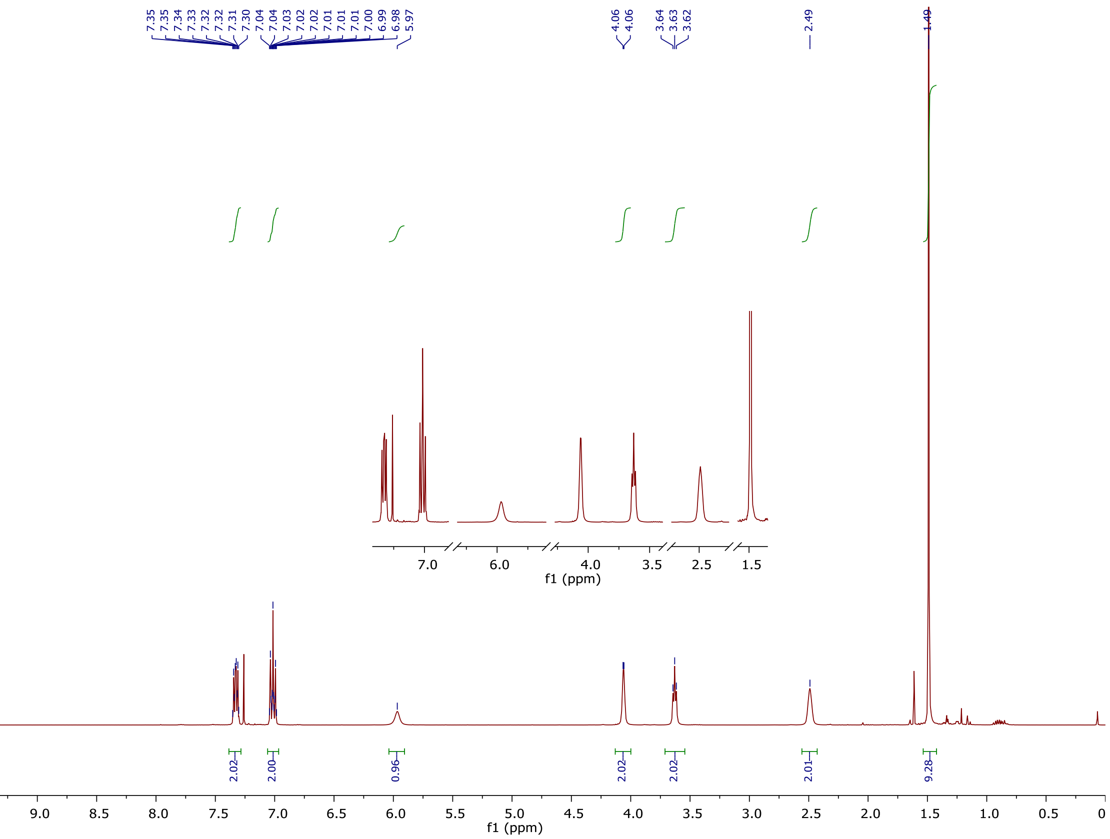

8-(4-Methoxyphenyl)-1,4-dioxaspiro[4.5]dec-7-ene (4p)

^{1}H NMR (400 MHz, $CDCl_3$)

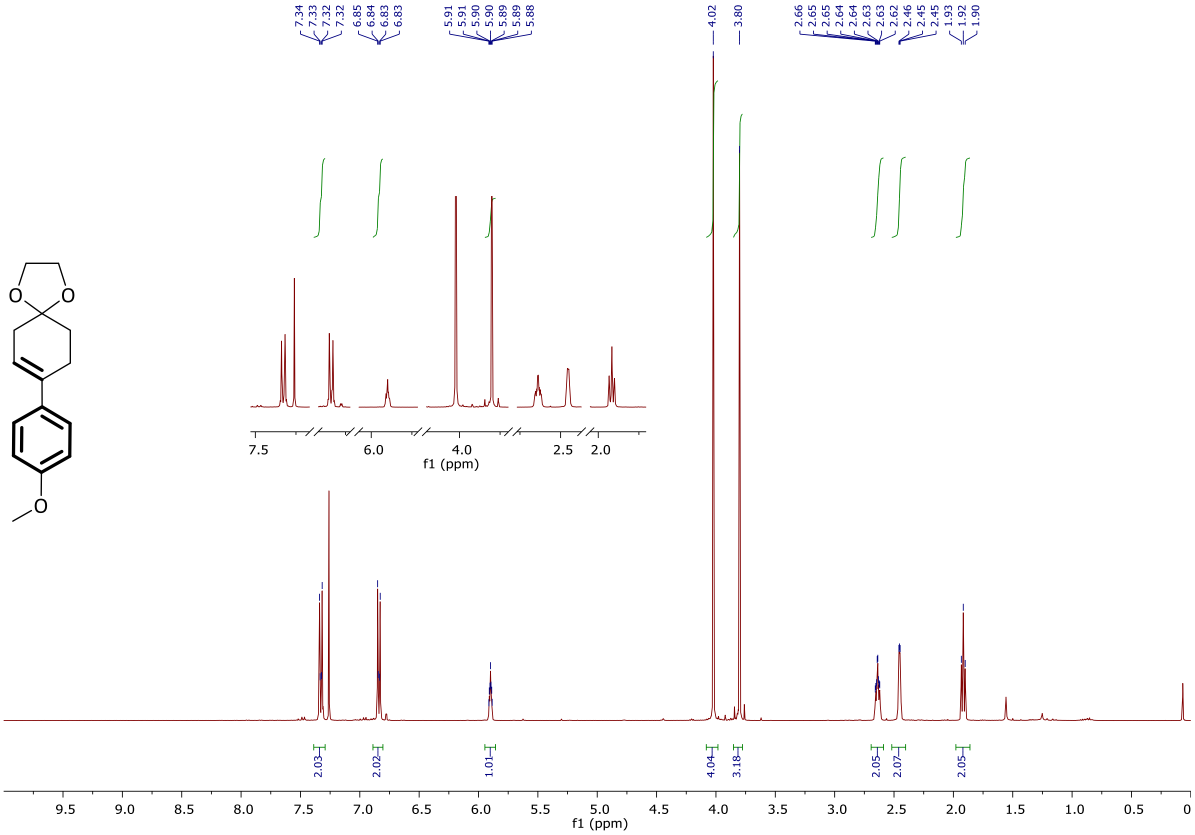

8-(3,5-Difluorophenyl)-1,4-dioxaspiro[4.5]dec-7-ene (4q)

^{1}H NMR (400 MHz, $CDCl_3$) and **^{13}C NMR** (101 MHz, $CDCl_3$)

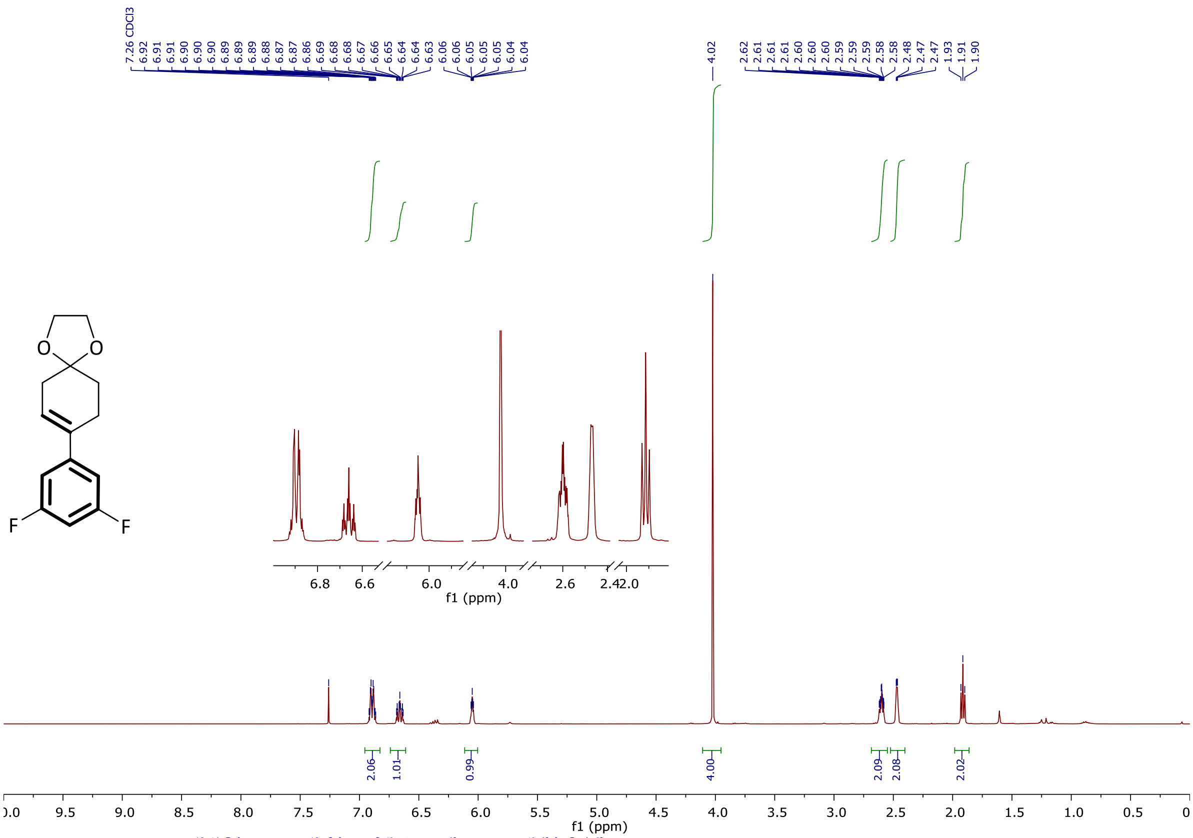

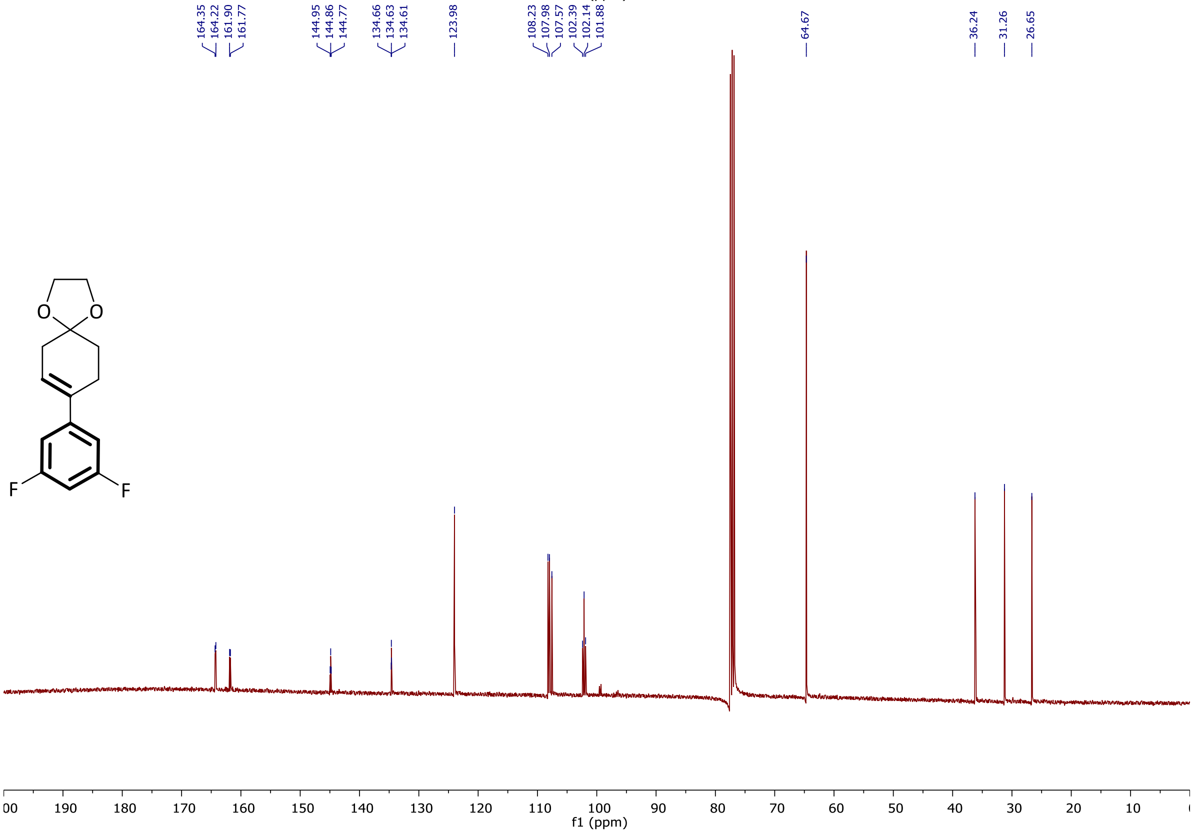

8-(4-Fluorophenyl)-1,4-dioxaspiro[4.5]dec-7-ene (4r)

^{1}H NMR (400 MHz, $CDCl_3$) and **^{13}C NMR** (101 MHz, $CDCl_3$)

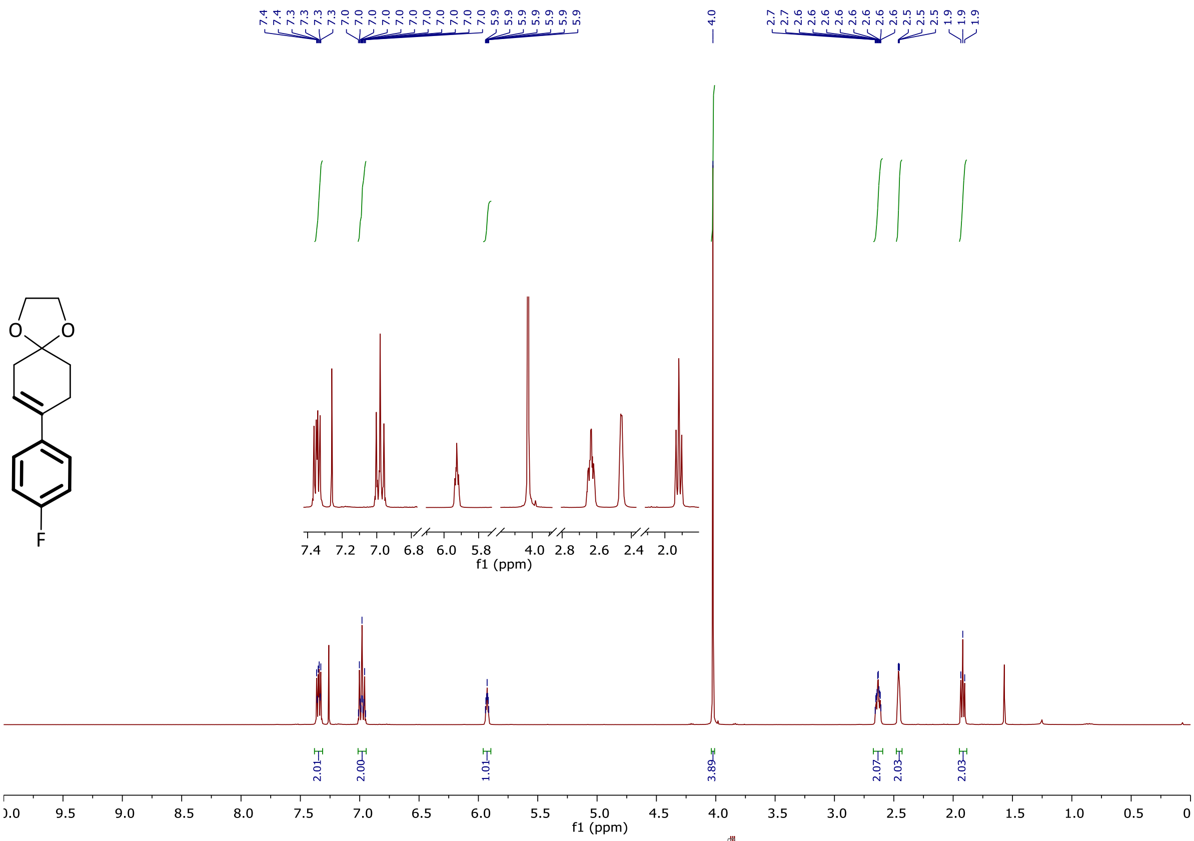

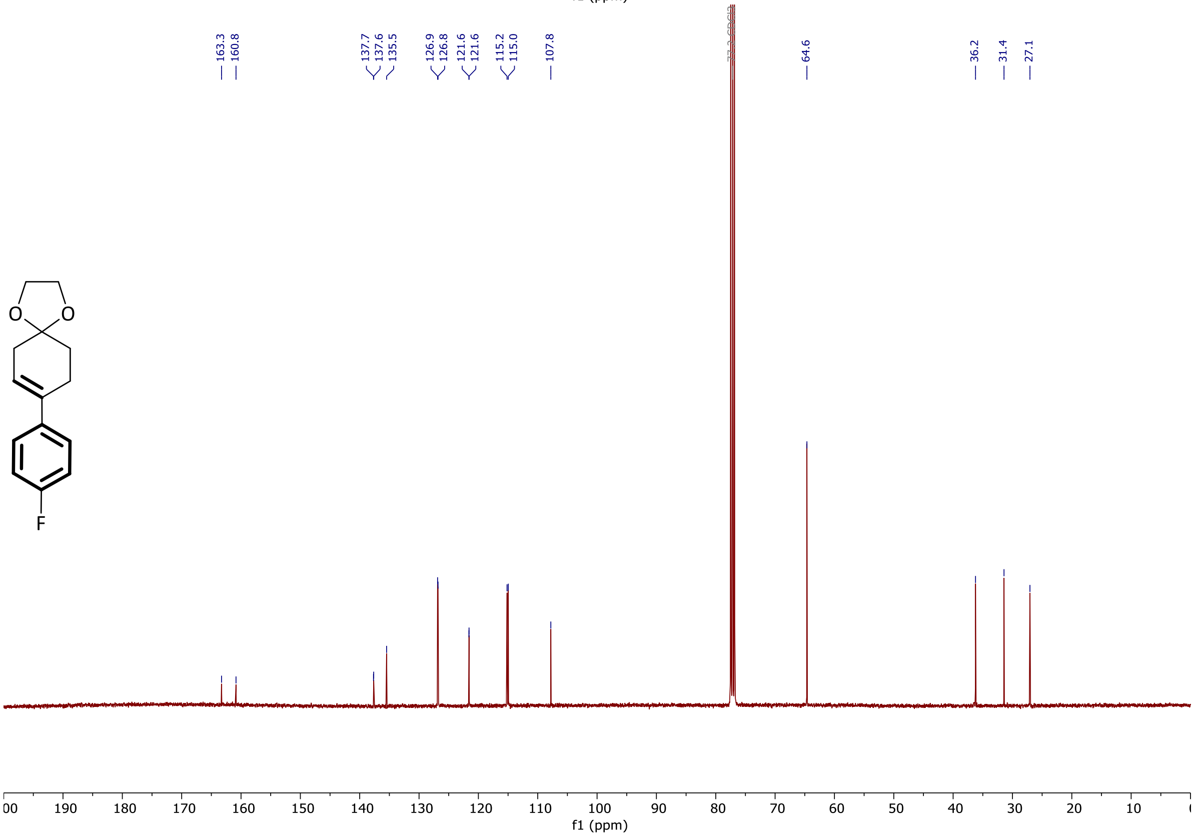

***tert*-Butyl(((*3S*,*8R*,*9S*,*10R*,*13S*,*14S*)-17-(4-fluorophenyl)-10,13-dimethyl-2,3,4,7,8,9,10,11,12,13,14,15-dodecahydro-1*H*-cyclopenta[*a*]phenanthren-3-yl)oxy)dimethylsilane (5a)**

^{1}H NMR (400 MHz, $CDCl_3$) and **^{13}C NMR** (101 MHz, $CDCl_3$)

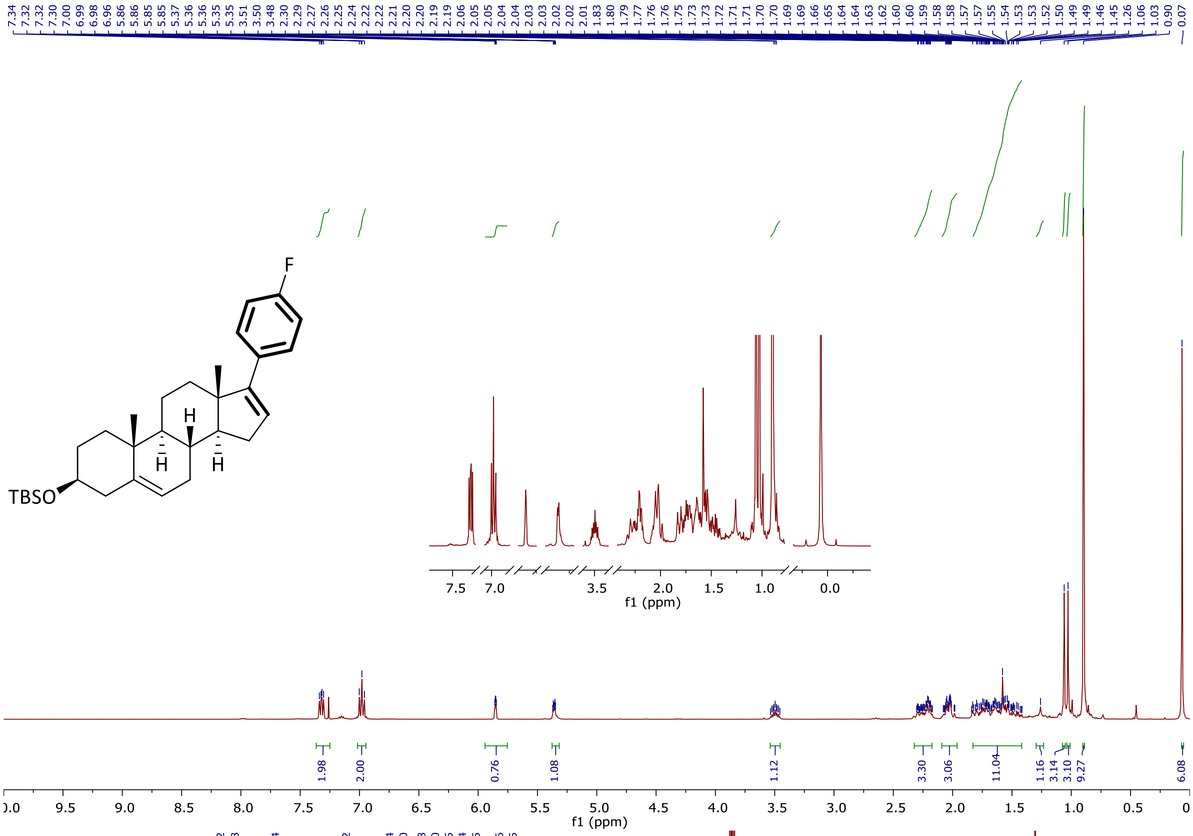

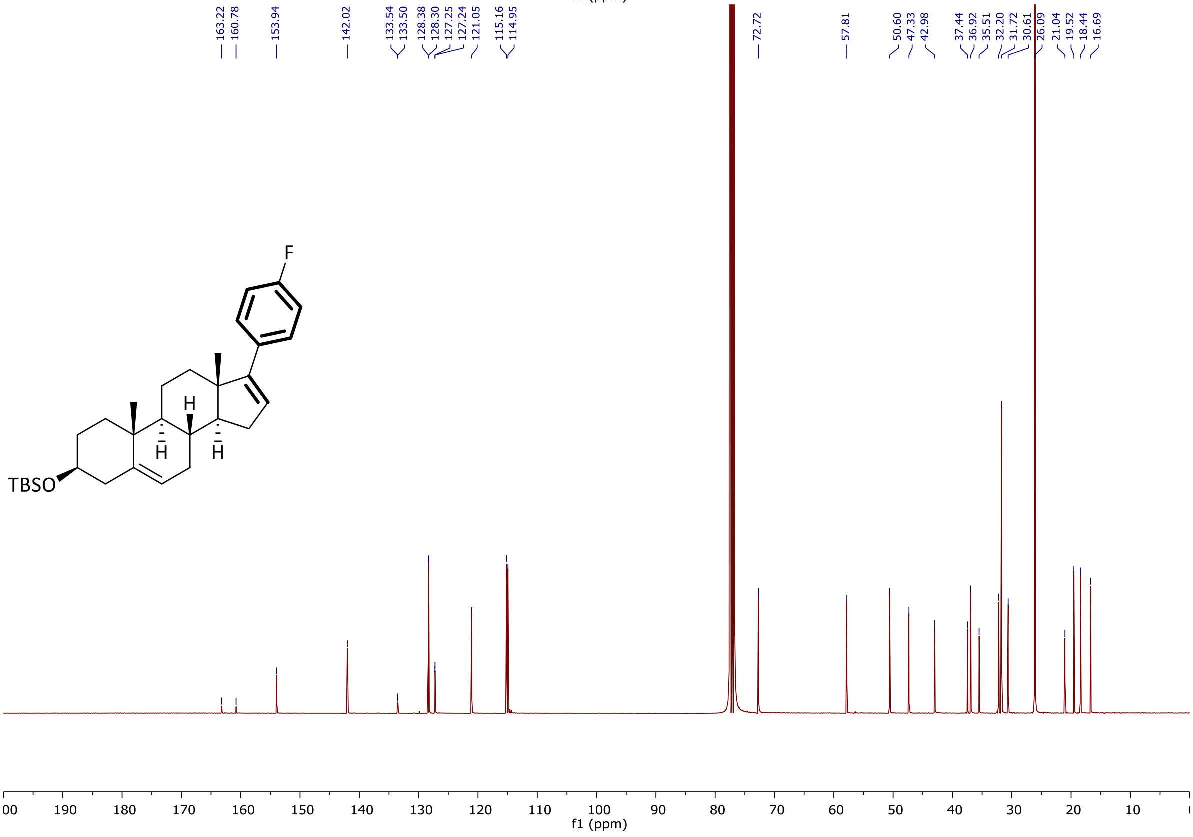

***tert*-Butyl(((*3S*,*8R*,*9S*,*10R*,*13S*,*14S*)-10,13-dimethyl-17-(4-(trifluoromethoxy)phenyl)-2,3,4,7,8,9,10,11,12,13,14,15-dodecahydro-1*H*-cyclopenta[*a*]phenanthren-3-yl)oxy)dimethylsilane (5b)**

^{1}H NMR (400 MHz, $CDCl_3$) and **^{13}C NMR** (101 MHz, $CDCl_3$)

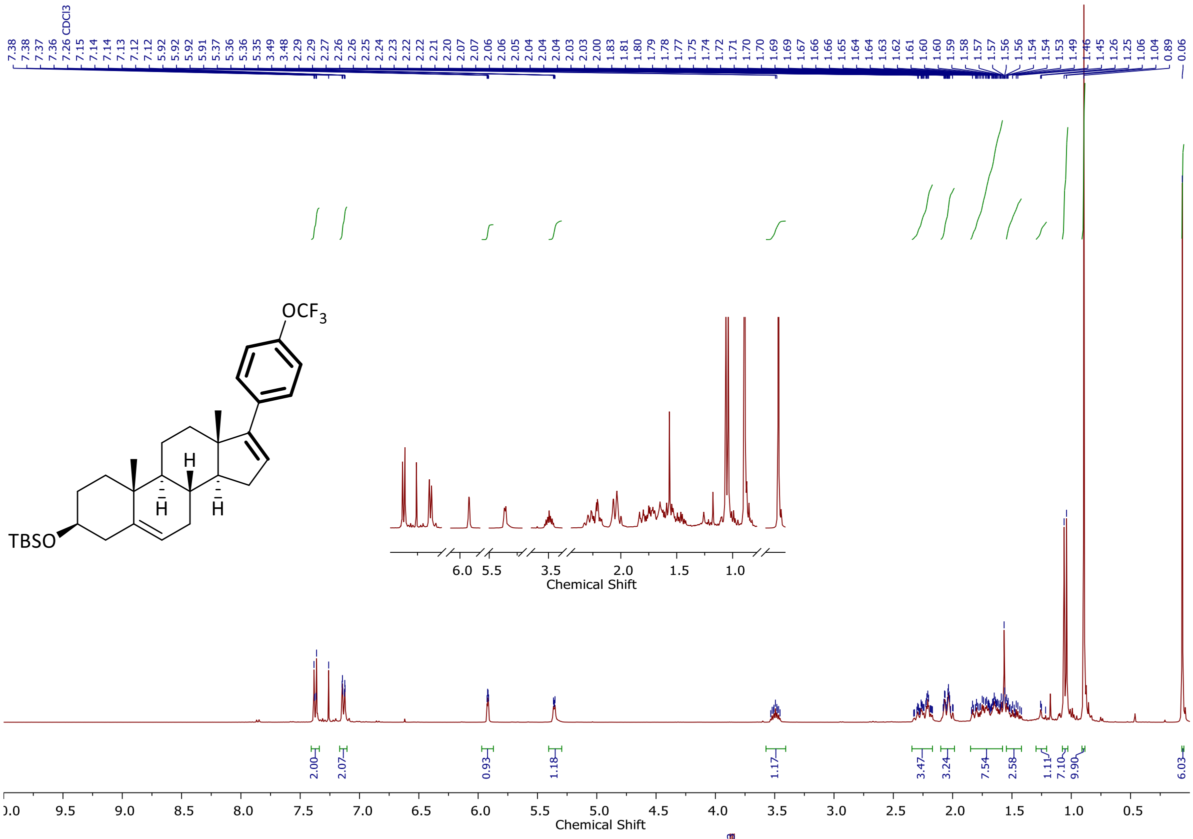

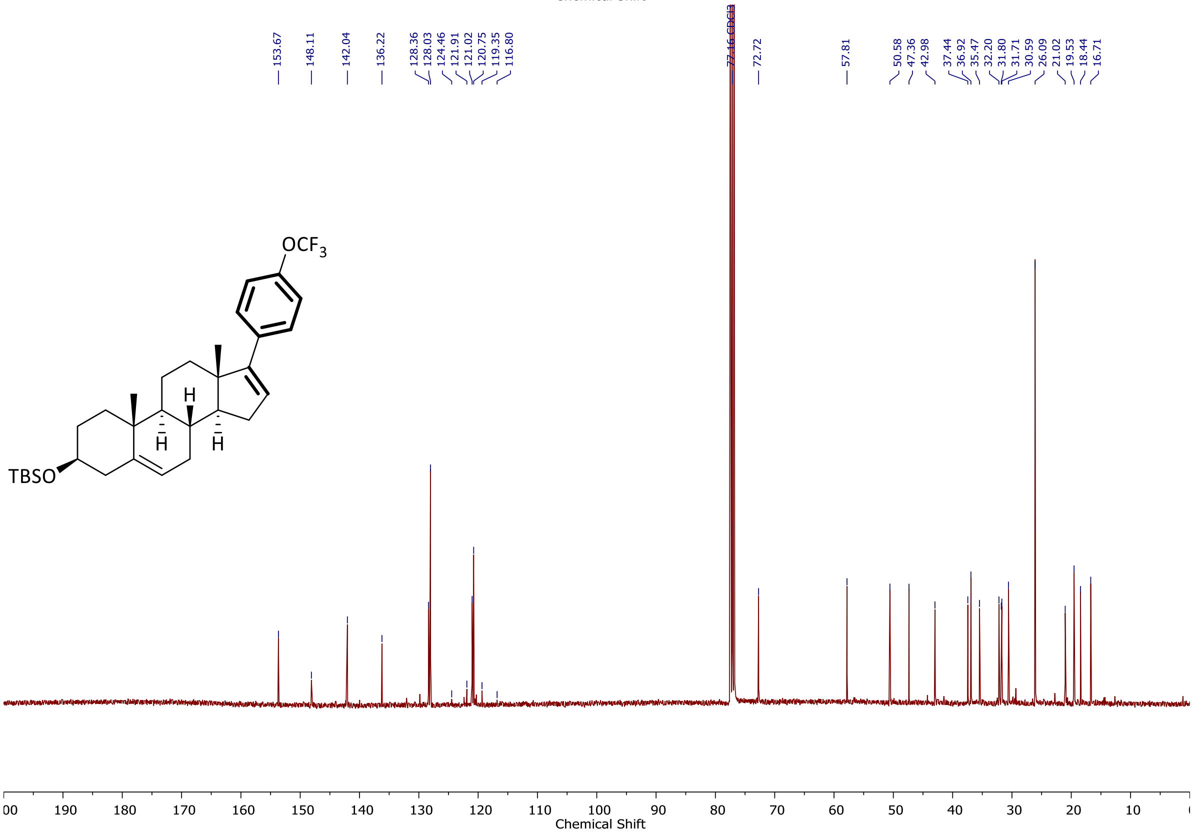

(*E*)-5-Styrylbenzene-1,3-diol (pinosylvin) (5c)

^{1}H NMR (400 MHz, $CDCl_3$)

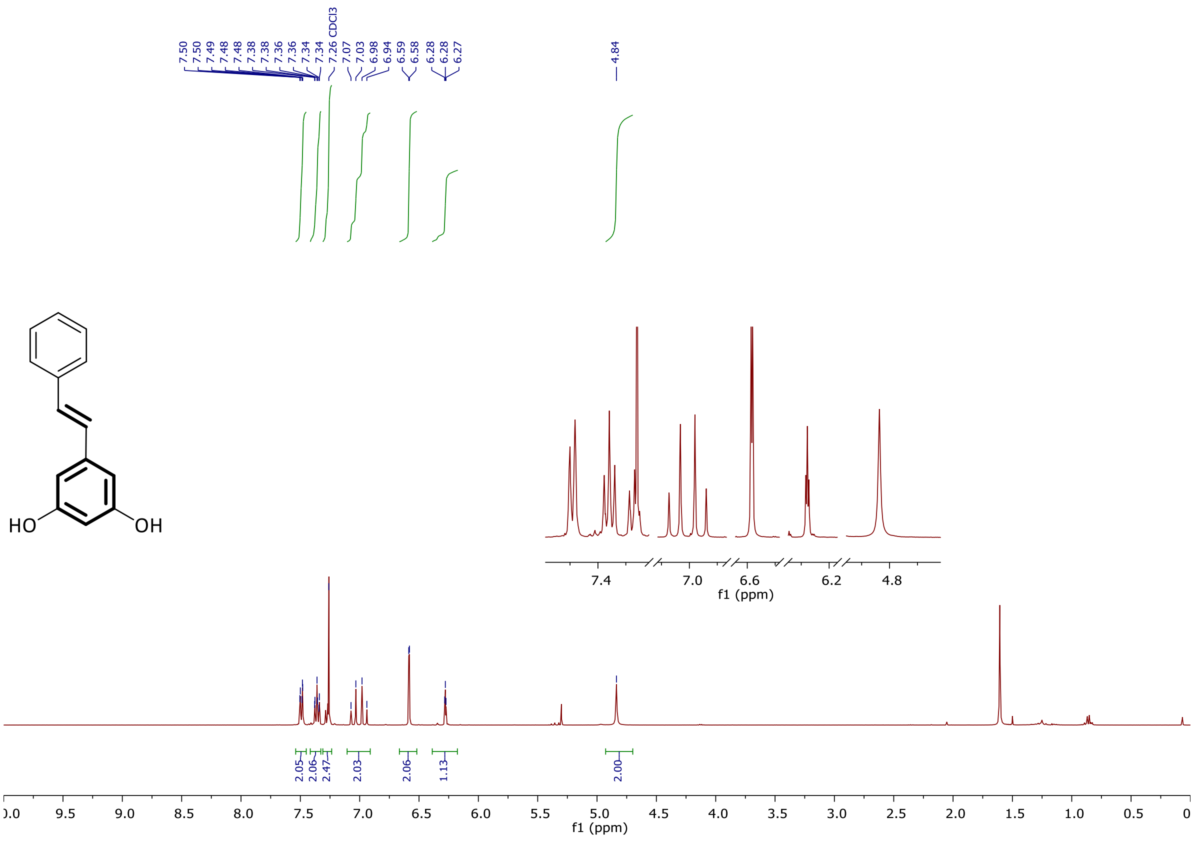

4. Cyclic Voltammetry

The oxidation potentials were determined in acetonitrile on a CH Instruments 630E electrochemical analyzer using a 2 mm diameter platinum working electrode, a platinum wire counter electrode and an Ag wire pseudo-reference electrode applying a scan rate of 0.1 V/s. Cyclic voltammetry measurements were performed in acetonitrile containing 0.1 M NBu_4ClO_4 with the ATB salts (**2a-b**) (c ≈ 3-4 × 10−4 M) and ferrocene (c = 3.8 × 10−4 M) as an internal standard. The $E_{1/2}(fc^+/fc$ in MeCN) = +0.382 V was used to calibrate E_p^{Ox} (in MeCN) vs. SCE.

*Table 2: Determined Oxidation potentials of ATB salts **2a-b** vs. SCE.*

ATB salt	ATB structure	E_p^{Ox} vs. SCE / V
**2a**	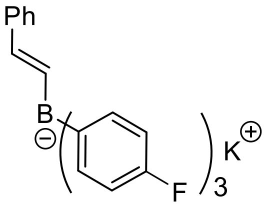	0.81
**2b**	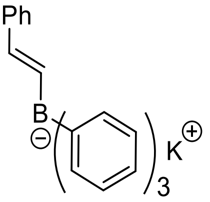	0.67
**$NaBPh_4$ reference (2c)**	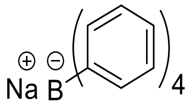	0.82

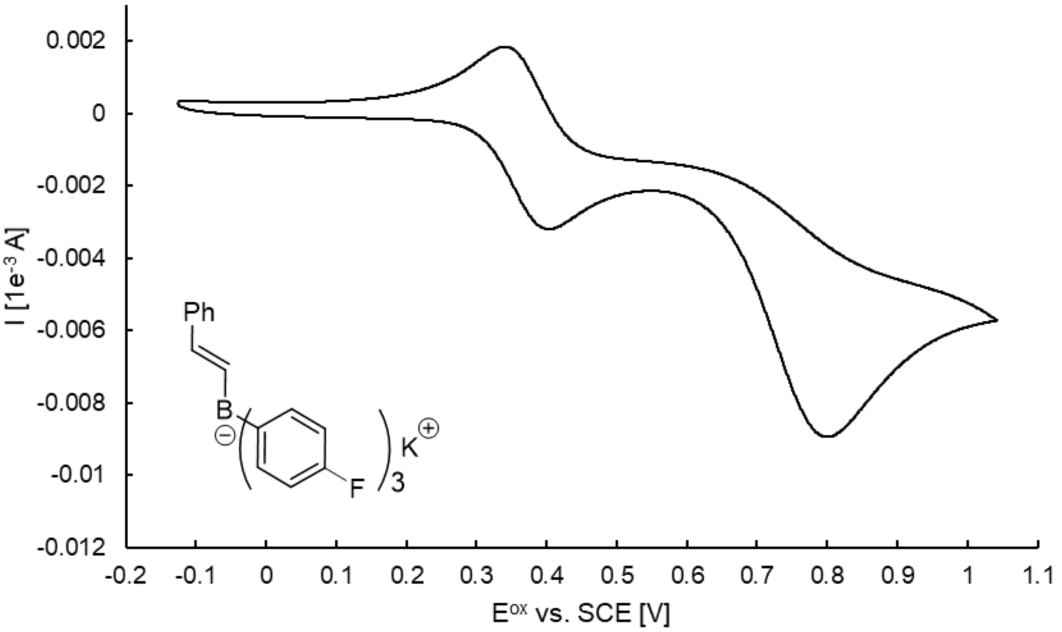

*Figure 2: Cyclic voltammogram of ATB salt **2a**.*

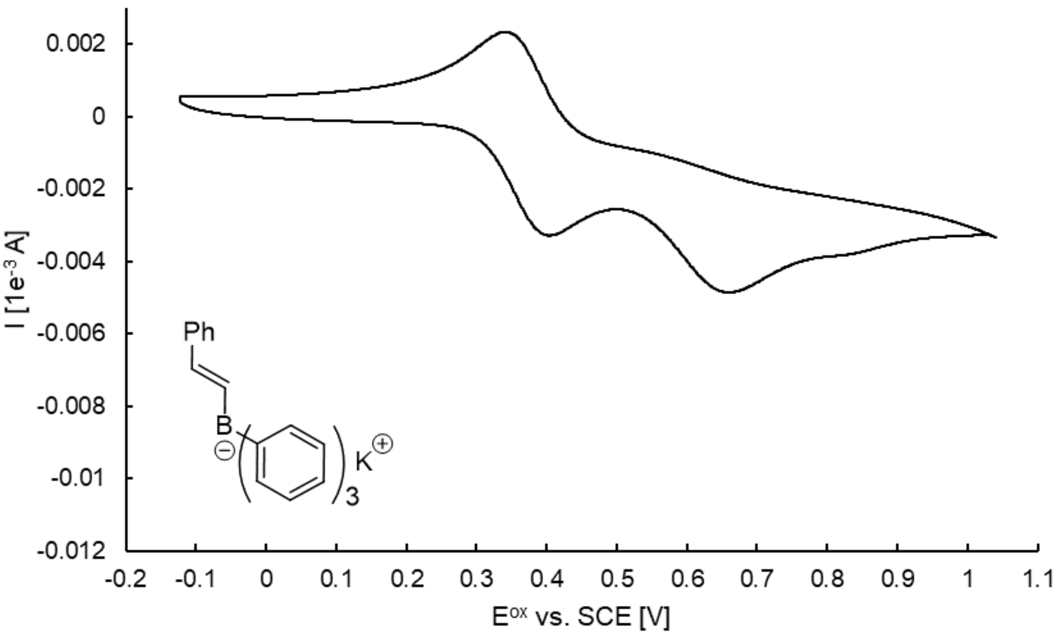

Figure 3: Cyclic voltammogram of ATB salt 2b.

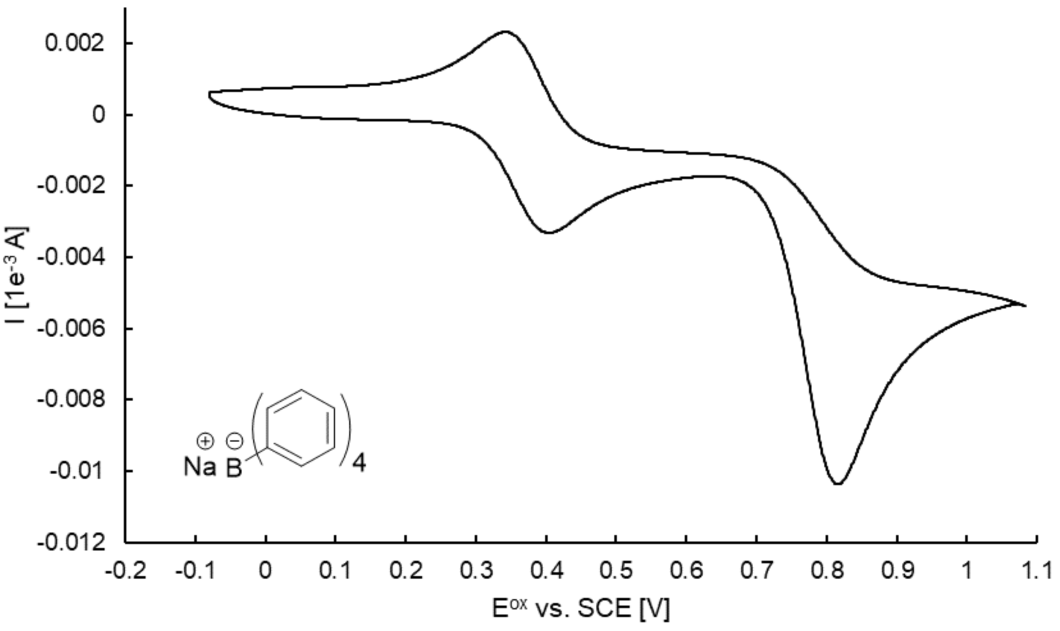

Figure 4: Cyclic voltammogram of $NaBPh_4$.

5. Theoretical Calculations

Calculations were performed at the equation-of-motion ionization potential coupled-cluster singles and doubles (EOM-IP-CCSD) level of theory and using density functional theory (DFT) with the ω B97X-D3 functional. The 6-31G* basis set was used in all calculations if not indicated otherwise. (EOM-IP)-CCSD calculations (Table 3) were performed for isolated molecules in gas phase, DFT calculations were performed in gas phase (Table 4) and additionally taking into account the solvent (acetonitrile) by means of the polarizable continuum (PCM) approach (Tables 5 and 6).[35] Non-equilibrium solvent effects upon ionization were either disregarded (Table 5) or taken into account by means of the state-specific approach (Table 6).[36] Molecular structures of the ATB anions were optimized at the ω B97X-D3/6-31G*/PCM level of theory.[37] Core electrons were frozen in all CCSD and EOM-IP-CCSD calculations.[38] All calculations were performed with the Q-Chem program package, release 5.0.[39]

The energy differences shown in Tables 3−6 support most of the trends observed in experiment. For the both ATB salts **2a**−**b**, the calculations agree with cyclic voltammetry measurements (see Section 4) that the species with the styryl group (**2b**) shows a lower oxidation potential than the tetraphenylborate anion. This effect is however nullified, when the phenyl substituents are exchanged for more electron-deficient *p*-fluorophenyl residues (**2a**), which results in a higher oxidation potential for this ATB salt compared to **2b** and an almost identical oxidation potential as the tetraphenylborate anion.

To characterize the change in the electronic structure upon oxidation of the ATB anions, spin and charge densities (Tables 7 and 8) were computed based on Mulliken population analysis.[40] Since this approach is known to suffer from a heavy basis-set dependence, partial charges were additionally computed using the ChElPG (Charges from the electrostatic potential on a grid) approach (Table 9).[41] These results illustrate that the single styryl moiety is selectively oxidized in all cases while the charge and spin densities of the other aromatic rings change only insignificantly. This fact is also visualized in Figure 5 and 6 by means of the spin densities of molecules **2a-b**. Notably, results do not change significantly for all other molecules when going from 6-31G* to 6-311G**.

---

[35] J. Tomasi; B. Mennucci; R. Cammi, *Chem. Rev.* **2005**, *105*, 2999.
[36] (a) M. Cossi; V. Barone, *J. Phys. Chem. A* **2000**, *104*, 10614. (b) Z.-Q. You; J.-M. Mewes; A. Dreuw; J. M. Herbert, *J. Chem. Phys.* **2015**, *143*, 204104.
[37] (a) J.-D. Chai; M. *J.* Head-Gordon, *Chem. Phys.* **2008**, *128*, 084106. (b) J.-D. Chai; M. Head-Gordon, *Phys. Chem. Chem. Phys.* **2008**, *10*, 6615. (c) S. Grimme; J. Antony; S. Ehrlich; H. Krieg, *J. Chem. Phys.* **2010**, *132*, 154104.
[38] I. Shavitt; R. J. Bartlett, Many Body Methods in Chemistry and Physics. MBPT and Coupled-Cluster Theory, *Cambridge University Press*, Cambridge, UK, **2009**.
[39] Y. Shao *et al.*, *Mol. Phys.* **2015**, *113*, 184.
[40] F. Jensen, Introduction to Computational Chemistry, *Wiley*, New York, USA, **1994**.
[41] (a) C. M. Breneman; K. B. Wiberg, *J. Comput. Chem.* **1990**, *11*, 361. (b) J. M. Herbert; L.D. Jacobson; K. U. Lao; M. A. Rohrdanz, *Phys. Chem. Chem. Phys.* **2012**, *14*, 7679.

*Table 3: Total energies (in atomic units) of closed-shell ATB anions (**2a-b**) and NABPh4 reference and the corresponding neutral radicals computed at the CCSD/6-31G* and EOM-IP-CCSD/6-31G* levels of theory, respectively. Energy differences (in eV) are also shown.*

ATB salt	E_t(Anionic) (a.u.)	E_t(Radical) (a.u.)	ΔE (eV)
$NaBPh_4$ reference	-948.477575	-948.3275427	4.08
**2a**	-1322.690426	-1322.534683	4.24
**2b**	-1025.630332	-1025.484670	3.96

*Table 4: Total energies (in atomic units) of closed-shell ATB anions (**2a-b**) and NABPh4 reference and the corresponding neutral radicals computed at the ω B97X-D3/6-31G* level of theory. Energy differences (in eV) are also shown.*

ATB salt	E_t(Anionic) (a.u.)	E_t(Radical) (a.u.)	ΔE (eV)
$KBPh_4$ reference	-951.253221	-951.094822	4.31
**2a**	-1326.271536	-1326.112552	4.33
**2b**	-1028.631710	-1028.481268	4.09

*Table 5: Total energies (in atomic units) of closed-shell ATB anions (**2a-b**) and NABPh4 reference and the corresponding neutral radicals computed at the ω B97X-D3/6-31G*/PCM level of theory. The solvent reaction field is equilibrated in all calculations. Energy differences (in eV) are also shown.*

ATB salt	E_t(Anionic) (a.u.)	E_t(Radical) (a.u.)	ΔE (eV)
$KBPh_4$ reference	-951.325714	-951.104965	6.01
**2a**	-1326.336968	-1326.136995	5.44
**2b**	-1028.704321	-1028.505684	5.41

*Table 6: Total energies (in atomic units) of closed-shell ATB anions (**2a-b**) and NABPh4 reference and the corresponding neutral radicals computed at the ω B97X-D3/6-31G*/PCM level of theory. The state-specific approach is used to describe non-equilibrium solvent effects upon ionization. Energy differences (in eV) are also shown.*

ATB salt	E_t(Anionic) (a.u.)	E_t(Radical) (a.u.)	ΔE (eV)
$KBPh_4$ reference	-951.325714	-951.075570	6.81
**2a**	-1326.336968	-1326.103329	6.36
**2b**	-1028.704321	-1028.472163	6.32

*Table 7: Spin densities of neutral ATB (**2a-b**) and NABPh4 reference radicals computed from Mulliken population analysis at the ω B97X-D3/6-31G*/PCM level of theory. The values represent the sums of the spin densities associated with the carbon atoms of the four aromatic rings.*

ATB salt	Spin densities Ar^1	Spin density Ar^2/vinyl
$KBPh_4$ reference	0.27/0.24/0.24/0.26	-
**2a**	0.00/0.03/0.06	0.37/0.61
**2b**	0.00/0.03/0.07	0.33/0.62

*Table 8: Differences in charge density between ATB (**2a-b**) and NABPh4 reference anions and neutral radicals computed from Mulliken population analysis at the ω B97X-D3/6-31G*/PCM level of theory. The values represent the sums of the charge density differences associated with the carbon atoms of the four aromatic rings.*

ATB salt	ΔCharge density Ar^1	ΔCharge density Ar^2/vinyl
$KBPh_4$ reference	0.09/0.09/0.09/0.10	-
**2a**	0.02/0.01/0.04	0.19/0.17
**2b**	0.02/0.01/0.03	0.18/0.17

*Table 9: Differences in charge density between ATB (**2a-b**) and NABPh4 reference anions and neutral radicals computed from charges from the electrostatic potential on a grid (ChElPG) at the ω B97X-D3/6-31G*/PCM level of theory. The values represent the sums of the charge density differences associated with the carbon atoms of the four aromatic rings.*

ATB salt	Charge density Ar^1	Charge density Ar^2/vinyl
$KBPh_4$ reference	-0.21, -0.20, -0.20, -0.19	-
**2a**	-0.02/-0.01/-0.05,	-0.29/-0.56
**2b**	-0.03/-0.01/-0.06	-0.28/-0.47

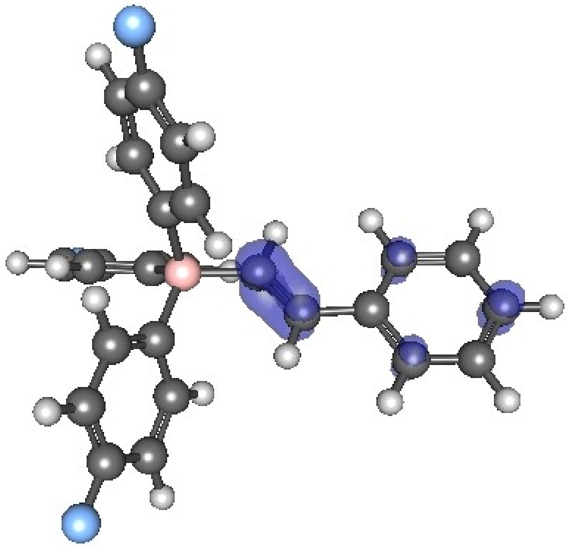

*Figure 5: Spin density for the neutral ATB radical (from **2a**) computed at the ω B97X-D3/6-31G*/PCM level of theory and plotted at an isovalue of 0.015.*

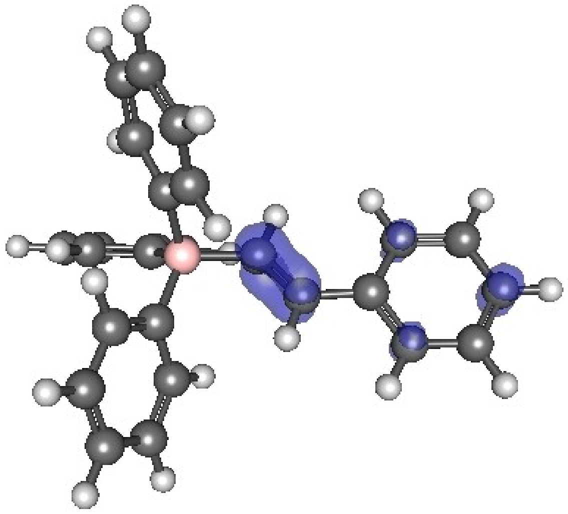

*Figure 6: Spin density for the neutral ATB radical (from **2b**) computed at the ω B97X-D3/6-31G*/PCM level of theory and plotted at an isovalue of 0.015.*

6. Single Crystal X-Ray Diffraction

Structure Determinations: The intensity data of **2a** (102 K) was collected on a Bruker D8 Venture TXS diffractometer using Mo-K_{α} radiation (λ = 0.71073 Å). The structures were solved by direct methods (SHELXT)[42] and refined by full-matrix least squares techniques against F_o^2 (SHELXL-2014/7)[43].

Supporting Information available: Crystallographic data have been deposited with the Cambridge Crystallographic Data Centre: CCDC-1964338 for **2a**. Copies of the data can be obtained free of charge: https://www.ccdc.cam.ac.uk/structures/.

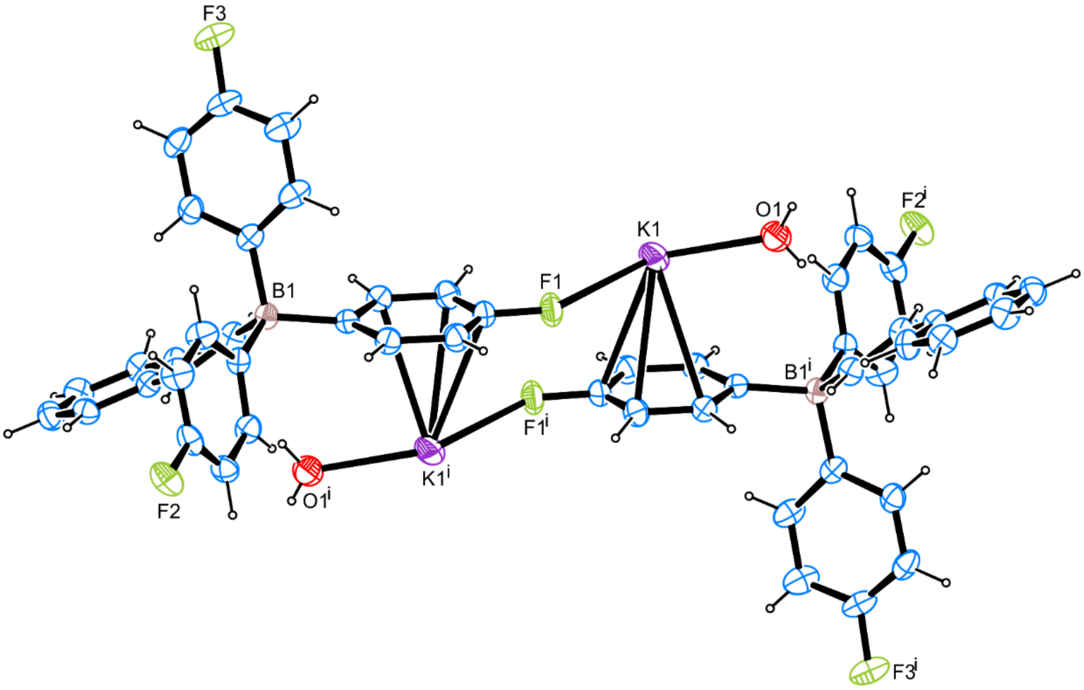

Figure 7: Crystal structure of ATB salt **2a**.

[42] G. M. Sheldrick, *Acta Cryst.* **2015**, A**71**, 3–8.

[43] G. M. Sheldrick, *Acta Cryst.* **2015**, C**71**, 3–8.

Details for X-ray data collection and structure refinement for compound **2a:**

	**2a**
net formula	$C_{26}H_{21.56}BF_3KO_{1.28}$
M_r/g mol^{-1}	461.42
crystal size/mm	0.070 × 0.050 × 0.030
T/K	102.(2)
radiation	MoKα
diffractometer	'Bruker D8 Venture TXS'
crystal system	triclinic
space group	'P -1'
a/Å	9.8924(4)
b/Å	10.0377(4)
c/Å	11.5787(4)
α/°	82.5670(10)
β/°	81.2960(10)
γ/°	88.2120(10)
V/Å^3	1126.86(7)
Z	2
calc. density/g cm^{-3}	1.360
μ/mm^{-1}	0.278
absorption correction	Multi-Scan
transmission factor range	0.96–0.99
refls. measured	19826
R_{int}	0.0309
mean σ(*I*)/*I*	0.0293
θ range	2.887–27.101
observed refls.	4217
x, y (weighting scheme)	0.0605, 1.4236
hydrogen refinement	mixed
refls in refinement	4955
parameters	302
restraints	0
$R(F_{obs})$	0.0566
$R_w(F^2)$	0.1499
S	1.054
shift/error$_{max}$	0.001
max electron density/e Å^{-3}	1.214
min electron density/e Å^{-3}	−1.115

H(C) constr, H(O1) refall
H(O2) not considered in refinement

The sof of O_2 has been refined freely and results in a value of 0.28.
The hydrogen atoms bound to this O could not be located and have not been considered in the refinement. This water-O-atom is not depicted in the figure above.
